# Supplementary material for: Terahertz generation and detection through gain-enhanced interband photomixing in quantum well structures
Source: Nat Commun. 2026 May 13;17:6247. doi: 10.1038/s41467-026-73080-6 (PMC13373183; doi:10.1038/s41467-026-73080-6)
Supplement: Supplementary file 1 — Supplementary Information [file 41467_2026_73080_MOESM1_ESM.pdf]

Supplementary Information

Terahertz Generation and Detection through Gain-Enhanced Interband Photomixing in Quantum Well Structures

Yifan Zhao[1,2], Shahed-E- Zumrat[1,2], Szu-An Tsao[1,2], Mona Jarrahi[1,2*]

[1]Electrical and Computer Engineering Department, University of California; Los Angeles, 90095, USA.

[2]California NanoSystems Institute, University of California; Los Angeles, 90095, USA.

*Corresponding author. Email: mjarrahi@ucla.edu

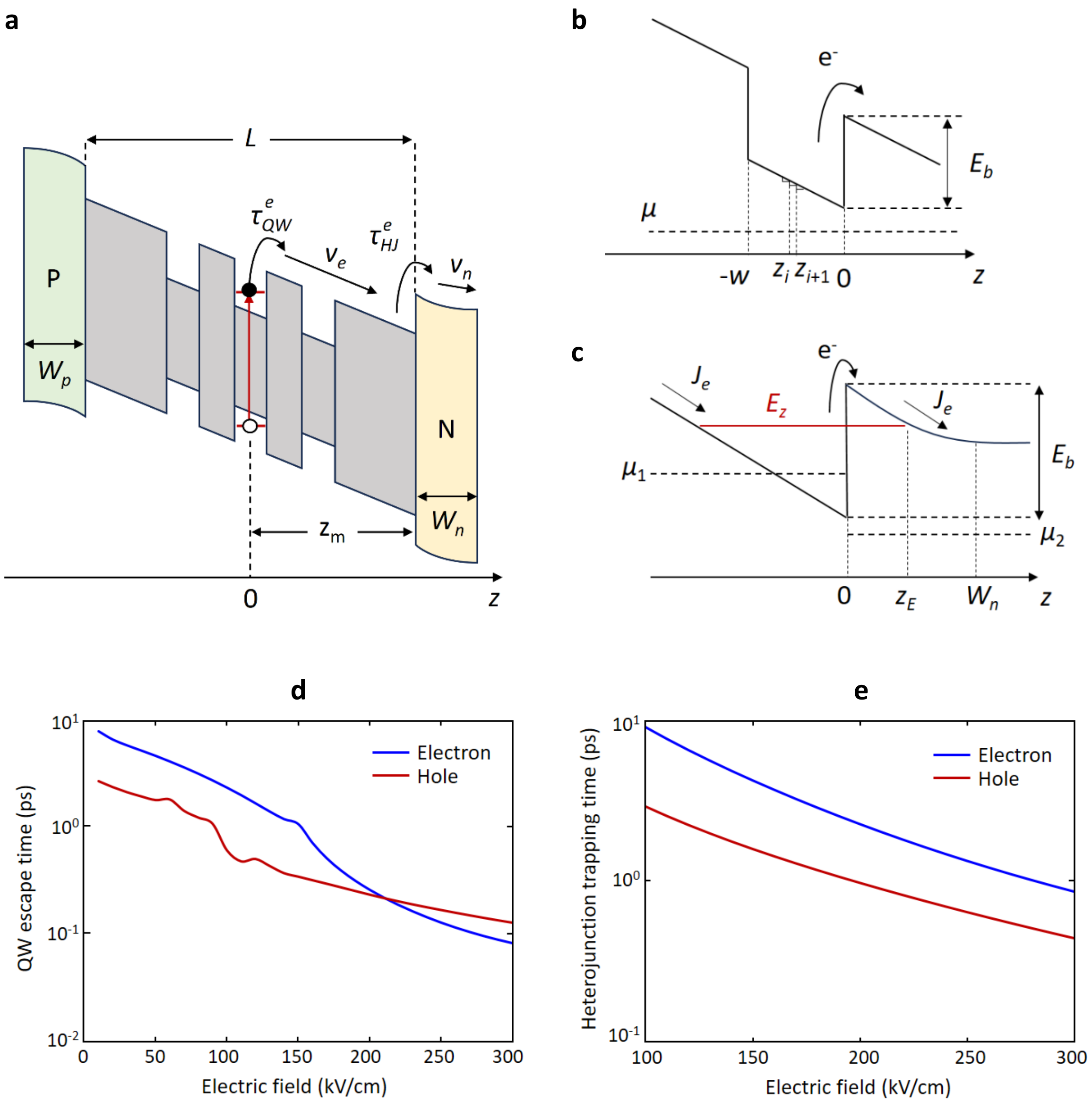

Fig. S1. Modeling of ultrafast carrier dynamics in a QW PIN photodiode reproduced from Zhao, Y. and Jarrahi, M., Journal of Applied Physics 139, 015701 (2026). A more detailed analysis is provided in Ref. 29 of the manuscript: Zhao, Y. & Jarrahi, M. Ultrafast carrier dynamics in multiple quantum well p-i-n photodiodes. J. Appl. Phys. 139, 015701 (2026). **a,** Illustration of the PIN structure with multiple QWs embedded in the intrinsic region. Photons are absorbed in the QWs and generate photocarriers. The photo-generated carriers need to escape from the QWs, then drift across the intrinsic region, may be temporarily trapped at the heterojunction interfaces; after escaping the heterojunction barrier, they traverse the depleted cladding layers, and are finally collected at the contact layers. The current generated by the mth QW superimposes with that of other QWs. Thus, the current contributions from all QWs are summed to obtain the total current. We

have used the Shockley-Ramo theorem to determine the current induced by the photocarrier motion. The total current is a sum of contributions from electrons and holes in all the QWs. For an optical impulse incident on the structure, the electron density in the mth QW can be expressed as:

$$N(t) = N_m e^{-t/\tau_{QW}^e} u(t) \qquad (S1-1)$$

where N_m is the electron-hole pair density at t = 0 and τ_{QW}^e is the electron escape time from the QW. After escaping the QW, the carriers drift at a saturation velocity v_e, through the intrinsic region with a density:

$$n(z,t) = \frac{N_m}{v_e \tau_{QW}^e} e^{-\left(t-\frac{z}{v_e}\right)/\tau_{QW}^e} u\left(t - \frac{z}{v_e}\right) \qquad (S1-2)$$

where z is the position measured from the center of the QW, $\tau_{trans}^e = \frac{z_m}{v_e}$ is the electron transit time to reach the heterojunction interface, and z_m is the distance between the mth well and the heterojunction interface. According to the Shockley-Ramo theorem, the induced current by electrons moving through the intrinsic region for the mth QW is:

$$I_{e,m,i}(t) = f_i \int_0^{z_m} q v_e S n(z,t)\, dz = \begin{cases} f_i q v_e S N_m \left(1 - e^{-\frac{t}{\tau_{QW}^e}}\right), & 0 < t < \tau_{trans}^e \\ f_i q v_e S N_m \left(1 - e^{-\frac{\tau_{trans}^e}{\tau_{QW}^e}}\right) e^{-\frac{t-\tau_{trans}^e}{\tau_{QW}^e}}, & t \geq \tau_{trans}^e \end{cases} \qquad (S1-3)$$

where S is the device area, $f_i = \frac{1/\varepsilon_i}{L/\varepsilon_i + W_n/\varepsilon_n + W_p/\varepsilon_p}$ is the weighting field in the intrinsic region, ε_i, ε_n and ε_p are the dielectric constants of the intrinsic, n-cladding, and p-cladding layers, W_n and W_p are the depletion width of the n- and p-cladding layers, and L is the intrinsic region thickness. The frequency response is obtained by taking the Fourier Transform of this current:

$$I_{e,m,i}(\omega) = \int I_{e,m,i}(t) e^{-j\omega t} dt = z_m f_i \frac{q S N_m}{1 + j\omega\tau_{QW}^e} sinc\left(\frac{\omega \tau_{trans}^e}{2}\right) e^{-\frac{j\omega\tau_{trans}^e}{2}} \qquad (S1-4)$$

This expression represents the induced current for electrons in the mth QW. We can write similar expressions for holes. After the carriers arrive at the heterojunction interface, they can be temporarily trapped due to the barrier height. The rate equation governing the electrons trapped at the heterojunction interface is used to analyze the dynamics of carrier trapping:

$$\frac{dN_t}{dt} = -\frac{N_t}{\tau_{HJ}^e} + n(z_m, t) v_e \qquad (S1-5)$$

where N_t is the trapped electron density, $n(z_m, t)$ is the electron density at the interface as a function of time, and τ_{HJ}^e is the heterojunction electron trap time. We obtain $N_t(t)$ by solving this equation:

$$N_t(t) = \frac{N_m}{1 - \frac{\tau_{QW}^e}{\tau_{HJ}^e}} \left(e^{-\frac{t-\tau_{trans}^e}{\tau_{HJ}^e}} - e^{-\frac{t-\tau_{trans}^e}{\tau_{QW}^e}} \right) u(t - \tau_{trans}^e) \qquad (S1-6)$$

After the carriers escape the heterojunction barrier, they traverse the depleted cladding region at saturation velocity v_n . The induced current by these electrons moving through the depleted n-cladding is:

$$I_{e,m,n}(t) = f_n q v_n S \int_{t-t_n}^{t} \frac{N_t(t)}{\tau_{HJ}^e} dt \qquad (S1-7)$$

where $t_n = \frac{W_n}{v_n}$ is the electron transit time in the depleted n-cladding layer, $f_n = \frac{1/\varepsilon_n}{L/\varepsilon_i + W_n/\varepsilon_n + W_p/\varepsilon_p}$ is the weighting field of the n-cladding layer. The frequency response is obtained by taking the Fourier Transform of this current:

$$I_{e,m,n}(\omega) = W_n f_n \frac{qSN_m}{1 + j\omega\tau_{QW}^e} \frac{1}{1 + j\omega\tau_{HJ}^e} sinc\left(\frac{\omega t_n}{2}\right) e^{-j\omega\left(\tau_{trans}^e + \frac{t_n}{2}\right)} \qquad (S1-8)$$

Total induced current is the summation of electron and hole currents generated in all QWs:

$$I(\omega) = \sum_m \left[I_{e,m,i}(\omega) + I_{e,m,n}(\omega) + I_{h,m,i}(\omega) + I_{h,m,p}(\omega) \right] \qquad (S1-9)$$

Here, $I_{h,m,i}$ is the current induced by holes originating from the mth well moving through the intrinsic region and $I_{h,m,p}$ is the current induced by the holes moving through the depleted p-cladding. **b,** The conduction band profile of a symmetric QW with barrier height E_b and width w under electric field along the z-axis. Under an applied electric field F, conduction band profile can be written as: $V(z) = -Fz$ within the well ($-w<z<0$) and $V(z) = E_b - Fz$ outside the well ($z < -w$ or $z > 0$). Under the influence of the electric field, electron states with energy below the barrier become quasi-bounded, allowing for tunneling with a finite probability. The combined tunneling and thermionic emission currents form the total electron escape current, with the escape time constant defined as $\tau_{QW}^e = \frac{N_{QW,e}}{J_e}$, where J_e is the electron escape current density and $N_{QW,e}$ is the 2D electron density in the well. Total escape current is calculated by integrating over all k-space states weighted by the Fermi distribution:

$$J_e = q \iint_{-\infty}^{\infty} \frac{dk_x dk_y}{(2\pi)^2} \left[\int_{k_{th}}^{\infty} f(E) \langle v_g^+(E_z) \rangle T^+(E_z) \frac{dk_z}{2\pi} \right] \qquad (S1-10)$$

where $f(E) = \frac{1}{e^{(E-\mu)/kT} + 1}$ is the Fermi-Dirac distribution, μ is the Fermi level, $\langle v_g^+(E_z) \rangle$ is the average group velocity in the $+z$ direction, and $T^+(E_z)$ is the transmission probability at the barrier. By reformulating in terms of energy:

$$J_e = q\int_0^{\infty} \frac{m_e}{2\pi\hbar^2} dE_t \left[\int_{E_{th}}^{\infty} f(E)\langle g_{1d}(E_z)\rangle\langle v_g^+(E_z)\rangle T^+(E_z) dE_z\right] \qquad (S1-11)$$

where m_e is the electron effective mass, $E = E_t + E_z$ is total energy decomposed into in-plane (E_t) and perpendicular (E_z) components, $\langle g_{1d}(E_z)\rangle$ is the average 1D density of states (DOS), $E_{th} = E_z(k_{th})$, and k_{th} is the threshold below which tunneling is negligible. Below the threshold, the energy states are well confined with discrete, step-like DOS which is similar to an un-biased QW. Above the threshold, states are broadened with continuous DOS and contribute to the escape current. A transmission line analogy is used to compute the 1D DOS, group velocity and transmission probability. The 2D electron density $N_{QW,e}$ is calculated by:

$$N_{QW,e} = w\int_0^{\infty} g_{3d}(E) f(E) dE \qquad (S1-12)$$

Here, $g_{3d}(E)$ is the 3D DOS which varies with energy. For electrons with energy below threshold, discrete DOS is given by:

$$\underset{E<E_{th}}{g_{3d}(E)} = \sum_{E_l<E} \frac{m_e}{\pi^2\hbar^2}(k_l - k_{l-1}) \qquad (S1-13)$$

where $k_l = \sqrt{2m_e E_l}/\hbar$ and $k_0 = 0$. For electrons with energy above the threshold, the continuous DOS is determined by

$$\underset{E>E_{th}}{g_{3d}(E)} = \sum_{E_l<E_{th}} \frac{m_e}{\pi^2\hbar^2}(k_l - k_{l-1}) + \int_0^{\infty} \frac{m_e}{2\pi\hbar^2} dE_t \int_{E_{th}}^{E} \langle g_{1d}(E_z)\rangle \delta(E - E_t - E_z) dE_z \quad (S1-14)$$

To determine QW escape time for holes, we assumed heavy and light holes are in equilibrium with a common quasi-Fermi level. The escape current is calculated separately for heavy and light holes then summed to obtain the total hole current density. **c,** Conduction band profile at the interface between the intrinsic layer and n-cladding layer. At the heterojunction interface carriers can escape via thermionic emission or tunneling through the barrier. Total current across the interface includes both tunneling and thermionic components. For a heterojunction with a conduction band barrier E_b, the conduction band is described by $E_c(z) = -Fz$ for z < 0 and $E_c(z) = E_b + \frac{1}{2}FW_n\left[1 - \left(1 - \frac{z}{W_n}\right)^2\right]$ for 0 < z < W_n. The electron transmission probability through the barrier is:

$$T(E_z) = \begin{cases} \exp\left(-\frac{2}{\hbar}\int_0^{z_E} [2m_{e2}(E_c(z) - E_z)]^{1/2} dz\right), & if\ E_{min} \le E_z \le E_c(0^+) \\ 1, & if\ \ E_z \ge E_c(0^+) \end{cases} \qquad (S1-15)$$

where $E_c(z)$ is the conduction band profile, E_{min} = max($E_c(0^-)$, $E_c(W_n)$) is the minimum energy level for electron tunneling, z = 0 is the position of the heterojunction interface, z_E is the position

where $E_c(z_E) = E_z$, and m_{e2} is the electron effective mass in the cladding layer. Total current can be determined:

$$J_e = J_{tunn} + J_{therm} = \frac{AT}{k}\int_{E_{min}}^{E_c(0^+)} f'(E_z)T(E_z)\,dE_z + \frac{AT}{k}\int_{E_c(0^+)}^{\infty} f'(E_z)T(E_z)\,dE_z \quad (S1-16)$$

where $A = \frac{4\pi q m_{e1} k^2}{h^3}$ is the Richardson constant, m_{e1} is the effective electron mass on the intrinsic side, and $f'(E_z) = \ln\left(1 + \exp[(\mu_1 - E_z)/kT]\right)$ is the Fermi distribution integrated over in-plane states. 2D density of trapped electrons at the heterojunction interface is calculated as:

$$N_{HJ,e} = \int_0^{-\infty} N_c e^{-(E_c(z)-\mu_1)/kT} dz \quad (S1-22)$$

where N_c is the effective conduction band density of states in the intrinsic region, $\tau_{HJ}^e = \frac{N_{HJ,e}}{J_e}$ is the electron trapping time at the heterojunction, and $\tau_{HJ}^h = \frac{N_{HJ,h}}{J_h}$ is the hole trapping time, which is calculated similarly. **d,** The calculated electron and hole escape times from the QWs and **e,** the calculated trapping times at the heterojunction interfaces for the GaAs/AlGaAs QW PIN photodiode heterostructure used for fabricating the terahertz source/detector prototypes. The calculation details are provided in Ref. 29 of the manuscript: Zhao, Y. & Jarrahi, M. Ultrafast carrier dynamics in multiple quantum well p-i-n photodiodes. J. Appl. Phys. 139, 015701 (2026). Under increasing reverse bias, the electric field across the intrinsic region becomes stronger, tilting the potential barriers and enhancing carrier tunneling out of the wells. As a result, the carrier escape time from the QWs (τ_{QW}) becomes shorter at higher bias voltages. Similarly, the carrier transit time (τ_{trans}) decreases with reverse bias because carriers are accelerated more rapidly and quickly reach their saturation velocity, reducing the time required to traverse the depletion region. The heterostructure composition and layer thickness also play a central role. Higher barrier heights set by material composition (e.g., higher Al content in AlGaAs) and thicker barriers increase confinement, leading to longer escape times, while thinner wells and barriers, along with lower band offsets, reduce the escape time by facilitating tunneling. Effective mass differences between materials further influence escape rates by modifying the tunneling probability. The total thickness of the intrinsic region directly determines the transit distance; thus, thicker intrinsic layers increase t_{trans}, whereas thinner regions reduce it. In addition, material-dependent saturation velocities and the presence of heterojunction barriers (which can temporarily trap carriers) further modulate the effective transit time. Overall, higher reverse bias and thinner, lower-barrier heterostructures lead to shorter τ_{QW} and τ_{trans}, while thicker layers and higher band offsets increase these characteristic times.

Material	Mole Fraction (x)	Thickness (nm)	Type	Doping level (cm^{-3})	Description
GaAs		200	p-doped(C)	$>2\times10^{19}$	P-contact
Al(x)GaAs	0.55 to 0.05	50	p-doped(C)	$>3\times10^{18}$	
Al(x)GaAs	0.55	1250	p-doped(C)	1×10^{18}	P-cladding
GaIn(x)P	0.49	10	p-doped(Zn)	7×10^{17}	Etch stop
Al(x)GaAs	0.55	300	p-doped(C)	6×10^{17}	P-cladding
Al(x)GaAs	0.3	40	Undoped		Barrier
Al(x)GaAs	0.08	5.5	Undoped		QW
Al(x)GaAs	0.3	6	Undoped		Barrier
Al(x)GaAs	0.08	5.5	Undoped		QW
Al(x)GaAs	0.3	6	Undoped		Barrier
Al(x)GaAs	0.08	5.5	Undoped		QW
Al(x)GaAs	0.3	40	Undoped		Barrier
Al(x)GaAs	0.55	1500	n-doped(Si)	1×10^{18}	N-cladding
Al(x)GaAs	0.05 to 0.55	50	n-doped(Si)	2×10^{18}	N-cladding
GaAs		1000	n-doped(Si)	2×10^{18}	N-contact
SI-GaAs			Undoped		Substrate

Fig. S2. The GaAs/AlGaAs QW PIN photodiode heterostructure used for fabricating the terahertz source/detector prototypes is a commercially available design typically utilized in semiconductor lasers operating at an approximate wavelength of 800 nm. This structure was grown by Xiamen Powerway Advanced Material Co., Ltd (PAM-XIAMEN). From bottom to top, the structure comprises: a 1-µm-thick, highly doped n+ GaAs contact layer grown on a semi-insulating (SI) GaAs substrate; a 50-nm-thick graded AlGaAs layer, followed by a 1.5-µm-thick AlGaAs n-cladding layer; a 108.5-nm-thick intrinsic region; a 1.5-µm-thick AlGaAs p-cladding layer, which includes a 10-nm-thick lattice-matched GaInP etch stop layer positioned 300 nm above the intrinsic region; and another 50-nm-thick graded AlGaAs layer and a 200-nm-thick, highly doped p+ GaAs contact layer. Within the intrinsic region, there are three pairs of QWs, each consisting of a 5.5-nm-thick $Al_{0.08}Ga_{0.92}As$ well layer and a 6-nm-thick $Al_{0.3}Ga_{0.7}As$ barrier layer, flanked by a 40-nm-thick $Al_{0.3}Ga_{0.7}As$ layer on each side. Although this wafer structure enables high-performance terahertz transmitter and receiver functionality, there remains potential for optimization to enhance its suitability for integrated terahertz optoelectronic applications.

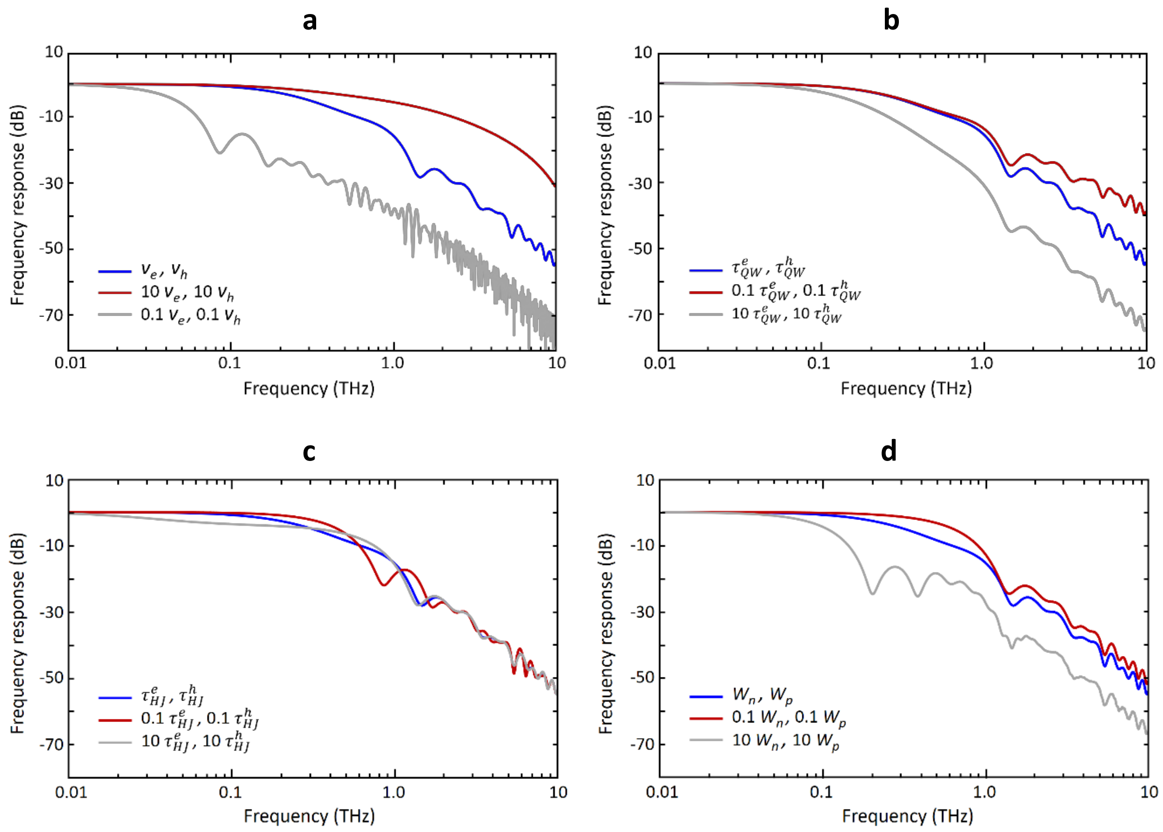

Fig. S3. Frequency response of the GaAs/AlGaAs QW PIN photodiode heterostructure used for fabricating the terahertz source/detector prototypes reproduced from Zhao, Y. and Jarrahi, M., Journal of Applied Physics 139, 015701 (2026). This frequency response is calculated using the model described in Fig. S1 and includes the impact of the electron and hole escape times from the QWs (τ_{QW}^e and τ_{QW}^h), electron and hole transit times through the intrinsic region (τ_{trans}^e and τ_{trans}^h), electron and hole trap times at the heterojunction interface (τ_{HJ}^e and τ_{HJ}^h), and excludes the impact of the device RC time constant, which is described in Figs. S4 and S5. The blue plot illustrates the frequency response based on the electron/hole saturation velocities of 0.72×10^7 / 0.8×10^7 cm/s through the depletion region [30, 31], as well as the calculated QW electron/hole escape times of 0.09/0.13 ps and heterojunction electron/hole trap times of 0.98/0.48 ps at an applied field of 280 kV/cm (as shown in Fig. S1 d, e). For frequencies between 0.1 and 1 THz, the frequency response exhibits a 20 dB/decade slope due to the slow transit time of ~1 ps. At higher frequencies, the slope increases to 40 dB/decade as the QW escape time (~0.1 ps) introduces an additional bottleneck. The calculation details are provided in Ref. 29 of the manuscript: Zhao, Y. & Jarrahi, M. Ultrafast carrier dynamics in multiple quantum well p-i-n photodiodes. J. Appl. Phys. 139, 015701 (2026). **a,** The impact of carrier transit time on the frequency response is studied by varying the carrier saturation velocity, while maintaining the geometry of the QW PIN structure,

illustrating the $sinc^2\left(\frac{\omega t_{trans}}{2}\right)$ dependence predicted by the theoretical model described in Fig. S1. Reducing the thickness of the intrinsic region while maintaining the same QW structures would decrease the carrier transit time and reduce the frequency roll-off. **b,** The impact of QW escape time on the frequency response is studied, illustrating the $(1 + j\omega\tau_{QW})^{-1}$ dependence predicted by the theoretical model. **c**, The impact of heterojunction trap time on the frequency response is studied, indicating a negligible dependence. This is because the heterojunction trap time only affects the current contribution from the photocarriers moving inside the depleted n- and p-cladding layers with depletion widths W_n and W_p, respectively. In the structure studied, the doping level is high enough to keep the depletion width in the cladding layer relatively small compared to the intrinsic region, resulting in minimal effect on the frequency response. **d,** The impact of cladding layer doping on the frequency response is studied. Using lighter doping in the cladding layers extends the depletion width, which increases the carrier transit time and results in a steeper frequency roll-off.

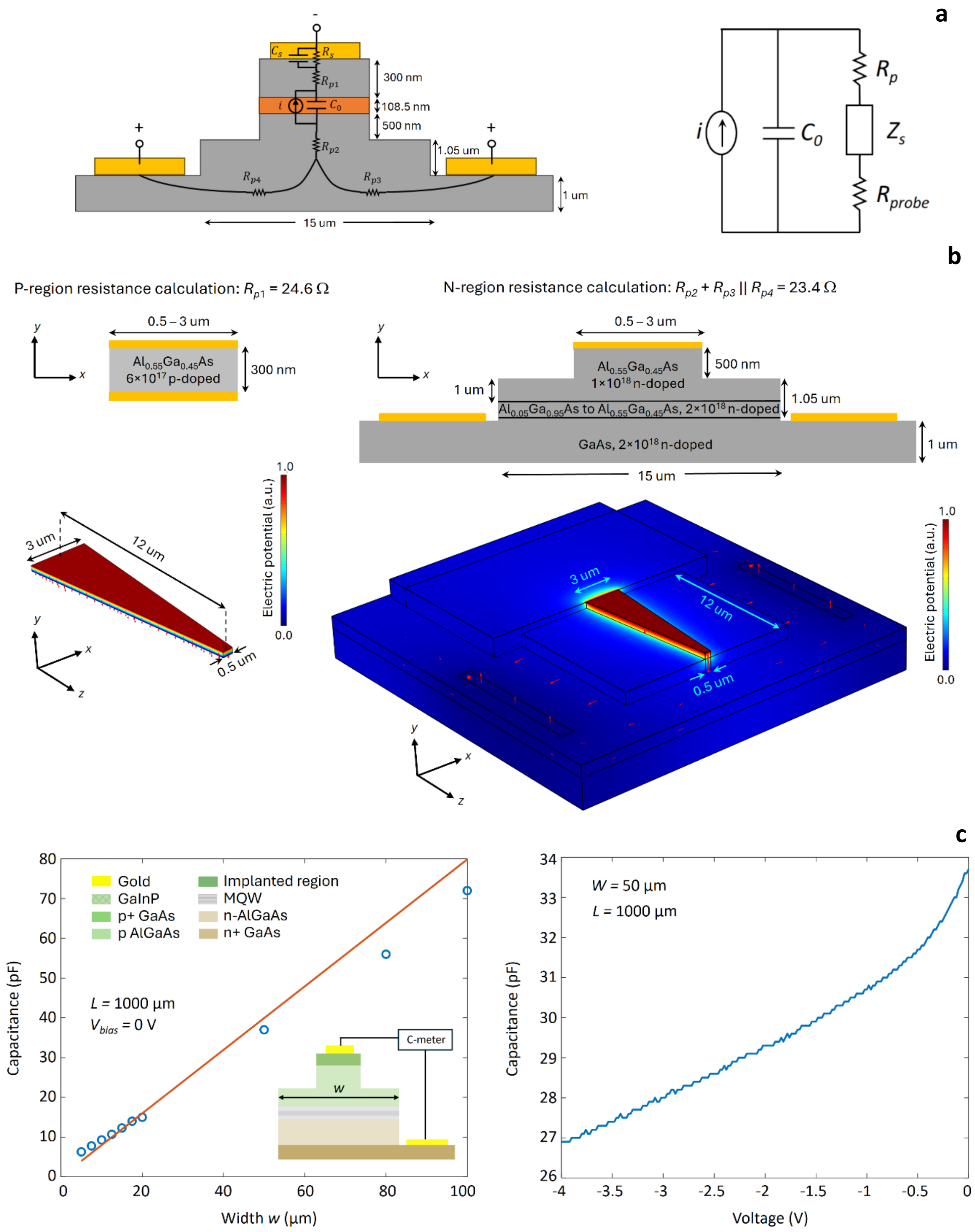

Fig. S4. a, The equivalent circuit model for the photomixer in terahertz generation mode is represented with the generated photocurrent modeled as a current source $i = I_{DC} + i_{THz}\cos(2\pi f_{beat} t)$, where I_{DC} and i_{THz} are the amplitudes of the DC and terahertz components of the photocurrent. Here, f_{beat} represents the terahertz beat frequency of the two optical tones

pumping the photomixer. The terahertz photocurrent component is given by $i_{THz} \cong i_{DC}.sinc(\pi f_{beat}\tau_{trans})\left(1+j2\pi f_{beat}\tau_{QW}\right)^{-1}$, where τ_{trans} is the effective carrier transit time from the QWs to the P/N layers, and τ_{QW} is the effective carrier escape time from the QWs. The current source is in parallel with the PIN diode depletion region capacitance C_0 and this combination is in series with the 50 Ω resistance of the GSG probe, R_{probe}, the parasitic resistance R_p from the n and p regions, and the Schottky contact impedance Z_s. The terahertz current passing through the load resistance is thus: $i_L = i_{THz}\left[1+j2\pi f_{beat}C_0(R_{probe}+R_p+Z_s)\right]^{-1} \equiv i_{THz}[1+j2\pi f_{beat}\tau_{RC}]^{-1}$ and the generated terahertz power is $\frac{1}{2}\left|i_L^2\right|R_{probe}$, where τ_{RC} represents the photomixer RC time constant for the designed photomixer shown in Fig. 2. **b,** The parasitic resistance R_p from the n and p regions is calculated using COMSOL. The DC current flowing through the p and n regions is simulated separately under a DC voltage and the corresponding resistance values for the p and n regions are calculated as R_{p1} = 24.6 Ω and $R_{p2} + R_{p3} \| R_{p4}$ = 23.4 Ω, with a total parasitic resistance of R_p = 48 Ω. **c**, The PIN diode depletion region capacitance C_0 is determined experimentally. Due to the small photomixer active area, it is not feasible to directly measure the capacitance with a capacitance meter. Instead, we fabricated 1-mm-long ridge waveguide samples with the cross-section shown in the inset and measured their capacitance using an HP 4280A capacitance meter. The width, w, in these samples varies from 5 to 100 μm across different samples. The left graph shows the measured capacitance values (blue data points) as a function of width at a 0 V bias voltage. The results are in close agreement with the estimated parallel plate capacitance (red curve), $\varepsilon Lw/t$, where $\varepsilon \approx 12\varepsilon_0$ is the average permittivity of the PIN depletion region, L = 1 mm, and t is the calculated depletion region thickness at 0 V bias (133 nm). The right graph shows the measured capacitance values as a function of reverse bias voltage for a device with a width of 50 μm. The device capacitance decreases with increasing the reverse bias voltage as the depletion region thickness increases. Finally, we extract the capacitance value of C_0 = 11.8 fF for our photomixer at a 3 V reverse bias by scaling the experimentally measured capacitance values according to the photomixer active area.

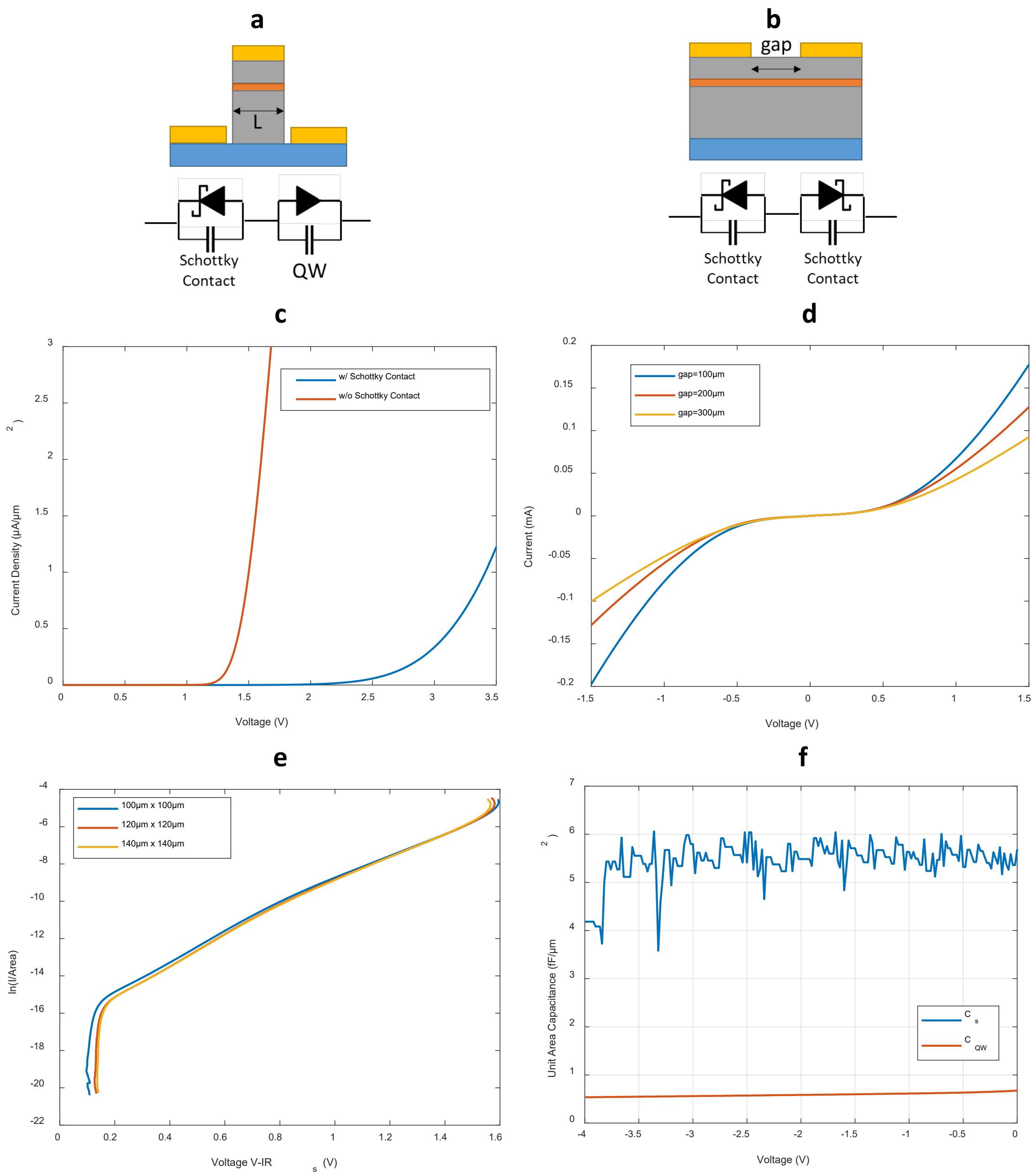

Fig. S5. Schottky contact circuit model: Since we etched away the p-type layers above the etch stop layer and deposited the photomixer top contact directly on the etch stop layer to minimize the parasitic resistance of the photomixer, the Schottky contact impedance plays a role in the frequency response of the photomixer. We extracted the capacitance and resistance of the formed Schottky contact using specific test structures shown in **a** and **b**. For the first test structure, we deposited 10/300 nm Cr/Au on the etch stop layer and etched mesas of various sizes. For the bottom contact, we used the same AuGe/Au ohmic contact as in the photomixer. The equivalent circuit model for this test structure is the series connection of the QW diode and the Schottky diode. The second test

structure consists of two Schottky contacts with different gap sizes on a mesa with a 200 μm width. Since the n-side is isolated from the p-side by the depletion region, the equivalent circuit model includes two Schottky diodes connected back-to-back. Both test structures were annealed at 380°C for 30 seconds, a requirement for the AuGe/Au ohmic contact. The IV characteristics of the two test structures are shown in **c** and **d**. When the two Schottky diodes are connected back-to-back, the threshold voltage of the entire structure is dominated by the reverse-biased diode. A threshold voltage of approximately 0.7 V is calculated from the IV characteristics of the second test structure. As illustrated in the IV characteristics of the first test structure, adding the Schottky contact to the QW PIN diode increases the turn-on voltage of the entire structure by approximately 1 V and reduces the current density. This behavior is expected due to the opposite polarity of the Schottky contact relative to the QW diode. As a result, the Schottky contact will be forward-biased when the photomixer is reverse-biased, which has a minor impact on the photomixer operation. To extract the IV characteristics of the Schottky contact, we used the slope of the IV curve to calculate the parasitic resistance and subtracted the IR voltage drop for the first test structure and a pure QW diode with a top ohmic contact. This approach allows us to isolate the IV characteristics of a pure QW diode with a top ohmic contact and a QW diode with a top Schottky contact. By subtracting the voltage across the QW diode at the same current density, we calculated the voltage across the Schottky contact and extracted the IV characteristics of the Schottky contact. The extracted IV characteristics of the Schottky contact from test structures with three different mesa sizes are shown in **e**, demonstrating excellent agreement across all results. To account for the barrier lowering effect under large reverse bias voltages, a Schottky contact can be modeled as:

$$I = I_0 e^{\frac{eV}{nkT}} \left[1 - e^{-\frac{eV}{kT}}\right] \qquad (S5-1)$$

where I_0 is the reverse-bias saturation current, V is the applied voltage, k is the Boltzmann constant, T is the temperature, and n is the ideality factor. When $|V| \gg kT/e$, we have

$$I = I_0 \exp\left(\frac{\left(\frac{1}{n} - 1\right) eV}{kT}\right) \qquad (S5-2)$$

$$ln\, I = ln\, I_0 + \frac{e(V - IR_s)}{kT}\left(\frac{1}{n} - 1\right)$$

Therefore, I_0 and n can be extracted from the results above and we calculate $I = \frac{5.7pA}{\mu m^2}[\exp\left(\frac{eV}{1.27kT}\right) - 1]$ for the Schottky contact. Under a DC photocurrent of 1 mA, this corresponds to an AC resistance of $R_s = dV/dI \approx 32.8\Omega$, which is independent of the device area. To compare this with the case where the Schottky contact is absent — meaning the p-type layers

above the etch stop are not removed, and the p+ GaAs layer still has an ohmic contact — we calculated the ohmic resistance of the p-type layers to be 60 Ω, which is approximately twice the resistance of the Schottky contact. We also characterized the Schottky contact capacitance by measuring the capacitance of the first test structure under different reverse bias voltages. The measured capacitance corresponds to the series combination of the Schottky contact capacitance, C_s, and the depletion region capacitance, C_{QW}, given by the equation $C = 1/(\frac{1}{C_{QW}} + \frac{1}{C_s})$. Since the Schottky junction is forward-biased when the QW diode is reverse-biased, most of the reverse bias voltage drops across the depletion region capacitance. Using the depletion region capacitance determined in Fig. S4, we solved for the Schottky contact capacitance C_s. The results, shown in **f**, reveal a constant Schottky contact capacitance of 5.5 fF/μm² as a function of the applied voltage, as theoretically expected. To summarize, the Schottky contact impedance is given by:

$$Z_s = \frac{32.8\Omega}{1 + j2\pi f_{beat} \times 32.8\Omega \times 5.5\text{fF} \cdot \mu\text{m}^{-2} * Area(\mu\text{m}^2)} \qquad (S5-3)$$

Using the extracted Schottky contact characteristics and the capacitance/resistance parasitic values $C_0 = 11.8$ fF and $R_p = 48$ Ω calculated in Fig. S4, the effective RC time constant of the photomixer is calculated as $\tau_{RC} \approx 1.55$ ps, where $1 + j2\pi f_{beat}\tau_{RC} \approx 1 + j2\pi f_{beat} C_0(R_{probe} + R_p + Z_s)$ in the 0.1-0.5 THz frequency range used in this work.

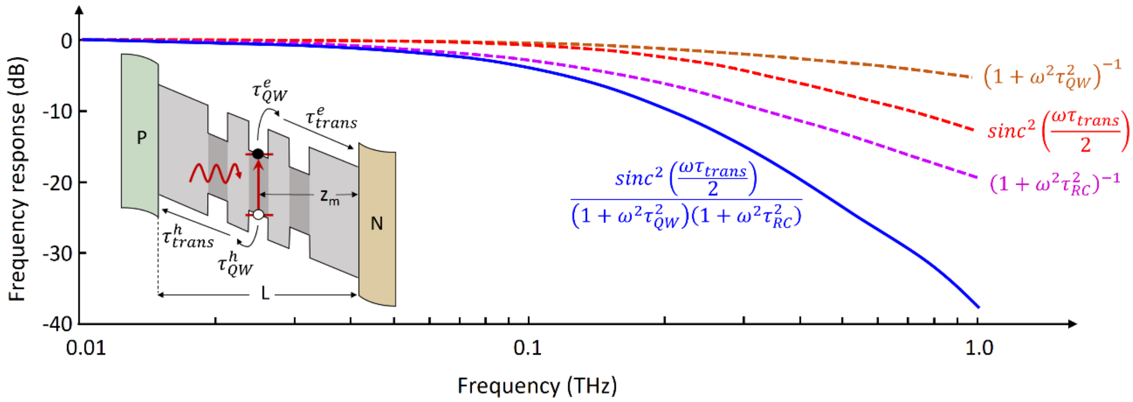

Fig. S6. The theoretically predicted frequency response of the QW PIN photomixer, which accounts for the ultrafast carrier dynamics (calculated in Figs. S1 and S3) and the photomixer RC time constant (calculated in Figs. S4 and S5).

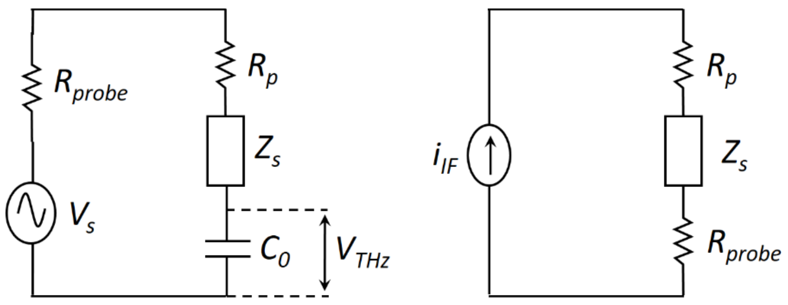

Fig. S7. The equivalent circuit model for the photomixer in terahertz detection mode includes a voltage source V_s and the 50 Ω resistance of the GSG probe, R_{probe}, to represent the terahertz signal input. These components are in series with the parasitic resistance R_p from the n and p regions, the Schottky contact impedance Z_s, and the capacitance C_0 of the PIN diode depletion region. For a received terahertz power $P_{THz} = \frac{V_s^2}{8R_{probe}}$, the induced voltage across the intrinsic region is calculated as $V_{THz} = V_s\left[1 + j2\pi f_{beat}C_0(R_{probe} + R_p + Z_s)\right]^{-1}$. As described previously, the photocurrent of the photomixer has both DC and terahertz components, represented by $i = I_{DC} + i_{THz}\cos(2\pi f_{beat}t)$, where $i_{THz} \cong i_{DC}.\, sinc(\pi f_{beat}\tau_{trans})\left(1 + j2\pi f_{beat}\tau_{QW}\right)^{-1} = i_{DC}.\, H_{carrier}(2\pi f_{beat})$. In terahertz detection mode, the voltage applied to the intrinsic region, $V_{DC} + V_{THz}\cos(2\pi f_{THz}t)$, consists of a DC component from the device's DC bias and a terahertz component, V_{THz}, induced across the intrinsic region of the photomixer at frequency f_{THz}. Assuming $V_{THz} \ll V_{DC}$, the induced photocurrent is given by $i = I_{DC} + I_{DC} * H_{carrier}(V_{DC} + V_{THz}\cos(2\pi f_{beat}t)) * \cos(2\pi f_{THz}t)$, approximated as $I_{DC} + I_{DC} * [H_{carrier}(V_{DC}) + H'_{carrier}(V_{DC}) * V_{THz}\cos(2\pi f_{THz}t)] * \cos(2\pi f_{beat}t)$, yielding an intermediate frequency (IF) component of $\frac{1}{2}I_{DC} * H'_{carrier}(V_{DC}) * V_{THz}\cos|2\pi f_{beat}t - 2\pi f_{THz}t|$. Thus, the magnitude of the IF current is $i_{IF} = \frac{1}{2}I_{DC}V_s * H'_{carrier}(V_{DC})\left[1 + j2\pi f_{beat}C_0(R_{probe} + R_p + Z_s)\right]^{-1}$. The conversion gain of the terahertz detector, defined as the down-converted IF power, $\frac{1}{2}i_{IF}^2R_{probe}$, divided by the received terahertz power, P_{THz}, is given by $[I_{DC}R_{probe} * H'_{carrier}(V_{DC})]^2\left[1 + j2\pi f_{beat}C_0(R_{probe} + R_p + Z_s)\right]^{-2}$, where $H'_{carrier}(V_{DC})$is proportional to $sinc(\pi f_{beat}\tau_{trans})\left(1 + j2\pi f_{beat}\tau_{QW}\right)^{-1}$.

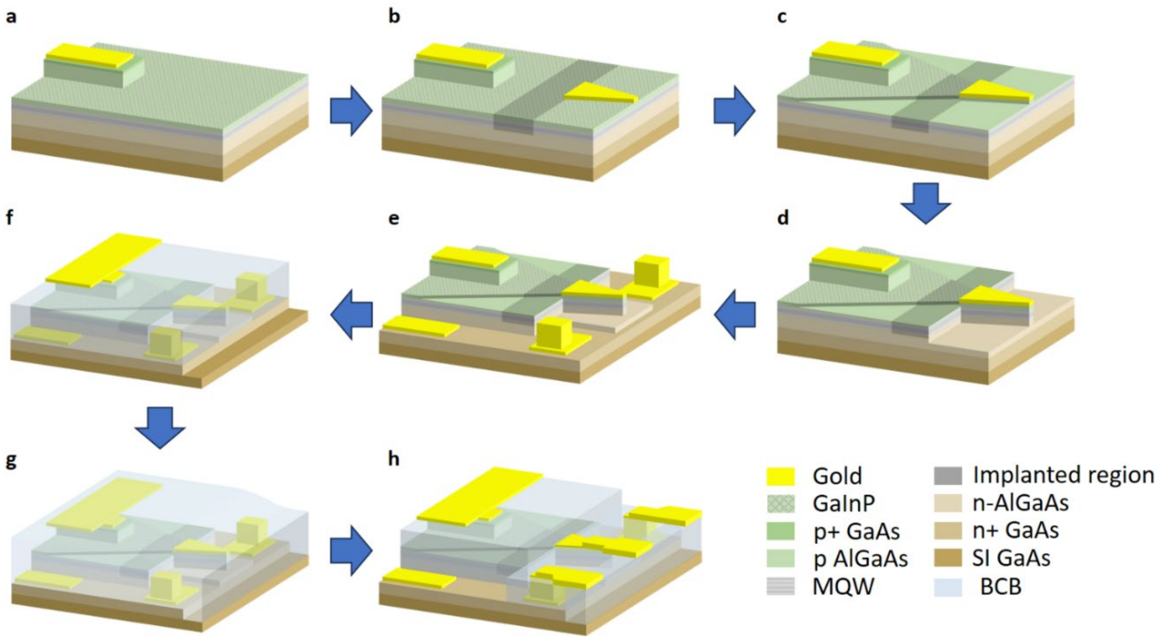

Fig. S8. The fabrication process for the terahertz source/detector prototype, which generates and detects terahertz signals via photomixing in a GaAs/AlGaAs QW PIN photodiode, involves the following steps: **a**, Deposition of the SOA top contact and etching of the SOA waveguide. **b**, Deposition of the photomixer top contact followed by ion implantation. **c**, Etching of the shallow taper. **d**, Etching of the photomixer waveguide. **e**, Exposure of the n+ GaAs layer and deposition of the bottom contacts. **f**, Spin coating with BCB (benzocyclobutene), followed by etch-back of BCB to allow deposition of the connection pad for the SOA top contact, and etching of the n+ GaAs in the GSG (ground-signal-ground) pad area. **g**, Spin coating with BCB again. **h**, Etch-back of BCB, deposition of GSG probing pads, and exposure of the SOA contact.

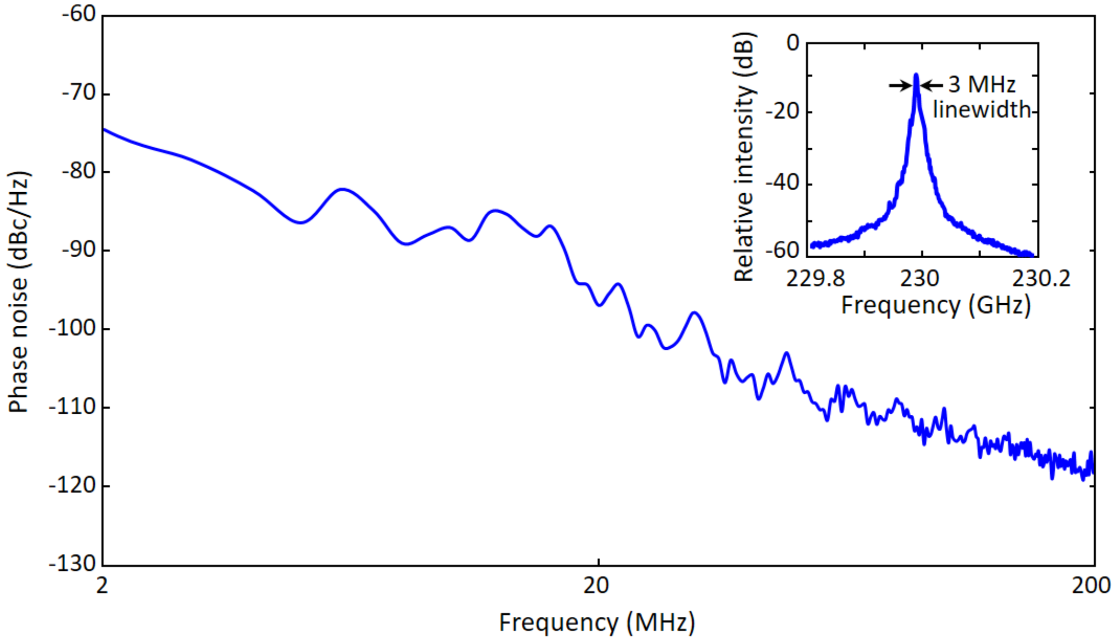

Fig. S9. Phase noise of the generated signal at 230 GHz, calculated from the measured signal spectrum shown in the inset.

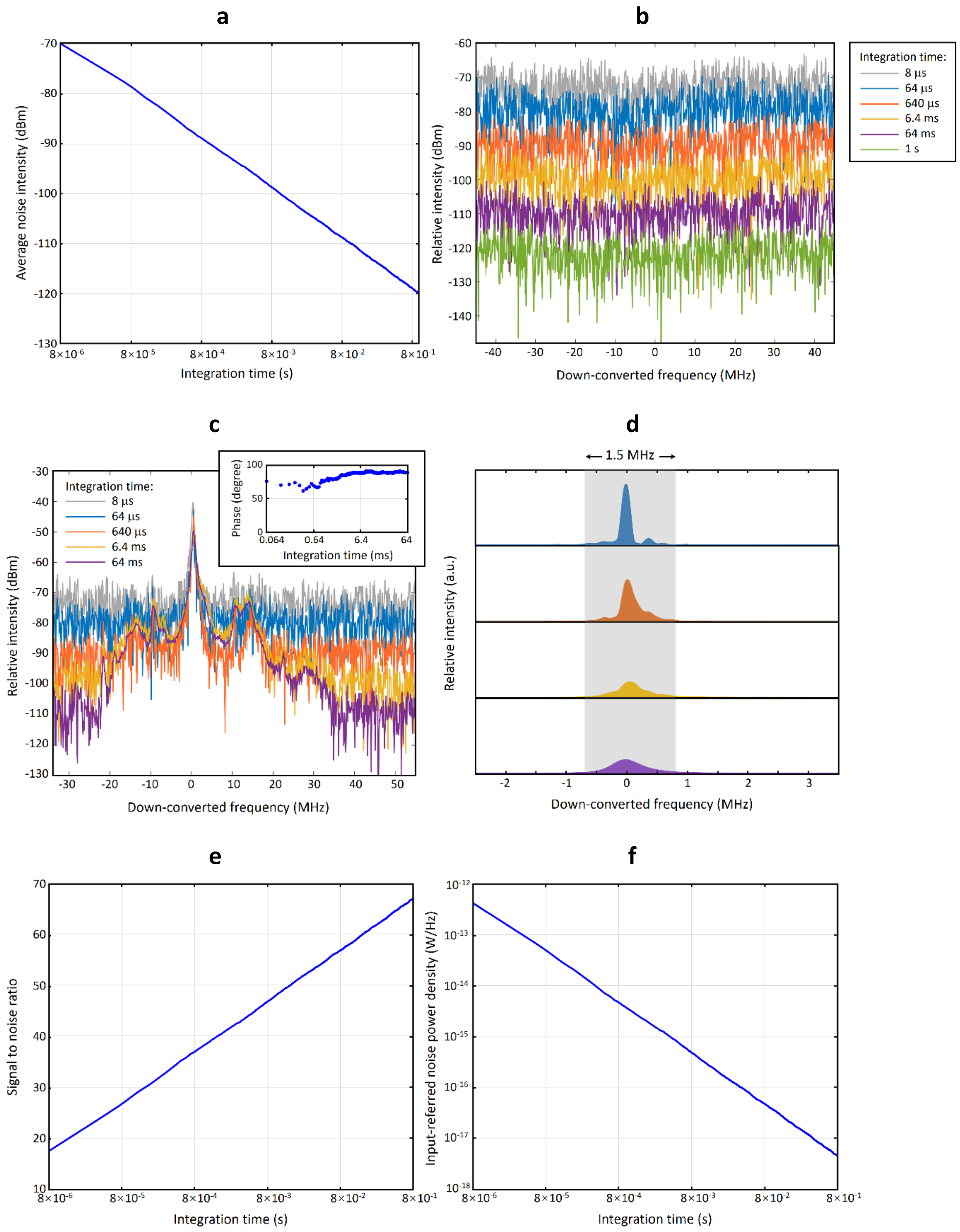

Fig. S10. Characteristics of the down-converted terahertz spectrum using spectral lock-in detection. **a,** Average noise intensity of the down-converted spectrum as a function of integration time, showing a noise reduction rate of 10 dB per decade. **b,** Down-converted

spectrum in the absence of a terahertz input for integration times ranging from 8 μs to 1 s. The 8 μs data correspond to measurements captured over an 8 μs time window without applying lock-in detection. **c,** Down-converted spectrum of a 240 GHz input tone at –14.5 dBm for integration times from 8 μs to 64 ms. Inset shows the phase of the resolved spectra at 240 GHz through spectral lock-in detection as a function of integration time. **d,** Down-converted signal power within a 1.5 MHz detection bandwidth remains constant for integration times up to 64 ms, despite IF spectral broadening caused by pump laser wavelength fluctuations. **e,** Signal-to-noise ratio (SNR) of the down-converted 240 GHz tone at –14.5 dBm, calculated as the ratio of signal power to noise power within a 1.5 MHz bandwidth, for integration times ranging from 64 μs to 1 s. **f,** Calculated input-referred noise power density, defined as the input power required to yield an SNR of 1 in a 1 Hz bandwidth ($\frac{-14.5\ dBm - SNR}{1.5\ MHz}$).

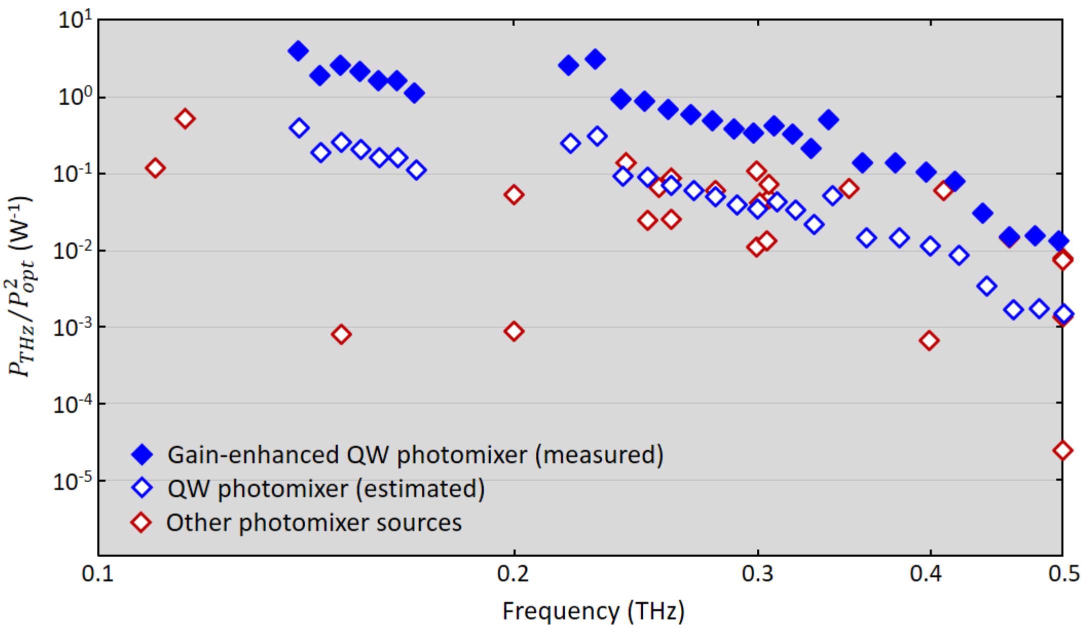

Fig. S11. The impact of the SOA gain on the terahertz generation efficiency is evaluated by comparing the efficiency figure of merit P_{THz}/P_{opt}^2 at the measured input optical power to the SOA (5 mW) and the estimated input optical power to the photomixer. The estimated input optical power to the photomixer is calculated as $\frac{1}{\eta}\frac{hc}{q\lambda}I_{ph} = 16.3$ mW, where h is the Planck's constant, c is the speed of light, q is the electron charge, λ is the optical wavelength of 809 nm, η is the photomixer quantum efficiency, estimated to be 56.5% from the electromagnetic simulations (Fig. 2b), and I_{ph} = 6 mA is the photomixer photocurrent. Based on these estimates, the SOA gain is found to enhance the terahertz generation efficiency by approximately an order of magnitude.

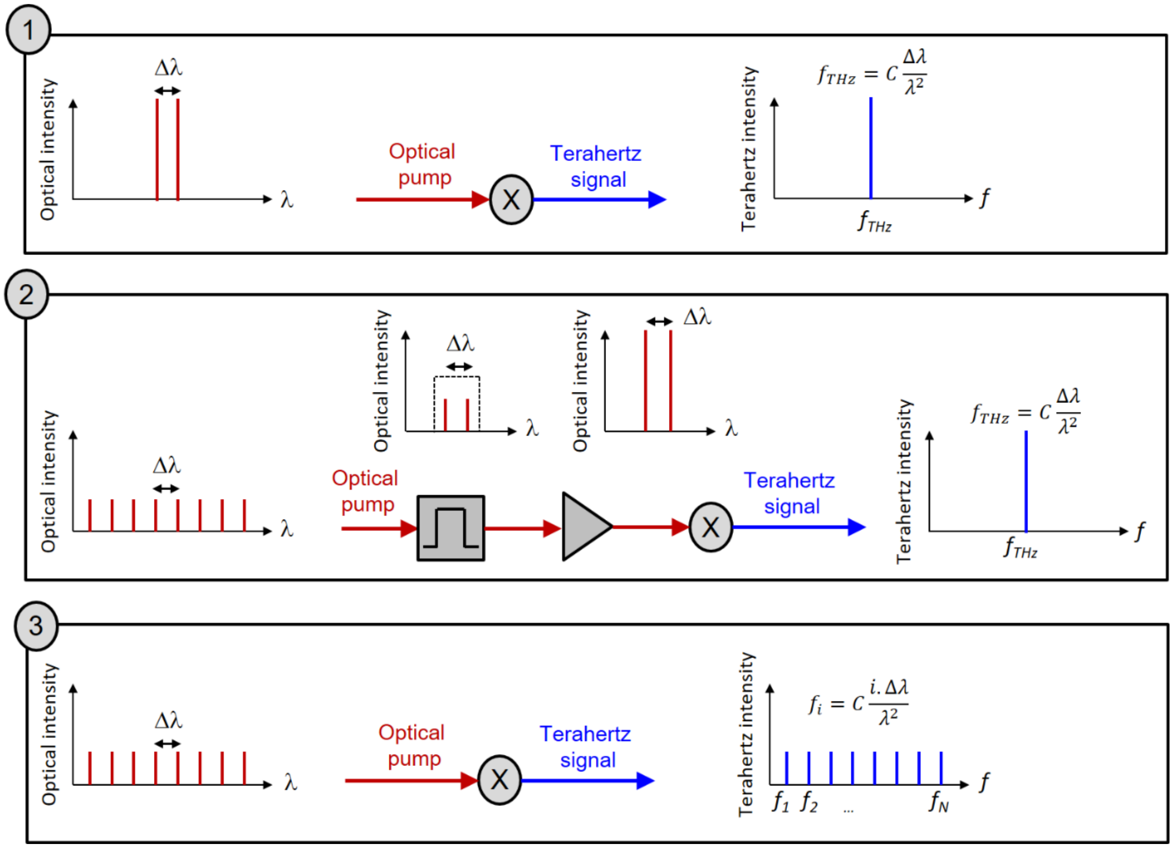

Fig. S12. Possible configurations for terahertz generation via photomixing with two-tone and multi-tone optical pump beams. (1) Two-Tone Optical Pumping: When two optical tones with a terahertz frequency difference directly pump the photomixer, a single-tone terahertz signal is generated. This configuration is ideal for producing high-power terahertz signals at a given frequency. For hyperspectral terahertz imaging or spectroscopy, the frequency difference between the optical tones must be varied, and the sample's response measured at each frequency. While this approach achieves high SNR by concentrating all optical pump power on a single frequency, it requires longer measurement times to scan across all frequencies sequentially. (2) Filtered Multi-Tone Optical Pumping: A multi-tone optical beam is used, with adjacent tones having terahertz-range frequency differences. An optical filter isolates and amplifies two tones to create a high-power two-tone beam that pumps the photomixer. This scenario functions similarly to the first but allows unused optical tones to serve additional purposes, such as optical frequency stabilization and frequency/phase locking. (3) Unfiltered Multi-Tone Optical Pumping: In this case, all optical tones are used to generate a multi-tone terahertz signal without filtering. Since the optical pump power is distributed across multiple tones, individual terahertz tones have lower power compared to scenarios (1) and (2). However, all terahertz frequencies are available simultaneously, eliminating the need for frequency tuning. This enables much faster hyperspectral imaging and spectroscopy at

the cost of reduced SNR per frequency. Each approach has advantages and trade-offs, making the choice application-dependent based on factors such as SNR, scanning speed, and spectral resolution.

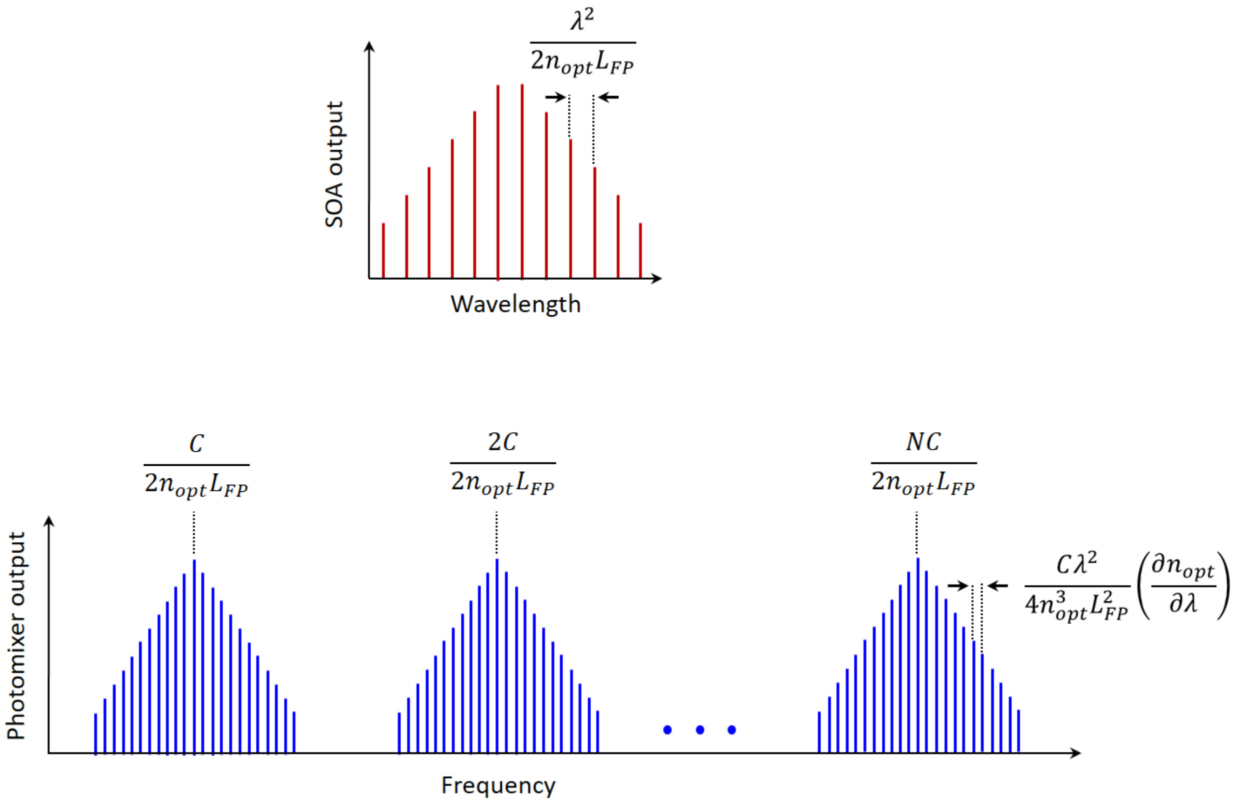

Fig. S13. The multi-mode signal generated by the photomixer (blue graph) in response to the multi-mode optical emission (red graph) from the Fabry-Perot laser. The optical tones have a wavelength spacing of $\frac{\lambda^2}{2n_{opt}L_{FP}}$, where λ is the optical wavelength, n_{opt} is the effective optical index in the laser cavity, and L_{FP} is the length of the Fabry-Perot cavity. The signal generated by the photomixing process consists of multi-mode tones centered at frequencies of $i\frac{C}{2n_{opt}L_{FP}}$, with a frequency spacing of $\frac{C\lambda^2}{4n_{opt}^3L_{FP}^2}\left(\frac{\partial n_{opt}}{\partial \lambda}\right)$, where C is the speed of light, $i = 1, 2, 3, \ldots$, and $\frac{\partial n_{opt}}{\partial \lambda}$ represents the dispersion of the optical index inside the laser cavity.

a

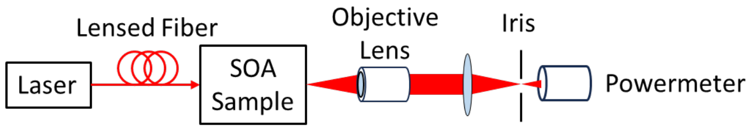

b

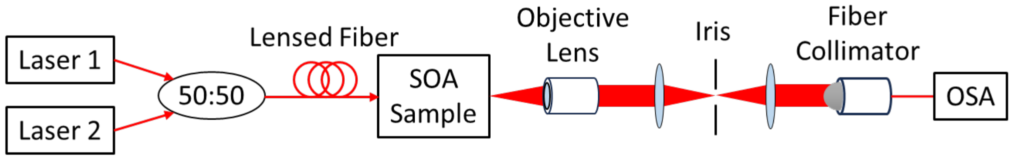

Fig. S14. The experimental setups used to characterize the output power and spectral properties of the SOA are shown in **a** and **b**, respectively.

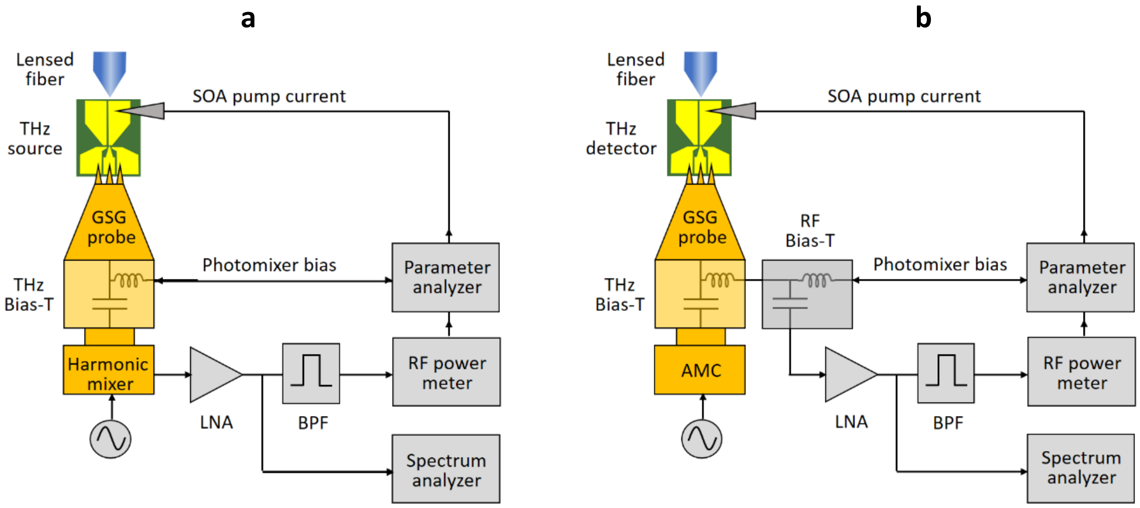

Fig. S15. The experimental setups used to characterize the terahertz sources and detectors are shown in **a** and **b**, respectively.

Reference	Type	Frequency (GHz)	P_{opt} (mW)	P_{THz} (mW)
D. Stanze, A. Deninger, A. Roggenbuck, S. Schindler, M. Schlak, B. Sartorius, Compact cw terahertz spectrometer pumped at 1.5 µm wavelength. J Infrared Milli Terahz Waves 32, 225–232 (2011).	Antenna-coupled	500	25	0.005
E. Rouvalis, C. C. Renaud, D. G. Moodie, M. J. Robertson, A. J. Seeds, Continuous wave terahertz generation from ultra-fast InP-based photodiodes. IEEE Transactions on Microwave Theory and Techniques 60, 509–517 (2012).	Antenna-coupled	306	48.1 / 38.7	0.11
E. Rouvalis, C. C. Renaud, D. G. Moodie, M. J. Robertson, A. J. Seeds, Traveling-wave Uni-Traveling Carrier Photodiodes for continuous wave THz generation. Opt. Express 18, 11105–11110 (2010).	Antenna-coupled	457 / 255	100 /40	0.148 / 0.105
J.-M. Wun, H.-Y. Liu, Y.-L. Zeng, S.-D. Yang, C.-L. Pan, C.-B. Huang, J.-W. Shi, Photonic high-power continuous wave THz-wave generation by Using Flip-Chip Packaged Uni-Traveling Carrier Photodiodes and a Femtosecond Optical Pulse Generator. J. Lightwave Technol. 34, 1387–1397 (2016).	Probed	280	130	1.04
V. Rymanov, A. Stöhr, S. Dülme, T. Tekin, Triple transit region photodiodes (TTR-PDs) providing high millimeter wave output power. Opt. Express 22, 7550–7558 (2014).	Probed	110	94	1.05
I. D. Henning, M. J. Adams, Y. Sun, D. G. Moodie, D. C. Rogers, P. J. Cannard, S. "Jeevan" Dosanjh, M. Skuse, R. J. Firth, Broadband Antenna-Integrated, Edge-Coupled Photomixers for Tuneable Terahertz Sources. IEEE J. Quantum Electron. 46, 1498–1505 (2010).	Antenna-coupled	410	50	0.15
A. Wakatsuki, T. Furuta, Y. Muramoto, T. Yoshimatsu, H. Ito, "High-power and broadband sub-terahertz wave generation using a J-band photomixer module with rectangular-waveguide output port" in 2008 33rd International Conference on Infrared, Millimeter and Terahertz Waves (IEEE, 2008), pp. 1–2.	Probed	350	91	0.54
H. Ito, T. Yoshimatsu, H. Yamamoto, T. Ishibashi, Widely Frequency Tunable Terahertz-Wave Emitter Integrating Uni-Traveling-Carrier Photodiode and Extended Bowtie Antenna. Appl. Phys. Express 6, 064101 (2013).	Antenna-coupled	200 / 500	47.6 / 47.6	0.12 / 0.017
P. Latzel, F. Pavanello, M. Billet, S. Bretin, A. Beck, M. Vanwolleghem, C. Coinon, X. Wallart, E. Peytavit, G. Ducournau, M. Zaknoune, J.-F. Lampin, Generation of mW Level in the 300-GHz Band Using Resonant-Cavity-Enhanced Unitraveling Carrier Photodiodes. IEEE Trans. THz Sci. Technol. 7, 800–807 (2017).	Probed	300	83	0.75
J.-M. Wun, C.-H. Lai, N.-W. Chen, J. E. Bowers, J.-W. Shi, Flip-Chip Bonding Packaged THz Photodiode With Broadband High-Power Performance. IEEE Photon. Technol. Lett. 26, 2462–2464 (2014).	Probed	260	162.5	0.67

J. Mangeney, A. Merigault, N. Zerounian, P. Crozat, K. Blary, J. F. Lampin, Continuous wave terahertz generation up to 2THz by photomixing on ion-irradiated In0.53Ga0.47As at 1.55µm wavelengths. Applied Physics Letters 91, 241102 (2007).	Antenna-coupled	500	40	0.00004
S.-H. Yang, M. Jarrahi, “High-power continuous-wave terahertz generation through plasmonic photomixers” in 2016 IEEE MTT-S International Microwave Symposium (IMS) (IEEE, 2016), pp. 1–4.	Antenna-coupled	150	350	0.1
H. Tanoto, J. H. Teng, Q. Y. Wu, M. Sun, Z. N. Chen, S. A. Maier, B. Wang, C. C. Chum, G. Y. Si, A. J. Danner, S. J. Chua, Greatly enhanced continuous-wave terahertz emission by nano-electrodes in a photoconductive photomixer. Nature Photon 6, 121–126 (2012).	Antenna-coupled	300 / 500	90 /90	0.09 / 0.011
N. Khiabani, Y. Huang, L. E. Garcia-Munoz, Y.-C. Shen, A. Rivera-Lavado, A Novel Sub-THz Photomixer With Nano-Trapezoidal Electrodes. IEEE Trans. THz Sci. Technol. 4, 501–508 (2014).	Antenna-coupled	200 / 400	30 /30	0.0008 / 0.0006
E. Peytavit, S. Lepilliet, F. Hindle, C. Coinon, T. Akalin, G. Ducournau, G. Mouret, J.-F. Lampin, Milliwatt-level output power in the sub-terahertz range generated by photomixing in a GaAs photoconductor. Applied Physics Letters 99, 223508 (2011).	Probed	305	162	0.35
E. Peytavit, P. Latzel, F. Pavanello, G. Ducournau, J.-F. Lampin, CW Source Based on Photomixing With Output Power Reaching 1.8 mW at 250 GHz. IEEE Electron Device Lett. 34, 1277–1279 (2013).	Probed	250	270	1.8
M. Deumer, S. Nellen, S. Lauck, S. Keyvaninia, S. Berrios, M. Kieper, M. Schell, R. B. Kohlhaas, Ultra-wideband PIN-pd THz emitter with> 5.5 THz bandwidth. Journal of Infrared, Millimeter, and Terahertz Waves 45, 831-840 (2024).	Antenna-coupled	115 / 300 / 500	39.8 mW	0.88 / 0.062 / 0.012
M. Grzeslo, S. Dülme, S. Clochiatti, T. Neerfeld, T. Haddad, P. Lu, J. Tebart, S. Makhlouf, C. Biurrun-Quel, J. L. Fernández Estévez, J. Lackmann, High saturation photocurrent THz waveguide-type MUTC-photodiodes reaching mW output power within the WR3. 4 band. Optics Express 31, 6484-6498 (2023).	Probed	240 / 280	80 mW	0.87 / 0.54

Table S1. The generated power as a function of the optical pump power for photomixer sources demonstrated in the literature over the 100-500 GHz frequency range, including those with free-space optical coupling from an external laser.
