# Supplementary material for: Terahertz generation and detection through gain-enhanced interband photomixing in quantum well structures
Source: Nat Commun. 2026 May 13;17:6247. doi: 10.1038/s41467-026-73080-6 (PMC13373183; doi:10.1038/s41467-026-73080-6)
Supplement: Supplementary file 2 — Transparent Peer Review File [file 41467_2026_73080_MOESM2_ESM.pdf]

nature portfolio

Peer Review File

Terahertz Generation and Detection through Gain-Enhanced Interband Photomixing in Quantum Well Structures

Corresponding Author: Professor Mona Jarrahi

Parts of this Peer Review File have been redacted as indicated to remove third-party material.

This file contains all reviewer reports in order by version, followed by all author rebuttals in order by version.

Attachments originally included by the reviewers as part of their assessment can be found at the end of this file.

Version 0:

Reviewer comments:

Reviewer #1

(Remarks to the Author)
The authors present an optoelectronic platform for generating and detecting terahertz radiation. These integrated THz platforms are intriguing from a scientific standpoint and have great potential for addressing real-world applications. Unfortunately, I am not convinced that the results support the claims or align with the journal's scope in terms of completeness, impact, or performance compared to existing technology. Details are provided below. In summary, I see a number of shortcomings. First, the comparison of the device's performance to that described in the literature is unfair. Second, central components of the platform have not been presented, nor has it been made clear how they could be realized. Third, no more than two functionalities have been demonstrated on a single chip: SOA and the THz emitter. Consequently, there is insufficient demonstration of integration into working devices to substantiate the platform claim. I cannot recommend publishing this work as it is, I recommend major revisions.

- The abstract claims: "...to realize all terahertz photonic system components onto a single chip for the first time." In the introduction, it states: "Previous work on waveguide-coupled terahertz sources and detectors required external lasers to function." However, no measurements have been taken with at least one laser and a photodiode on a single chip. Therefore, integration of the components into a common platform has not been demonstrated; it has only been shown in parts. This is critical because of the potential for high crosstalk, or interaction through back reflections, between components that could drastically reduce performance.
- The claimed platform lacks passive waveguides and couplers. Low-loss waveguides and couplers are highly relevant for the claimed functionality, especially for more complex designs, as shown in Fig. 4(d). The layer stack shown suggests that the quantum-dot layer is also the waveguiding layer. In that case, the passive components would also have to include the quantum-dot layer, which would result in extreme losses. Based on the information provided, I cannot identify a method to incorporate passive components into the device. Without such components, I cannot support the claim of a platform.
- The demonstrated laser relies on end-facet coating. As a result, it cannot be integrated into this configuration or claimed as a component of the platform, although DBR or DFB structuring seems feasible.
- Figure 4d is misleading because the final vision would not be possible with the components shown. No passive waveguides or couplers were demonstrated. Furthermore, phase modulation has been demonstrated with a couple of pi at the laser frequency. However, depicted beam steering at THz frequencies that uses a single multicolor laser source would require phase shift ranges of the order of the THz wavelength, which is at least two orders of magnitude higher, because all laser lines pass through the same phase shifter and experience a similar phase shift. This is certainly not possible with this integrated approach.
- The phase and amplitude modulator appear to be the same device with the same driving signals. Based on the information provided, it can be concluded that phase and amplitude cannot be modulated independently. This drastically limits the device's usability and impact.
The authors claim that their platform has advantages over other photonic integration platforms, such as reduced complexity and improved THz emitter and receiver performance. However, the figures of merit used here are uncommon and/or incomparable to those in other publications. Some of these are discussed in more detail below.
Emitter/PD:

- The authors claim that the THz emitter achieves a higher optical-to-THz efficiency than state-of-the-art technology. However, they include the SOA in this comparison and compare it with literature that did not use extra optical signal amplification. Therefore, this is not a fair comparison. To make a fair comparison, the efficiency of the photodetector (PD) without the SOA must be used. Furthermore, the comparison omits some current state-of-the-art publications, such as: [1], [2].
- Responsivity values and IV curves are not provided for the PD.

Detector:

- Although it is claimed that the detector surpasses state-of-the-art detectors, the provided comparison is invalid for multiple reasons:

o The comparison was only made to direct detectors. The presented receiver is a multiplicative mixer that uses an optical local oscillator (LO), but it is compared to direct detectors, which scale differently with respect to bandwidth. A more accurate comparison would include mixers with an electrical or optical local oscillator, such as Schottky diode-based subharmonic mixers or photoconductive mixers. Such mixers, as described in the literature, provide far superior sensitivity than that demonstrated here. [3], [4].

o Unsuitable Measurement Scheme: The input-referred noise (also known as sensitivity or NEP) of a mixer is not a function of integration time or bandwidth. However, due to the radiometer measurement scheme that was applied (bandpass filter and power detector), the value appears to be much lower than it actually is. It also scales with the square root of the integration time. This makes the comparison with other detectors invalid and is strongly misleading. For example: Using the bandpass filter (BW: 140 MHz) and an integration time of 0.1 s, the input-referred noise of the mixer itself is 3,700 times higher than indicated by Fig. 2(h), contradicting the claimed sensitivity.

o Missing: Comparison with the authors' own previous work. The authors did not compare their results to those of their previous publication, in which they used a very similar measurement scheme and detector principle. This would be a fair comparison. However, the sensitivity value provided in the previous publication is over 100,000 times better than what was demonstrated here. This disproves the claim of state-of-the-art performance.

o Discrepancy Between Figures 2h and 3b: In Figure 3b, the noise equivalent power is plotted in pW/√Hz for comparison with direct detectors. How is the unit fW/√Hz in Fig. 2(h) converted to pW/√Hz in Fig. 3(b)?

- No IV curves are provided as a function of optical power that would show the conductivity modulation used in the mixing process.

Modulators:

Common figures of merit, such as the modulators' bandwidth and insertion loss, should be provided.

[1] M. Deumer et al., "Ultra-Wideband PIN-PD THz Emitter with > 5.5 THz Bandwidth," J Infrared Milli Terahz Waves, vol. 45, no. 9, pp. 831–840, Oct. 2024, doi: 10.1007/s10762-024-01001-z.
[2] M. Grzeslo et al., "High saturation photocurrent THz waveguide-type MUTC-photodiodes reaching mW output power within the WR3.4 band," Opt. Express, OE, vol. 31, no. 4, pp. 6484–6498, Feb. 2023, doi: 10.1364/OE.475987.
[3] "Virginia Diodes, Inc - Mixers." Accessed: Jul. 16, 2025. [Online]. Available: https://www.vadiodes.com/en/products/mixers-shm-ehm-and-fm
[4] M. Deumer et al., "Continuous wave THz receivers with rhodium-doped InGaAs enabling 132 dB dynamic range," Opt. Express, OE, vol. 32, no. 17, pp. 29855–29867, Aug. 2024, doi: 10.1364/OE.532465.
[5] K. Rohlfs and T. L. Wilson, Tools of Radio Astronomy. in Astronomy and Astrophysics Library. Berlin, Heidelberg: Springer, 1996. doi: 10.1007/978-3-662-03266-4.
[6] N. Wang, S. Cakmakyapan, Y. J. Lin, H. Javadi, and M. Jarrahi, "Room-temperature heterodyne terahertz detection with quantum-level sensitivity," Nature Astronomy 2019 3:11, vol. 3, no. 11, pp. 977–982, Jul. 2019, doi: 10.1038/s41550-019-0828-6.

Reviewer #2

(Remarks to the Author)
In the present manuscript the authors demonstrate an integrated optoelectronic terahertz platform that integrates THz source, detector, modulator and other passive components on one chip, all based on PIN quantum well photodiodes.

While the presented platform is with no doubt very powerful and elegant, I have several questions and concerns which I will outline in the following.

Page 2: The sentence on "biological tissues" may deserve a remark about the problem of water absorption, ubiquitous in biological systems.

Page 4: About this sentence: "This limitation arises because previous studies have been limited to photomixing schemes utilizing intersubband photon absorption in QW PIN photodiodes 23,24":
Refs. 23 and 24 describe two different schemes, but neither of them is based on a PIN photodiode (but rather on unipolar structures).

Page 5:
Usually photomixers rely on the ultrafast carrier relaxation/recombination across the band gap (like in low-T grown GaAs). Here the transit time apparently takes this role. It would be good to make a remark on this difference compared to standard photomixers.

Page 6 top: why are the saturation velocities for electrons and holes nearly the same, considering the vastly different

effective masses ? Is there a simple argument for this ?

Page 6: what does this half-sentence mean:
“Leveraging the ultrafast carrier dynamics that support interband photon absorption“ ?
What does the absorption have to do with the ultrafast carrier dynamics ?

Page 7:
The SOAs are introduced without any particular reasoning. Not being an expert in integrated photonics, I was asking myself whether one always needs an SOA , or if just two lasers could be sufficient. Are DFB lasers never powerful enough (see also next question) ? It would be good to give some more explanations on this issue.

Page 8:
In this connection I see another problem: despite the promise of a completely integrated platform, the authors finally use two external lasers with slightly different wavelengths as pump sources. The claim that they also could be integrated sounds a bit too casual (“Two commercially available lasers are used to characterize both the SOA and the monolithically integrated terahertz source/detector. The two lasers can be replaced by two wavelength tunable lasers fabricated on the same QW PIN photodiode substrate 31.”)
Indeed Ref. 31 presents such a laser pair (power 15 mW !) – but would this really be so easy to be implemented in the present platform ? If yes, why isn’t this demonstrated then ? If no, an honest discussion should be given.

Fig. 2a: The eight used colors are somehow not different enough; it is difficult to distinguish and assigning the different layers.

The data in Fig. 3a and 3b are very impressive, showing that the present QW photomixer outperforms most (or all) devices in the literature, both for THz sources and detectors. Here the question arises, why ? Do the authors have an intuitive or even substantiated explanation why their device is better than anything else produced during the past two decades ? This may actually be hard to believe and one may wonder about calibrations etc.

And a related question: can one and the same QW PIN structure really be a “Swiss army knife”, i.e. be optimally suited for all different purposes: source, detector, modulator, SOA, filter, demultiplexer ? I guess one has to make comprises concerning the performance for the different functionalities ?

Reviewer #3

(Remarks to the Author)
In this paper, the authors propose a concept for integrating terahertz photonic system components onto a single chip, using a quantum well structure as a key element. Among the various aspects, the most original point is the generation and detection of terahertz waves via photomixing using a QW PIN photodiode. Both experimental and theoretical evaluations have been conducted in detail, and the authors claim that they have achieved a power conversion efficiency exceeding that of conventional technologies. The device fabrication and evaluation methods have been carefully considered and are described thoroughly, making them credible. This represents an important technology that contributes to the miniaturization and cost reduction of terahertz technology.

One major concern, however, lies in the method of comparison with previous literature shown in Fig. 3. The power output values reported in previous works are basically the power of electromagnetic waves emitted into free space through an antenna. In contrast, the power evaluated in this paper is calculated from the current before it is converted into electromagnetic waves, meaning that the antenna conversion efficiency (loss) is not included. This does not constitute a fair comparison, and therefore does not adequately support the authors’ claim of achieving higher conversion efficiency than conventional technologies. The same issue applies to the detector operation as well. This point needs to be corrected before considering publication in Nature Communications.

Additional comments are provided below:

•Regarding previous studies on detectors, the following paper is not included. This device is already commercially available and represents an important point of comparison:
Ito, H., and Ishibashi, T.: “InP/InGaAs Fermi-level managed barrier diode for broadband and low-noise terahertz-wave detection”, Jpn. J. Appl. Phys., 2017, 56, (1), pp. 014101-1–014101-7

•The evaluation method for the phase modulator operation shown in Fig. 4c should be described in more detail.

•Since the quantum-confined Stark effect changes both the absorption coefficient and the refractive index, using it as a phase modulator inevitably causes simultaneous modulation of the intensity. The authors should comment on whether this could pose a problem in future applications.

Version 1:

Reviewer comments:

Reviewer #1

(Remarks to the Author)
Thanks to the authors for revising the manuscript. The new title accurately reflects the content of the paper. I agree with the authors that demonstrating emitters and receivers based on the same QW PIN stack is an interesting work.

Many of the previous concerns were addressed, but important issues in the methods remain. These issues invalidate and mislead some of the comparisons and claims. This includes the publication's central claims about the sensitivity of the receiver.
Below, I provide a brief summary of each critical aspect and have attached a more thorough explanation of each one.

Receiver sensitivity
The authors claim that the receiver has a '…sensitivity over the state-of-the-art.' (l. 14). However, the results in this work were obtained using the evaluation scheme 'spectral lock-in' which differs significantly from the evaluation methods used in the other cited publications. The different evaluation method produces different results, rendering a fair comparison impossible. There are multiple indications in the presented data that support this claim. The most prominent indication is that the sensitivity of a receiver is a fixed value and therefore should not scale with integration time. However, the data in Fig. S6f and Fig. 2h show scaling with integration time, indicating that the authors used a radiometer scheme. That said, the 10 dB/decade increase in sensitivity with integration time (Fig. S6a, f, and 2h) raises concerns about signal evaluation. Normal lock-in produces constant sensitivity, whereas radiometer noise scales at 5 dB/decade.
In contrast, the other publications (ref. 35 and ref. 36) use a standard lock-in scheme and demonstrate sensitivity independent of integration time, as expected.
In summary, the authors obtain their sensitivity values differently than state-of-the-art publications. Consequently, their sensitivity appears to be orders of magnitude higher and thus beyond state of the art , which is an unsubstantiated claim. Furthermore, their previous publications with comparable methods but far better results are missing.

Terahertz emitter
The discussion of the SOA integration is noted, and I agree with the authors that the 'gain enhancement' is an interesting feature. However, this is also possible based on already available technologies like photonic integration platforms that provide building blocks for PD and SOA. Consequently other platforms already demonstrated the so-called 'gain-enhanced ' terahertz generation by integrating an SOA with a PD [4], [5].
The claimed record efficiency value is largely defined by SOA gain. A fair comparison to the state of the art would require include the efficiency of the PD itself.
In conclusion, combining SOAs and photodiodes for terahertz generation is possible on other platforms and has been demonstrated. Furthermore, the claim of '…significant improvements in power efficiency … over state of the art' (line 14) cannot be made, as other publications on gain-enhanced THz emitters did not consider this figure of merit.

Introduction
As a motivation for this work, the authors claim that '…Previous work on waveguide-coupled terahertz sources and detectors has lacked compatibility with photonic integrated circuits (PICs)…'(l. 56-58) while both, emitters and receivers, are already shown to be compatible: Ref. 21 [1] of the manuscript demonstrates integration of the source. Additionally, there are publications showing that photodiodes can also be used as detectors[2]. Furthermore, waveguide-integrated photoconductors are demonstrated, which are suitable for photonic integration [3].
Previously Published Plots and Graphs
Many of the key graphs were included in publications made before or during the review process of this manuscript (see attachment for an overview). While a comprehensive publication of all the results in one place is valuable, it could potentially conflict with some journal guidelines. I will leave it to the editor to evaluate this.

Conclusion
I cannot recommend this manuscript for publication. My primary concern is the method used to evaluate receiver sensitivity, which seems questionable and differs fundamentally from other recent literature. This results in highly overestimated sensitivity values and a strongly misleading presentation. Furthermore, this method cannot be used to claim that sensitivity exceeds that of state-of-the-art receivers.

Attachment:

The following explanations are intended to provide background information to help you understand the nature of my concerns.

1. Lock-In Technique vs. Radiometer

The authors appear to use a radiometer measurement scheme to determine the sensitivity value of their receiver, whereas the references use a lock-in technique. The following will explain the main differences between the two approaches and why the results differ.
The radiometer scheme is common in astronomy and weather/atmosphere measurements because it surpasses actual receiver sensitivity by orders of magnitude. This is done by comparing noise power and noise plus signal power.
The process involves modulating the source and then comparing the off and on states. Essentially, it averages the noise power based on the off state in a fixed bandwidth and subtracts this value from the on-state signal. This subtraction scales with integration time because the standard deviation of the mean values scales with the square root of N, or in this case, the square root of τ. This explains why input-referred noise (sensitivity) scales with integration time. However, this is not an inherent noise value; it is the standard deviation of the noise. Consequently, using those sensitivity values without first calculating the original noise using the radiometer equation renders them completely incomparable to regular measurements. With the 1.5 MHz bandwidth and 64 ms integration time used here, the smallest detectable power appears ~300 lower than the actual receiver sensitivity.
Unlike a radiometer, the standard lock-in scheme is essentially a bandpass filter with I/Q demodulation. It does not involve power averaging, and the integration time is inversely proportional to the bandwidth. Therefore, integrating for a longer time reduces the bandwidth, allowing one to distinguish a narrowband signal from the noise. With ten times the integration time, the bandwidth and integrated noise power are both reduced by a factor of ten. However, the input-referred noise power spectral density (sensitivity) in W/Hz remains constant as expected.

2. 'Spectral Lock-in' Scheme and Receiver sensitivity
In the rebuttal, the authors write that refs. 7 and 8 also use a lock-in technique. However, they overlook the fact that their own measurement scheme is still quite different. They refer to their measurement scheme as "spectral lock-in" and claim a 10 dB/decade improvement in noise and signal-to-noise ratio (SNR). However, input-referred noise reduction with integration time is only possible with radiometers, which would result in a 5 dB/decade reduction, as seen in the original manuscript. Furthermore, their scheme includes a fixed power integration bandwidth of 1.5 MHz and a modulated signal source, similar to a radiometer setup. In this configuration, achieving a 10 dB/decade reduction in noise is not plausible and suggests a significant flaw in the signal processing. Therefore, the measurement results are highly questionable. The authors neither explain the measurement scheme in detail nor cite the publication in which they use the term "spectral lock-in." [6].
In order to fully understand this measurement scheme, the authors must provide:
(a) A detailed block diagram and description explaining the entire signal chain and processing.
(b) Measurements with different source input powers to verify the linearity of the measurement scheme.
c) Show the phase information of the spectra in Fig. S6c.

Nevertheless, the provided results indicate that the measurement scheme cannot be used to compare the receiver to literature values obtained by different methods, even without detailed knowledge of the evaluation process.
The publications referenced in ref. 35 and ref. 36 use a standard lock-in scheme, and, as expected, sensitivity is independent of integration time. However, the authors do not properly explain why the setup in reference 7 is incomparable to theirs. Furthermore, in references [6] and [8], the authors appear to use the same "spectral lock-in" scheme as in this manuscript while reporting sensitivity orders of magnitude better (2.5 K noise temperature in reference [8] is equivalent to 3.45×10^(−8) fW/Hz).
There are further indications that the scheme used in the manuscript produces inconsistent results:
a) Conversion loss and input-referred noise vary between manuscripts and methods: As previously mentioned, the relationship between sensitivity and conversion loss should remain consistent. However, this relationship changes from ~1 fW/Hz @ 45 dB in the first manuscript to ~0.02 fW/Hz @ 45 dB in the revised manuscript, which is a mismatch of more than one order of magnitude. Neither value matches the theory, and the authors do not explain why the values and their relation changed so much. The only reasonable conclusion is that both measurement schemes largely distort the results. If these measurement schemes provided reliable sensitivity values, they would match.
b) Unclear choice of integration times and bandwidths: As mentioned above, the measurement schemes produce very different and implausible results. It is unclear why the integration time was 25 ms in the initial manuscript and 64 ms in the revised one. This is confusing, given that the inset in Fig. 2h indicates that they measured both in a region from 0.1 ms to 100 ms. Furthermore, if the input-referenced noise is not constant, but rather scales by 10 dB/decade or 5 dB/decade, how can one select a plausible integration time and bandwidth for the comparison? If both manuscripts used the same integration time, the discrepancy explained in (b) above would be more obvious.
c) Integration Time and Sensitivity: This is just for completeness, since it was mentioned earlier. The sensitivity of a receiver does not increase with longer integration times.
In conclusion, a common method for measuring receiver sensitivity must be used to allow for a fair comparison to the state of the art. The following procedure can be used: First, measure the receiver output noise with an ADC. Then, calculate the power spectral density without subtracting anything. Finally, refer this spectral power density to the input with the conversion loss. This can be done with the measured data, so it should be possible with minimal effort.

3. Previous publications
See image attached.

References
[1] G. Carpintero et al., "Microwave Photonic Integrated Circuits for Millimeter-Wave Wireless Communications," Journal of Lightwave Technology, vol. 32, no. 20, pp. 3495–3501, Oct. 2014, doi: 10.1109/JLT.2014.2321573.
[2] C. C. Renaud, M. Natrella, C. Graham, J. Seddon, F. Van Dijk, and A. J. Seeds, "Antenna Integrated THz Uni-Traveling Carrier Photodiodes," IEEE Journal of Selected Topics in Quantum Electronics, vol. 24, no. 2, pp. 1–11, Mar. 2018, doi: 10.1109/JSTQE.2017.2725444.

[3] M. Deumer et al., “Waveguide-integrated photoconductive THz receivers,” in 2022 47th International Conference on Infrared, Millimeter and Terahertz Waves (IRMMW-THz), Aug. 2022, pp. 1–2. doi: 10.1109/IRMMW-THz50927.2022.9895580.
[4] G. Carpintero et al., “Photonic-Enabled Terahertz Phase Arrays Using Dielectric Rod Waveguides for 6G Wireless Communications,” in 2024 IEEE/MTT-S International Microwave Symposium - IMS 2024, June 2024, pp. 74–77. doi: 10.1109/IMS40175.2024.10600234.
[5] G. Carpintero, R. C. Guzmán, C. Gordón, G. Kervella, M. Chitoui, and F. Van Dijk, “Photonic Integrated Circuits for Radio-Frequency Signal Generation,” Journal of Lightwave Technology, vol. 34, no. 2, pp. 508–515, Jan. 2016, doi: 10.1109/JLT.2015.2511040.
[6] S.-A. Tsao, J. Hwang, and M. Jarrahi, “Lock-in detection of terahertz spectra captured by plasmonic heterodyne terahertz receivers,” in Terahertz, RF, Millimeter, and Submillimeter-Wave Technology and Applications XVIII, SPIE, Mar. 2025, p. PC1336508. doi: 10.1117/12.3046514.
[7] N. Wang, S. Cakmakyapan, Y. J. Lin, H. Javadi, and M. Jarrahi, “Room-temperature heterodyne terahertz detection with quantum-level sensitivity,” Nature Astronomy 2019 3:11, vol. 3, no. 11, pp. 977–982, July 2019, doi: 10.1038/s41550-019-0828-6.
[8] J. Hwang, S.-A. Tsao, and M. Jarrahi, “Ultra-low-noise, room-temperature terahertz receiver based on plasmonic photomixing,” in Terahertz, RF, Millimeter, and Submillimeter-Wave Technology and Applications XVIII, SPIE, Mar. 2025, p. PC1336502. doi: 10.1117/12.3047609.
[9] Y. Zhao, S.-E.- Zumrat, and M. Jarrahi, “Monolithically Integrated Terahertz Optoelectronics Based on Quantum Well Structures,” in 2024 49th International Conference on Infrared, Millimeter, and Terahertz Waves (IRMMW-THz), Sept. 2024, pp. 1–2. doi: 10.1109/IRMMW-THz60956.2024.10697774.
[10] Y. Zhao and M. Jarrahi, “Monolithically integrated terahertz optoelectronics based on quantum well structures,” in Terahertz Emitters, Receivers, and Applications XV, SPIE, Oct. 2024, p. PC1314104. doi: 10.1117/12.3028428.
[11] Y. Zhao, S.-E.- Zumrat, and M. Jarrahi, “Monolithically Integrated Optoelectronic Terahertz Sources and Detectors on Quantum Well PIN Substrates,” in 2025 IEEE/MTT-S International Microwave Symposium - IMS 2025, June 2025, pp. 126–129. doi: 10.1109/IMS40360.2025.11103976.

Reviewer #2

(Remarks to the Author)
The authors answered most of my questions, clarified many things and put their achievements in more realistic and honest perspective. One thing I am still not completely satisfied with is the performance comparison: they relate the performance of their device to the unamplified input laser power, with the argument that the power after SOA amplification is not known and cannot be measured. But at least it must be possible to give an estimation (not to speak about fabricating a SOA test device and measure the amplified output). Is it a factor of 10 ? Or more likely 100 ? This uncertainty should also be stated where the performance is discussed (near Fig 2 and/or 3.)

Reviewer #3

(Remarks to the Author)
The revisions have clarified the authors’ claims, and the title has been updated accordingly. In my opinion, the work is on a rather specific terahertz technology and may not attract broad interest. Therefore, I believe it would be more suitable for a more specialized journal rather than Nature Communications. My additional comments are as follows:

• While I can accept the inclusion of performance improvements achieved with the SOA, this result by itself does not appear to carry substantial scientific impact.

• The constraints imposed by the Kramers–Kronig relations apply when considering the full frequency response. In practice, however, devices are typically operated in a very narrow frequency window—for example, in a transparency region—where such effects are negligible and the device can essentially function as a pure phase modulator. In the present case, the absorption is significant, which raises concerns that the situation is quite different from that of a general modulator.

Version 2:

Reviewer comments:

Reviewer #1

(Remarks to the Author)
Thank you to the authors for revising the manuscript once again, and for providing such a detailed response. The SOA gain estimation and the additional data points in Fig. S7 now enable a fair comparison and eliminate the question of the SOA's influence. The emitter's efficiency appears to be very good, which is a remarkable result given the use of a multi-purpose epitaxial stack.
In contrast, the method used to assess receiver performance remains essentially unchanged, meaning that the results cannot be compared to those in other publications. This is clearly evident in the sensitivity of the receiver, as shown in Fig. 2h and Fig. S6f. According to the fundamental principle of receiver input-referred power spectral density, it must not scale

with integration time.
To support this, I provide an overview of the reasons why the sensitivity values given in the manuscript are invalid:
a) Fig. 2h and S6f clearly show scaling of input-referenced noise in W/Hz with integration time. However, this is fundamentally impossible; the power spectral density must not change with integration time.
b) Relation between input-referred noise and conversion loss: The conversion loss of a receiver is fundamentally linked to its sensitivity and input-referred noise. Therefore, the fundamental limit of a receiver's sensitivity/input-referred noise can be calculated [1]. With -174 dBm/Hz thermal noise at room temperature (where the noise power is equal to the temperature multiplied by Boltzmann's constant) and 43 dB conversion loss (see Fig. 2h), the sensitivity of the receiver is, at best, -131 dBm/Hz (0.08 fW/Hz). However, Fig. 2h shows that the authors claim to have reached 0.02 fW/Hz, which would be approximately 6 dB better than the fundamental limit. In the inset, they even claim to have increased this by more than an order of magnitude with integration time.
c) Phase information: In Fig. S6c, the authors have added an inset showing the phase information over integration time, as requested. The presented phase data supports the assumption that they are using only the magnitude in their lock-in scheme, as explained below. If they were acquiring the actual source signal phase, it would be random due to the heterodyne measurement principle; however, here it is constant/converging. This plot most likely represents the phase of the 15 kHz modulation rather than the source signal, indicating that the scheme used is related to a radiometer.
To resolve this issue, the authors must use input-referenced noise power density, based on unprocessed output power spectral density divided by conversion loss, for comparison purposes. This value was included in the rebuttal as 20 fW/Hz at ~140 GHz.
Below, I have provided comments on the author’s response.
1) “Benchmarking and comparison with other detectors in the literature”
a) The authors argue that a comparison is not straightforward, stating that “Benchmarking is particularly tricky for terahertz detectors, since there are several different types of detectors…”. I disagree. While there are certainly different types of detector with different bandwidths and coupling schemes, the sensitivity in W/Hz of mixer-based detectors with a local oscillator is a well-established figure of merit that enables receivers to be compared regardless of bandwidth or integration time. The same applies to noise temperature, which is a different representation of sensitivity and an equally universal figure of merit.
b) The authors state: “Based on the reviewer’s comment, we revised our manuscript and modified our comparison with the detectors suggested by the reviewer (exactly the same reference that the reviewer suggested). Therefore, it is very unfair that the reviewer is now questioning the fairness of this comparison that he himself suggested“. I disagree. In my initial comment, I stated that there are multiple reasons why this comparison is invalid, including the measurement scheme. Resolving just one of these issues does not change the fact that the comparison is invalid.
c) The authors wrote: ”We indeed explained why the sensitivity values changed from our original manuscript to the revised manuscript. Quoting from our response to the reviewer in the previous round:…”. The authors explained the reduction in noise power. However, it remains unclear how the noise power scales with integration time when the signal power remains constant at a fixed resolution bandwidth of 1.5 MHz. Furthermore, as the noise is not reduced by narrowing the bandwidth as it would be with a regular lock-in, the reason for the difference in noise power reduction between the two schemes remains unclear.
d) In the rebuttal, the authors list the differences between the measurement schemes in Ref. 35 and their own manuscript. In this comparison, the setup used in the manuscript is referred to as a 'Photomixer-based detector operating in an incoherent direct detection scheme'. This implies that the integrated noise power would scale by 5 dB per decade. However, in the provided data, it scales with 10 dB per decade (integrated noise power in fW, with a constant power spectral density). Furthermore, the scheme would only be considered a direct detection scheme if the authors used the magnitude of the FFT in their lock-in scheme instead of the complex value, as mentioned below. This would also mean that the setup would be equivalent to a radiometer, meaning that the presented values would be invalid for comparison purposes (in general, a direct detector with a chopped emitter and lock-in amplification would be equivalent to a radiometer). In contrast, in Ref. 35, the emitter is not modulated and the integration is equivalent to a second filter stage. Thus, the noise is simply reduced by narrowing the bandwidth, and the sensitivity in fW/Hz can easily be calculated. More importantly, it stays constant over integration time.
e) The authors state that their results are not comparable with those of their previous publication [2] because different measurement schemes were employed. However, noise temperature and sensitive/input-referred noise describe the same physical property and are directly convertible [1]. This raises the question of why the authors claim that these values depend on the measurement scheme. If this were true, any comparison with values from other sources would be invalid.

2) Spectral lock-in
a) In lock-in measurements, the signal-to-noise ratio (SNR) scales with integration time. This is because the integration acts as a band-pass filter that becomes narrower with increasing integration time. Consequently, the proportion of the spectrally constant noise power spectral density in W/Hz that falls within this filter band decreases as the integration time increases. However, this also means that, for heterodyne measurements on a broadband source (i.e. a source with a bandwidth greater than that of the lock-in filter), the narrowing of the lock-in filter bandpass with integration time will not increase the SNR. In this case, the sensitivity of the detector remains the fundamental limit of the minimum detectable power per bandwidth, which is independent of integration time. However, the authors modulate the source power and run the lock-in on this modulation. This makes it possible to measure power levels much lower than the receiver's sensitivity. This method is common in many fields since it enables very weak signals to be measured, almost independently of the receiver's sensitivity. However, this scheme is not suited to determining the sensitivity of a detector itself, since it measures the sensitivity of the entire setup, including modulation of the source. Therefore, the extracted power levels do not represent the sensitivity in W/Hz as a fundamental property of the detector. Consequently, these values cannot be compared to sensitivity values provided in the literature. A detailed block diagram would certainly help with understanding the measurement scheme. My understanding of

the scheme is as follows: Assuming the signal source produces a signal at 100.1 GHz: This source is switched on and off at a frequency of 15 kHz, as mentioned in the manuscript. The receiver is then driven by an optical beatnote at ~100 GHz, which is down-converted to an intermediate frequency (IF) of ~100 MHz. In the sampled data, we therefore see alternating short noise periods and periods with an IF signal at ~100 MHz. A short-time FFT with 1.5 MHz bins is then applied, which is equivalent to filtering with many 1.5 MHz bandpass filters. For simplicity, only the FFT bin/bandpass around 100 MHz is considered. In the source-on state, the complex amplitude within this FFT bin will contain the IF (source) signal. However, due to the free-running lasers, the phase will be uncorrelated, random and will vary rapidly over time. In the source-off state, there will only be noise. When a lock-in amplifier is applied to this complex-valued signal, both the noise and the source signal will integrate to zero due to the uncorrelated phase. Thus, there will be no output signal to monitor. Nevertheless, the authors report signal and noise reduction over time. This can only be achieved by applying the lock-in scheme to the IF magnitude rather than the complex signal. In this case, the off-state integrates to the average noise magnitude and the on-state integrates to the average source signal magnitude, i.e. the noise power and signal power. The result of the lock-in is therefore the change in power between the noise and the signal, just like a radiometer. Because the variance of the average noise power scales with the square root of the number of samples, the noise reduction is 5 dB per decade. This is a digital implementation of a 'multichannel filter spectrometer' [3].
In conclusion, this scheme either measures no signal at all when a complex signal is used, or it measures power as a radiometer would when magnitude is considered. However, it should never reduce noise power by 10 dB per decade.
b) I requested a comparison with the author's previous publication (Ref.). 48. The author replied that the results are not directly comparable and that the differences could be up to four orders of magnitude. I see two issues here: i) The result from Ref. 48 are still many orders of magnitude better and should be used as the benchmark in your comparison. ii) Why not conduct the same measurement under similar conditions to establish at least one direct, unquestionable point of comparison?
c) The authors claim that constant sensitivity over integration time only applies to coherent homodyne measurements and is not relevant to their receiver. I disagree. For a heterodyne detector, sensitivity is independent of integration time, just as it is for any electronic frequency extender used with a spectrum analyser. The situation is different for direct detectors without a local oscillator, such as some Schottky detectors or Golay cells. These effectively integrate the power of all frequencies and cannot provide spectral (frequency and phase) information.
d) I requested the input-referred noise without any temporal integration. The authors state that this is included in Fig. S6b. However, it seems that this graph already incorporates some form of integration or time averaging, and possibly even the lock-in stage. This would affect the power spectral density values. I was also unable to find information on whether 64 µs is used as the window length for the short-time FFT. A frequency resolution of 1.5 MHz would correspond to a window of just ~0.64 µs. In conclusion, it seems that the 64 µs refer to the lock-in period, indicating that lock-in demodulation of the magnitude is applied. Therefore, this is not the actual power spectral density that I requested.
3) Terahertz Emitter:
a) In their rebuttal, the authors wrote that "…Ref. 21 ([4]) and [5] use uni-traveling-carrier photodiodes that are not compatible with standard photonic integrated circuits (PICs) offered by foundries. Ref. 21 used a fabrication process that required three epitaxial growth steps. Quoting from the authors of Ref. 21 ([4]) and [6]: "The main drawbacks of this approach are that it requires a dedicated fabrication process flow."" However, in Ref. 21 [4], UTC-PDs are implemented alongside SOAs, lasers, waveguides and modulators on a single chip, while in Ref. [7], PIN-PDs are implemented with SOAs and waveguides. In Ref. 21 [4], PDs based on UTC-PDs are described, and it is mentioned that „This process flow uses fabrication steps that are compatible with the integration of MQW active sections and the realization of shallow ridge laser and SOA waveguides." [8]. Furthermore, typical foundry platforms for photonic integration commonly use multiple epitaxial growth steps. This means that UTC-PDs are just as compatible with photonic integration as PIN diodes. There are also further examples of photonic integrated UTC-PDs, such as [9].
b) The authors wrote, "Beyond scalability issues, these integrated terahertz optoelectronics schemes have been limited to generating signals up to ~100 GHz without demonstrating high-frequency signal detection." However, this is not accurate, as [7] demonstrated an emitter with SOAs and four PIN-PDs that emitted at 210 GHz.
c) The authors replied that "…monolithic integration with other active/passive optoelectronic components on the same chip is the unique advantage of our devices." As previously mentioned, there are multiple publications demonstrating the integration of multiple active and passive optoelectronic components on a single chip [4, 7, 9]. There are also publications demonstrating photonic integration-compatible emitters, receivers, or both [5, 8, 9, 10, 11].

[1] D. M. Pozar, Microwave Engineering, 4th ed. Hoboken, NJ: John Wiley & Sons, Inc., 2012.
[2] N. Wang, S. Cakmakyapan, Y. J. Lin, H. Javadi, and M. Jarrahi, "Room-temperature heterodyne terahertz detection with quantum-level sensitivity," Nature Astronomy 2019 3:11, vol. 3, no. 11, pp. 977–982, July 2019, doi: 10.1038/s41550-019-0828-6.
[3] K. Rohlfs and T. L. Wilson, Tools of Radio Astronomy. in Astronomy and Astrophysics Library. Berlin, Heidelberg: Springer, 1996. doi: 10.1007/978-3-662-03266-4.
[4] G. Carpintero et al., "Microwave Photonic Integrated Circuits for Millimeter-Wave Wireless Communications," Journal of Lightwave Technology, vol. 32, no. 20, pp. 3495–3501, Oct. 2014, doi: 10.1109/JLT.2014.2321573.
[5] C. C. Renaud, M. Natrella, C. Graham, J. Seddon, F. Van Dijk, and A. J. Seeds, "Antenna Integrated THz Uni-Traveling Carrier Photodiodes," IEEE Journal of Selected Topics in Quantum Electronics, vol. 24, no. 2, pp. 1–11, Mar. 2018, doi: 10.1109/JSTQE.2017.2725444.
[6] G. Carpintero, R. C. Guzmán, C. Gordón, G. Kervella, M. Chitoui, and F. Van Dijk, "Photonic Integrated Circuits for Radio-Frequency Signal Generation," Journal of Lightwave Technology, vol. 34, no. 2, pp. 508–515, Jan. 2016, doi: 10.1109/JLT.2015.2511040.
[7] G. Carpintero et al., "Photonic-Enabled Terahertz Phase Arrays Using Dielectric Rod Waveguides for 6G Wireless Communications," in 2024 IEEE/MTT-S International Microwave Symposium - IMS 2024, June 2024, pp. 74–77. doi: 10.1109/IMS40175.2024.10600234.

[8] E. Rouvalis et al., “High-speed photodiodes for InP-based photonic integrated circuits,” Opt. Express, OE, vol. 20, no. 8, pp. 9172–9177, Apr. 2012, doi: 10.1364/OE.20.009172.
[9] M. Anagnosti et al., “Record Gain x Bandwidth (6.1 THz) Monolithically Integrated SOA-UTC Photoreceiver for 100-Gbit/s Applications,” Journal of Lightwave Technology, vol. 33, no. 6, pp. 1186–1190, Mar. 2015, doi: 10.1109/JLT.2014.2372816.
[10] E. Rouvalis et al., “170 GHz Photodiodes for InP-based photonic integrated circuits,” in IEEE Photonics Conference 2012, Sept. 2012, pp. 88–89. doi: 10.1109/IPCon.2012.6358502.
[11] M. Deumer et al., “Waveguide-integrated photoconductive THz receivers,” in 2022 47th International Conference on Infrared, Millimeter and Terahertz Waves (IRMMW-THz), Aug. 2022, pp. 1–2. doi: 10.1109/IRMMW-THz50927.2022.9895580.

Reviewer #3

(Remarks to the Author)
As the authors state, I have no doubt that this work is highly important and has the potential to enable future miniaturization and cost reduction of terahertz systems. However, at this stage, the study is still in the phase of establishing the core technological components, and in my view it does not, by itself, generate a broad interest. Therefore, my conclusion remains unchanged: it would be more appropriate to submit this work to a more specialized journal rather than Nature Communications.

Version 3:

Reviewer comments:

Reviewer #4

(Remarks to the Author)

Reviewer #5

(Remarks to the Author)
The authors report on the development and demonstration of a single QW PIN structure allowing various modes of operation. This is an important milestone on the way towards the integration of optical, terahertz and electronic properties on a single chip built from possibly universal components – which is a topic and achievement certainly appropriate for Nature Communications.

The manuscript essentially builds on and synthesizes numerous long-term developments: while QW PIN structure is the core component, the various functionalities address quite a number of branches of photonics (optical amplification, terahertz generation using photo-mixing, terahertz down-conversion etc.) – it is hard to imagine the unbelievable amount of work behind all these studies. The manuscript thus contains an extreme amount of useful and interesting information.

A serious weakness of the manuscript is the unfocused style of the presentation – it took me quite some time to just decipher its main message. Another issue is that there are many unsupported or undeveloped claims. For example, at lines 105 - 107 it is written that “The values of tau_QW and tau_trans depend on the reverse bias voltage, as well as the composition and thickness of the heterostructure layers forming the QW PIN photodiode.” - since these dependencies are used throughout the manuscript, I would expect a formula or a reference. Particular numerical values falling from a clear sky excessively appear throughout the entire manuscript. For example, at lines 108 - 111 it is written that “Figure 1a presents the theoretically predicted frequency response of a reverse-biased QW PIN photodiode with an RC time constant of 1.55 ps, fabricated on the GaAs/AlGaAs QW PIC substrate used in our demonstrations. The substrate structure, detailed in Supplementary Fig. S1, provides estimated saturation velocities of 0.72×10^7 and 0.8×10^7 cm/s...” - it is not clear how any of these three physical values was determined.

Altogether, although the manuscript still needs quite some improvement (even at the price of its expansion), the subject is really important to deserve communication to a broader audience.

Reviewer #6

(Remarks to the Author)
The manuscript from the Jarrahi group reports on a new method of THz or sub-THz frequency generation by photomixing in GaAs/AlGaAs semiconductor heterostructures. The results comprise higher efficiency, lower noise figures as compared to other photomixing approaches as well as straightforward on-chip integration owing to the monolithic semiconductor process. The device architecture even allows for versatile operation modes ranging from detection, phase or amplitude modulation to the aforementioned main purpose, photomixing.
The principle of the device is straightforward and based on established semiconductor physics, and as such nothing new. However, the technological relevance of this work is very high owing to the potentially cost-effective implementation of THz

devices from communications to sensing to industry and medical applications. Moreover, it has potential in research owing to the possible broadband operation with the potential to generate short pulses.
In my view, the work suits Nature Communications in principle. However, I would like to see two points to be addressed to broaden the scope of the work:
- Mechanism. What (quantum?) model would describe the charge carrier dynamics and enable one to deduce suitable current densities? The authors refer to a preprint paper with their own model. How would, e.g., modulation doping of the structure affect the electron transport?
- Scalability / material platform. The strength as well as THz bandwidth achievable with the photocurrent depend crucially on the intrinsic properties of the charge carriers and thus semicondutor material as well as the geometry of the structure. Which other materials may be suitable and for what reasons?

Regarding the authors' exchange with Referee 1, my assessment is that the Referee's original concerns were justified and, as stated by the Referee, have been adressed to a sizeable extent with rigor. The Referee has some more open questions and I am a bit surprised by the confrontational nature of the authors' responses. The Refree's statement for example that a normalized noise figure (units: W/Hz) should not depend on measurement time is correct and I would like to see a solid response to that.
On the question of noise and power measurements in general, my view is that this is certainly important when benchmarking something intended as a device architecture against existing concepts. I feel however that questions of scaling and universality as raised by myself above are more central.

Version 4:

Reviewer comments:

Reviewer #5

(Remarks to the Author)
The authors addressed my concerns, the manuscript is now fit for Nature Communications.

Reviewer #6

(Remarks to the Author)
The authors have addressed my questions satisfactorily. In my opinion, the manuscript is now suitable for publication.

Open Access This Peer Review File is licensed under a Creative Commons Attribution 4.0 International License, which permits use, sharing, adaptation, distribution and reproduction in any medium or format, as long as you give appropriate credit to the original author(s) and the source, provide a link to the Creative Commons license, and indicate if changes were made.
In cases where reviewers are anonymous, credit should be given to 'Anonymous Referee' and the source.
The images or other third party material in this Peer Review File are included in the article's Creative Commons license, unless indicated otherwise in a credit line to the material. If material is not included in the article's Creative Commons license and your intended use is not permitted by statutory regulation or exceeds the permitted use, you will need to obtain

permission directly from the copyright holder.
To view a copy of this license, visit https://creativecommons.org/licenses/by/4.0/

Reviewer #1 (Remarks to the Author):

The authors present an optoelectronic platform for generating and detecting terahertz radiation. These integrated THz platforms are intriguing from a scientific standpoint and have great potential for addressing real-world applications. Unfortunately, I am not convinced that the results support the claims or align with the journal's scope in terms of completeness, impact, or performance compared to existing technology. Details are provided below. In summary, I see a number of shortcomings. First, the comparison of the device's performance to that described in the literature is unfair. Second, central components of the platform have not been presented, nor has it been made clear how they could be realized. Third, no more than two functionalities have been demonstrated on a single chip: SOA and the THz emitter. Consequently, there is insufficient demonstration of integration into working devices to substantiate the platform claim. I cannot recommend publishing this work as it is, I recommend major revisions.

We thank the reviewer for affirming the importance of integrated terahertz platforms, both from a scientific perspective and for their strong potential in addressing real-world applications. Regarding the reviewer's concerns, we believe there may be a misunderstanding about the scope of our claims. The concept of monolithically integrated optoelectronic components based on QW PIN structures has been established for more than three decades and is already offered by PIC foundries (e.g., Smart Photonics, MACOM, ZHIWEI Photonics, Sandia, WIN Semiconductors), providing lasers, SOAs, modulators, isolators, photodetectors, waveguides, couplers, multiplexers, demultiplexers, and beam splitters on the same QW PIN chip.

In this work, we neither aim nor claim to reinvent these established components. Instead, we demonstrate that high-performance terahertz emitters and detectors can also be realized using the same QW PIN structures, establishing QW PIN photodiodes as the foundation of a Monolithically Integrated Terahertz Optoelectronic (MITO) platform. **Stated differently, we do not claim to have integrated or demonstrated all photonic components already available in commercial QW PIN platforms (e.g., lasers, multiplexers, couplers, modulators).** We have included the list of optoelectronics components already provided by a QW PIN PIC foundry at the end of our responses, highlighting the availability, maturity and advancement of this technology. **These are a 3-decade technology that are already available through foundry processes.** The focus of our demonstrations are gain-enhanced terahertz sources and detectors and even when showing intensity and phase modulation in the QW PIN waveguide, we do not present this as a fully realized modulator, but rather as a proof that the same substrate used for efficient terahertz sources and detectors can also support efficient intensity/phase modulation. To avoid any possible misunderstanding that we claim the integration of all photonic components, we have revised the manuscript title to:

Terahertz Generation and Detection through Gain-Enhanced Interband Photomixing in Quantum Well Structures

We have also emphasized multiple times in the manuscript that monolithically integrated optoelectronic components based on QW PIN structures are already widely available:

While QW PIN photodiodes are well-established for photonic integrated circuit implementation, their potential for terahertz generation and detection has yet to be realized.

QWs embedded in PIN photodiodes—key elements of commercially available photonic integrated circuits (PICs)—enable monolithic integration of lasers, semiconductor optical amplifiers (SOAs), modulators, filters, demultiplexers, and other passive optical components.

These examples represent just a fraction of the many optoelectronic functionalities that can be obtained on the same QW PIN substrate, which, as demonstrated in this work, supports both high-efficiency terahertz generation and high-sensitivity terahertz detection. In fact, high-performance tunable lasers, SOAs, modulators, filters, demultiplexers, and other passive optical components monolithically integrated on QW PIN substrates are already available through commercial PIC foundry processes.

The availability of commercial PIC foundry processes providing lasers, SOAs, modulators, filters, demultiplexers, and other passive optical components on the same QW PIN photodiode substrate enables the monolithic integration of terahertz optoelectronic systems onto a single chip.

As previously mentioned, QW PIN photodiodes serve as the fundamental building blocks of commercially available PICs, enabling the integration of active and passive optical components onto the same substrate. Figures 1b–g illustrate how the same QWs, embedded within the intrinsic region of a PIN photodiode waveguide, can function as multiple optoelectronic components, including a terahertz source (Fig. 1b), a terahertz detector (Fig. 1c), an optical pump source (Fig. 1d), an optical amplifier (Fig. 1e), an intensity modulator (Fig. 1f), and a phase modulator (Fig. 1g).

To further clarify the manuscript's claims and scope of contributions, we have made several additional revisions, which are detailed in our responses to the reviewer's comments below.

- The abstract claims: "...to realize all terahertz photonic system components onto a single chip for the first time." In the introduction, it states: "Previous work on waveguide-coupled terahertz sources and detectors required external lasers to function." However, no measurements have been taken with at least one laser and a photodiode on a single chip. Therefore, integration of the components into a common platform has not been demonstrated; it has only been shown in parts. This is critical because of the potential for high crosstalk, or interaction through back reflections, between components that could drastically reduce performance.

As explained above, we believe there has been a misunderstanding regarding the scope of our claims. To provide greater clarity, we have revised the corresponding sentence in the abstract to read as follows:

Here, we demonstrate gain-enhanced interband photomixing in quantum well (QW) PIN photodiodes as an efficient mechanism for frequency-tunable terahertz generation and detection, achieving significant improvements in power efficiency and sensitivity over the state-of-the-art.

QWs embedded in PIN photodiodes—key elements of commercially available photonic integrated circuits (PICs)—enable monolithic integration of lasers, semiconductor optical amplifiers (SOAs), modulators, filters, demultiplexers, and other passive optical components. By establishing QW PIN photodiodes as the foundation of a Monolithically Integrated Terahertz Optoelectronic (MITO) platform, this work paves the way for compact, scalable terahertz optoelectronic systems with applications in high-speed data transfer, spectroscopy, and hyperspectral imaging.

Also, to better clarify our claims, the mentioned sentence in the introduction is changed to:

Previous work on waveguide-coupled terahertz sources and detectors has lacked compatibility with photonic integrated circuits (PICs) that incorporate both passive and active optical components [12-20].

While the scope of our manuscript does not include integrating the demonstrated terahertz sources and detectors with all possible optical components, we note that issues such as crosstalk and back reflections are already well addressed and solved in QW PIN foundry processes (e.g., through spatial separation of components and the use of on-chip optical isolators). For example, photonic integrated circuits based on QW PIN structures developed for optical communication and LiDAR applications have successfully employed waveguide arrays with multiple phase modulators integrated alongside SOAs and lasers [ref1].

[ref1] Guo, W., Binetti, P.R., Althouse, C., Mašanović, M.L., Ambrosius, H.P., Johansson, L.A. and Coldren, L.A., 2013. Two-dimensional optical beam steering with InP-based photonic integrated circuits. IEEE Journal of Selected Topics in Quantum Electronics, 19(4), pp.6100212-6100212.

- The claimed platform lacks passive waveguides and couplers. Low-loss waveguides and couplers are highly relevant for the claimed functionality, especially for more complex designs, as shown in Fig. 4(d). The layer stack shown suggests that the quantum-dot layer is also the waveguiding layer. In that case, the passive

components would also have to include the quantum-dot layer, which would result in extreme losses. Based on the information provided, I cannot identify a method to incorporate passive components into the device. Without such components, I cannot support the claim of a platform.

We agree with the reviewer that low-loss waveguides and couplers are essential for a monolithically integrated optoelectronic platform. Quantum well intermixing provides an effective means to realize low-loss waveguides and passive components—such as distributed Bragg reflectors, MMI couplers, optical cavities, and wavelength (de)multiplexers—on a QW PIN platform [ref2, ref3, ref4]. Several quantum well intermixing techniques are already employed in QW PIN-based foundry processes. We have now included this information, along with the relevant references, in the revised manuscript.

Furthermore, QW intermixing is a very effective way to obtain low loss waveguides and passive components such as distributed Bragg reflectors, MMI couplers, optical cavities, wavelength demultiplexers and multiplexers, on the same QW PIN substrate [ref2, ref3, ref4].

[ref2] Marsh, J.H., 1993. Quantum well intermixing. Semiconductor science and technology, 8(6), p.1136.

[ref3] Hou, L. and Marsh, J.H., 2016. Photonic integrated circuits based on quantum well intermixing techniques. Procedia Engineering, 140, pp.107-114.

[ref4] Ooi, B.S., McIlvaney, K., Street, M.W., Helmy, A.S., Ayling, S.G., Bryce, A.C., Marsh, J.H. and Roberts, J.S., 1997. Selective quantum-well intermixing in GaAs-AlGaAs structures using impurity-free vacancy diffusion. IEEE journal of quantum electronics, 33(10), pp.1784-1793.

- The demonstrated laser relies on end-facet coating. As a result, it cannot be integrated into this configuration or claimed as a component of the platform, although DBR or DFB structuring seems feasible.

While the scope of our manuscript does not include integrating the demonstrated terahertz sources and detectors with on-chip lasers, we respectfully disagree with the reviewer's assertion that the demonstrated Fabry–Perot laser cannot be integrated with our terahertz sources, detectors, SOAs, and other components on the same chip. Fabry–Perot lasers with cleaved end facets (with or without coatings) are already available through commercial QW PIN PIC foundries, and their beam can be readily coupled to other on-chip components via integrated couplers, as previously demonstrated. For example, references below [ref5, ref6] are examples of how Fabry–Perot lasers were integrated with passive waveguides and couplers. That being said, we agree with the reviewer that implementing DBR or DFB structures is feasible, as QW-based DBR and DFB lasers have been realized for decades and are currently offered as standard building blocks in commercial PIC foundries.

[ref5] Lemaitre, F., Kleijn, S., Millan-Mejia, A.J., Williams, K. and Dolores-Calzadilla, V., 2022. High density integration of semiconductor optical amplifiers in InP generic photonic integration technology. IEEE Journal of Selected Topics in Quantum Electronics, 28(6: High Density Integr. Multipurpose Photon. Circ.), pp.1-6.

[ref6] Alexander, J.K., Caro, L., Dernaika, M., Duggan, S.P., Yang, H., Chandran, S., Martin, E.P., Ruth, A.A., Anandarajah, P.M. and Peters, F.H., 2020. Integrated dual optical frequency comb source. Optics Express, 28(11), pp.16900-16906.

- Figure 4d is misleading because the final vision would not be possible with the components shown. No passive waveguides or couplers were demonstrated. Furthermore, phase modulation has been demonstrated with a couple of pi at the laser frequency. However, depicted beam steering at THz frequencies that uses a single multicolor laser source would require phase shift ranges of the order of the THz wavelength, which is at least two orders of magnitude higher, because all laser lines pass through the same phase shifter and experience a similar phase shift. This is certainly not possible with this integrated approach.

We thank the reviewer for highlighting these aspects of Fig. 4d, which helped us to further clarify and illustrate our vision. As noted in our response to an earlier comment of the reviewer, QW intermixing is an effective approach for realizing low-loss waveguides and couplers in a QW PIN platform, and is already available in commercial QW PIN-based foundry processes. It is correct that, in Fig. 4d, the two laser lines pass through the same phase shifter. However, they do not experience identical phase shifts due to the strong wavelength dependence of the phase shift, an inherent property of the quantum-confined Stark effect as governed by the Kramers–Kronig relations. While passing both laser lines through the same phase modulator can yield a net terahertz phase shift, we recognize that this point may be misleading to readers without extensive theoretical or experimental analysis, which lies beyond the scope of this manuscript. To address this, we have modified Fig. 4d and now present an envisioned design that independently controls the phases of the two laser tones generating the terahertz signal (see revised figure below).

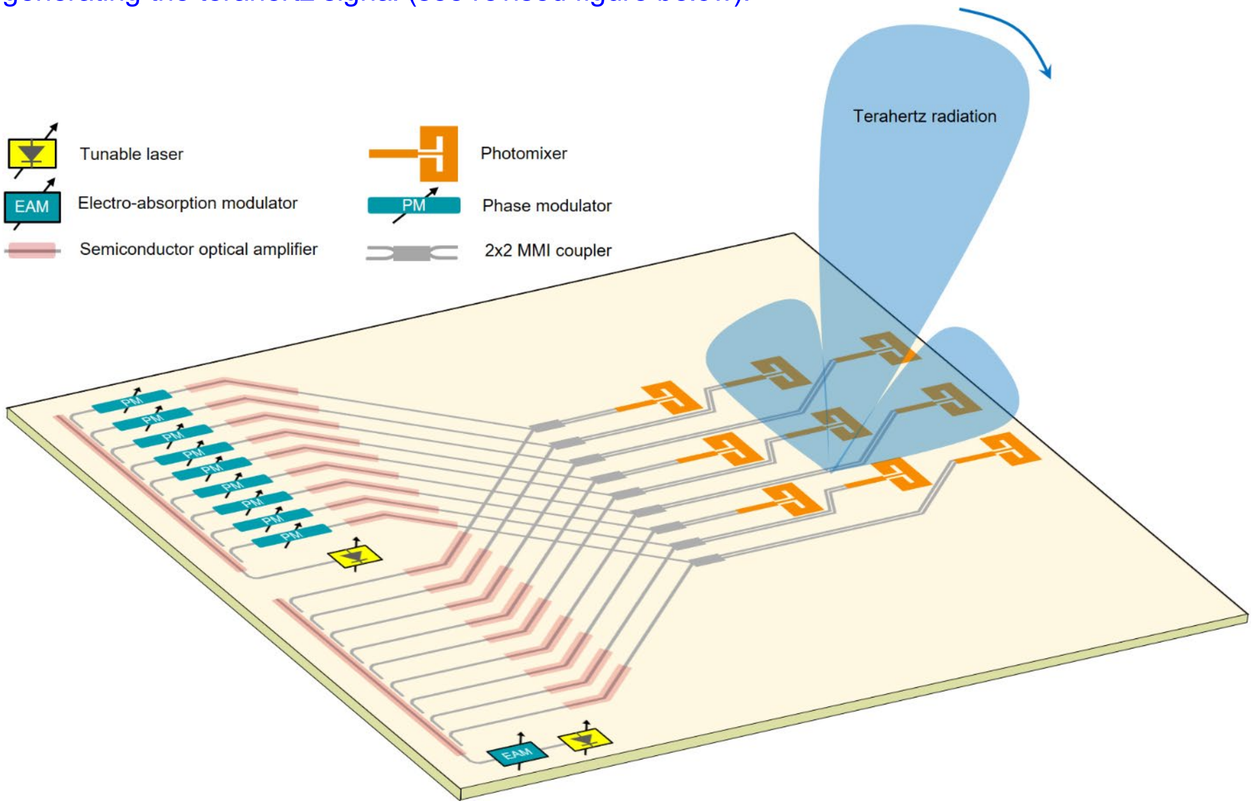

Fig. 4d, *Concept of a single-chip terahertz phased array transceiver based on a QW PIN PIC process for adaptive hyperspectral remote sensing and communication. A pair of tunable lasers, with an adjustable terahertz frequency difference, is used to generate optical pump beams with a tunable terahertz beat frequency. These optical beams are amplified by one (or multiple) SOAs and distributed across an array of photomixers, which function as both terahertz transmitters and receivers. Depending on the system requirements, phase and intensity modulators are used to control the intensity and phase of the optical beams pumping each photomixer. For example, to implement communication systems, intensity modulators encode the terahertz carrier signal with data to be transmitted. Furthermore, to implement radar, remote sensing, and smart communication systems, which require spatial scanning of the transmitted terahertz radiation, an array of phase modulators dynamically controls the terahertz phase at each photomixer, enabling tilt in the phase front of the transmitted terahertz radiation. The platform also enables implementing spectrometry, spectroscopy, and hyperspectral imaging systems, as the radiated terahertz frequency can be easily tuned by adjusting the laser wavelengths. Furthermore, multi-channel terahertz communication systems can be implemented using orthogonal frequency division multiplexing by employing multiple optical pump beams with different terahertz beat frequencies.*

- The phase and amplitude modulator appear to be the same device with the same driving signals. Based on the information provided, it can be concluded that phase and amplitude cannot be modulated independently. This drastically limits the device's usability and impact.

The reviewer is correct in noting the tradeoff between intensity and phase modulation. However, this tradeoff is intrinsic to any modulation process in a linear optical system, as amplitude and phase modulation cannot be fully decoupled due to the Kramers–Kronig relations. This limitation applies to modulators in all PIC platforms, including those based on III–V quantum wells, lithium niobate, and silicon photonics. Importantly, this tradeoff has not hindered the usability or impact of such devices, as demonstrated in numerous photonic integrated

circuits, including optical phased arrays. As shown in Figures 4b and 4c, the modulation voltage required for a 2π phase shift produces relatively low intensity modulation, which does not compromise the operation of the terahertz phased array. Moreover, the QW platform inherently provides optical gain, allowing compensation for any unwanted modulator losses. While the tradeoff between phase and intensity modulation does not limit phased-array performance, we have nonetheless revised Fig. 4d to include an array of SOAs following the phase modulators to further compensate for potential losses.

The authors claim that their platform has advantages over other photonic integration platforms, such as reduced complexity and improved THz emitter and receiver performance. However, the figures of merit used here are uncommon and/or incomparable to those in other publications. Some of these are discussed in more detail below.

Emitter/PD:
- The authors claim that the THz emitter achieves a higher optical-to-THz efficiency than state-of-the-art technology. However, they include the SOA in this comparison and compare it with literature that did not use extra optical signal amplification. Therefore, this is not a fair comparison. To make a fair comparison, the efficiency of the photodetector (PD) without the SOA must be used.

Our terahertz emitter consists of an SOA monolithically integrated with a photomixer on the same PIC platform. **A unique advantage of this configuration is the ability to enhance the photomixing efficiency through stimulated photon emission within the same device—an option not available in other reported emitters**. In other reported emitters, additional optical amplification requires a separate amplifier component that cannot be monolithically integrated with the terahertz emitter, thereby increasing the size, cost, and complexity of the system. **The absence of this unique capability in other emitters does not mean that we need to take away this important feature of our terahertz emitter when benchmarking the performance of our device.** To better emphasize that the SOA is an integral part of our terahertz emitters and detectors—and a key feature enabling their high efficiency—we now describe the terahertz generation and detection process in the revised manuscript as '**Gain-Enhanced Interband Photomixing in QW structures**' and have added the following discussion:

Two key factors contribute to this performance enhancement. First, the relatively long interaction length between the optical pump beam and the QW photo-absorbing region (when compared with surface-illuminated photomixers), where the photoabsorbed carriers can escape from and transit to the P/N layers in a sub-picosecond timescale, enabling both high quantum efficiency and ultrafast photomixing. Second, the gain-enhanced photomixing process within a monolithically integrated platform, enhancing the terahertz generation/detection efficiency. While the QW PIN photomixer can generate/detect terahertz signals via interband photomixing when directly pumped by two lasers with a terahertz beat frequency, integrating an SOA significantly reduces the required optical power and enhances the terahertz generation/detection efficiency. This highlights a key advantage of QW PIN photodiodes enabling scalable, complex terahertz optoelectronic systems based on large source/detector arrays.

Furthermore, the comparison omits some current state-of-the-art publications, such as: [1], [2].

We thank the reviewer for suggesting these references. We added the results of these references to the comparison graph in Fig. 3a and supplementary table S1.

- Responsivity values and IV curves are not provided for the PD.

The photomixer's I–V characteristics are shown in Fig. 2e inset, which plots the photocurrent as a function of bias voltage and SOA pump current at an input optical power of 5 mW. As noted earlier, our terahertz source consists of a monolithically integrated SOA and photomixer. While the input optical power to the SOA section is known, the optical power coupled from the SOA to the photomixer cannot be directly measured because these sections are tightly integrated to form the gain-enhanced terahertz source. As a result, it is not experimentally feasible to extract the pure responsivity of the photomixer section of the device alone.

Detector:
- Although it is claimed that the detector surpasses state-of-the-art detectors, the provided comparison is invalid for multiple reasons:

o The comparison was only made to direct detectors. The presented receiver is a multiplicative mixer that uses an optical local oscillator (LO), but it is compared to direct detectors, which scale differently with respect to bandwidth. A more accurate comparison would include mixers with an electrical or optical local oscillator, such as Schottky diode-based subharmonic mixers or photoconductive mixers. Such mixers, as described in the literature, provide far superior sensitivity than that demonstrated here. [3], [4].

We thank the reviewer for this valuable suggestion. In the revised manuscript, we have removed the performance comparisons with direct detectors and instead compared our detector with other multiplicative mixers that utilize an optical local oscillator (LO), demonstrating the higher sensitivity achieved by our device. The figure on the right replaces Fig. 3b in the revised manuscript, and the relevant references have been added accordingly [ref7, ref8].

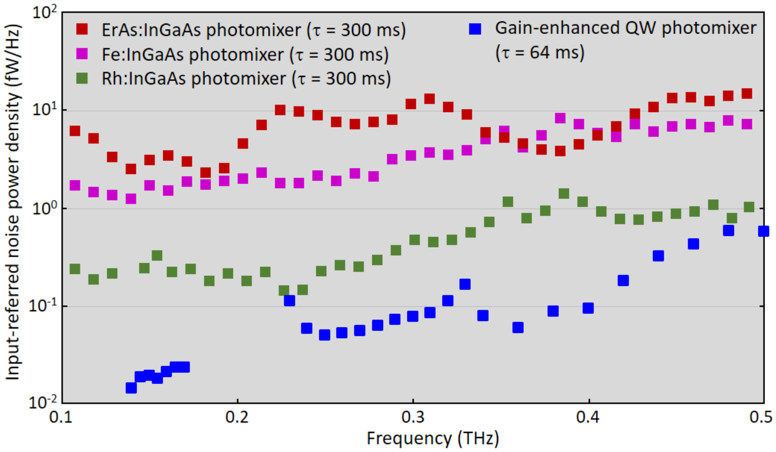

[ref7] Deumer, M., Breuer, S., Berrios, S., Keyvaninia, S., Schwanke, G., Schwenson, L., Lauck, S., Liebermeister, L., Nellen, S., Schell, M. and Kohlhaas, R.B., 2024. Continuous wave THz receivers with rhodium-doped InGaAs enabling 132 dB dynamic range. Optics Express, 32(17), pp.29855-29867.

[ref8] Fernandez Olvera, A.D.J., Roggenbuck, A., Dutzi, K., Vieweg, N., Lu, H., Gossard, A.C. and Preu, S., 2019, February. International system of units (SI) traceable noise-equivalent power and responsivity characterization of continuous wave ErAs: InGaAs photoconductive terahertz detectors. In Photonics (Vol. 6, No. 1, p. 15). MDPI.

o Unsuitable Measurement Scheme: The input-referred noise (also known as sensitivity or NEP) of a mixer is not a function of integration time or bandwidth. However, due to the radiometer measurement scheme that was applied (bandpass filter and power detector), the value appears to be much lower than it actually is. It also scales with the square root of the integration time. This makes the comparison with other detectors invalid and is strongly misleading. For example: Using the bandpass filter (BW: 140 MHz) and an integration time of 0.1 s, the input-referred noise of the mixer itself is 3,700 times higher than indicated by Fig. 2(h), contradicting the claimed sensitivity.

We believe the reviewer may have misunderstood the setup and procedure used for the detector sensitivity measurements. As detailed in the Methods section and illustrated in Supplementary Fig. S10b, the detector performance was characterized using two parallel paths: (1) a path including a bandpass filter and RF power meter to measure the detector's conversion loss (not sensitivity), and (2) a path including a spectrum analyzer to evaluate the detector's sensitivity. The spectral analysis was performed using both an electrical spectrum analyzer and a spectral lock-in detection code to enhance the SNR. To clarify this further, we have expanded the Methods section with additional details on the spectral lock-in detection process.

To perform spectral lock-in detection, the input terahertz signal is first modulated, and the electrical spectrum analyzer is replaced with a real-time lock-in detection analyzer. This analyzer down-converts the IF spectrum from ~0.8 GHz to ~40 MHz using an RF mixer (RFCOMP HD26217) and then digitizes it with an analog-to-digital converter (Texas Instruments ADS5485). A Fast Fourier Transform is then applied to the time-domain signal, decomposing it into distinct frequency components spanning 0–100 MHz. All frequency components are demodulated in parallel to generate the lock-in detected spectrum.

Using spectral lock-in detection, the background noise level is reduced while preserving all spectral information, thereby lowering the input-referred noise and improving sensitivity. In the original manuscript, our spectral lock-in detection code applied lock-in detection to the spectral power data, yielding a 5 dB/dec noise reduction rate with integration time. In the revised manuscript, we repeated the detector measurements using a modified spectral lock-in detection code that applies lock-in detection to the spectral voltage data (consistent with refs. 7 and 8, as suggested by the reviewer), which results in a 10 dB/dec noise reduction rate with integration time. Consequently, the revised manuscript shows lower input-referred noise levels, as illustrated in the updated Fig. 2h.

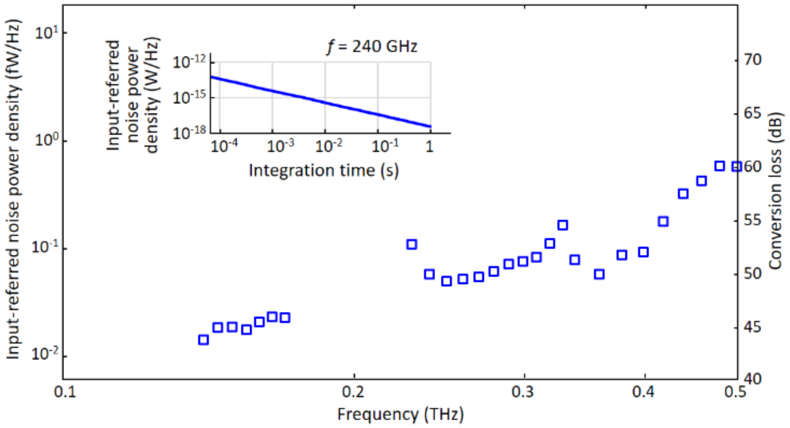

Fig. 2h, *Conversion loss and input-referred noise power density at a bias voltage of -0.7 V, photocurrent of 0.38 mA, and an integration time of 64 ms. Spectrum of the down-converted signal is processed using a real-time spectral lock-in detection code (see Methods and Supplementary Fig. S6), which offers an input-referred noise power density reduction rate of 10 dB per decade as a function of integration time while preserving spectral information. Inset shows the dependence of the input-referred noise power density on the integration time at 240 GHz for a 15 kHz modulation frequency.*

To further clarify the dependence of sensitivity on integration time, we have also added Supplementary Fig. S6.

We also added the following text to the manuscript:

Spectrum of the down-converted signal is processed using a real-time spectral lock-in detection code (see Methods and Supplementary Fig. S6), which offers an input-referred noise power density reduction rate of 10 dB per decade as a function of integration time while preserving spectral information (Fig. 2h inset).

We respectfully disagree with the reviewer's concern that characterizing our detector using lock-in detection makes the comparison with other detectors invalid or misleading, as the majority of reported terahertz detectors also employ lock-in detection. For example, Refs. 7 and 8—recommended by the reviewer as comparable photomixer-based terahertz detectors using an optical LO—both relied on lock-in detection codes to analyze their down-converted terahertz signals, with an integration time of 300 ms.

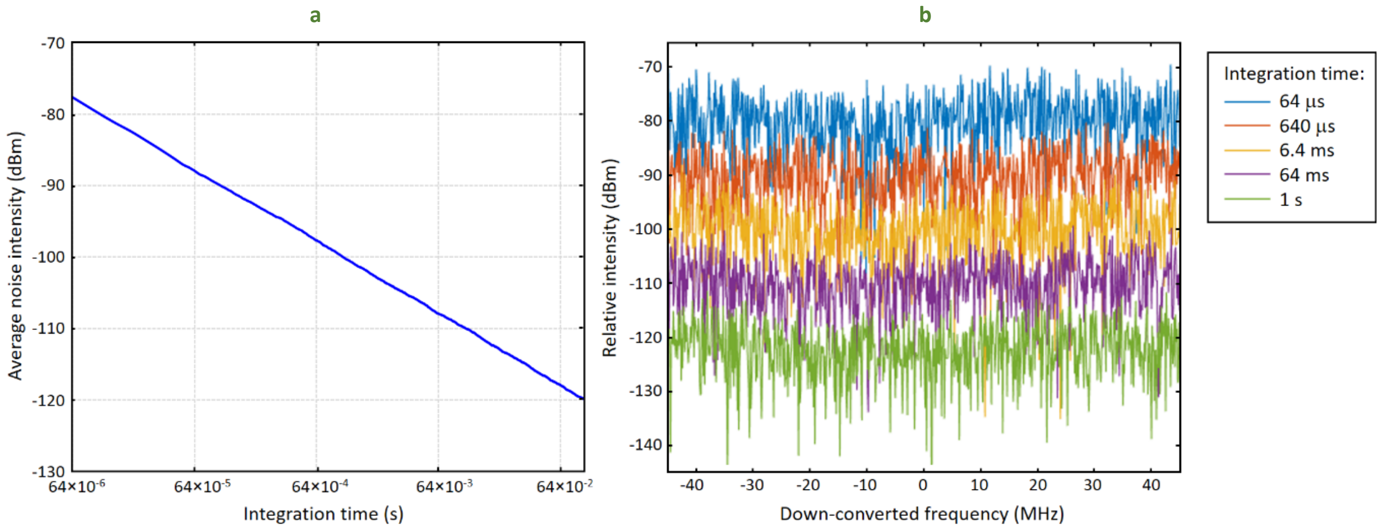

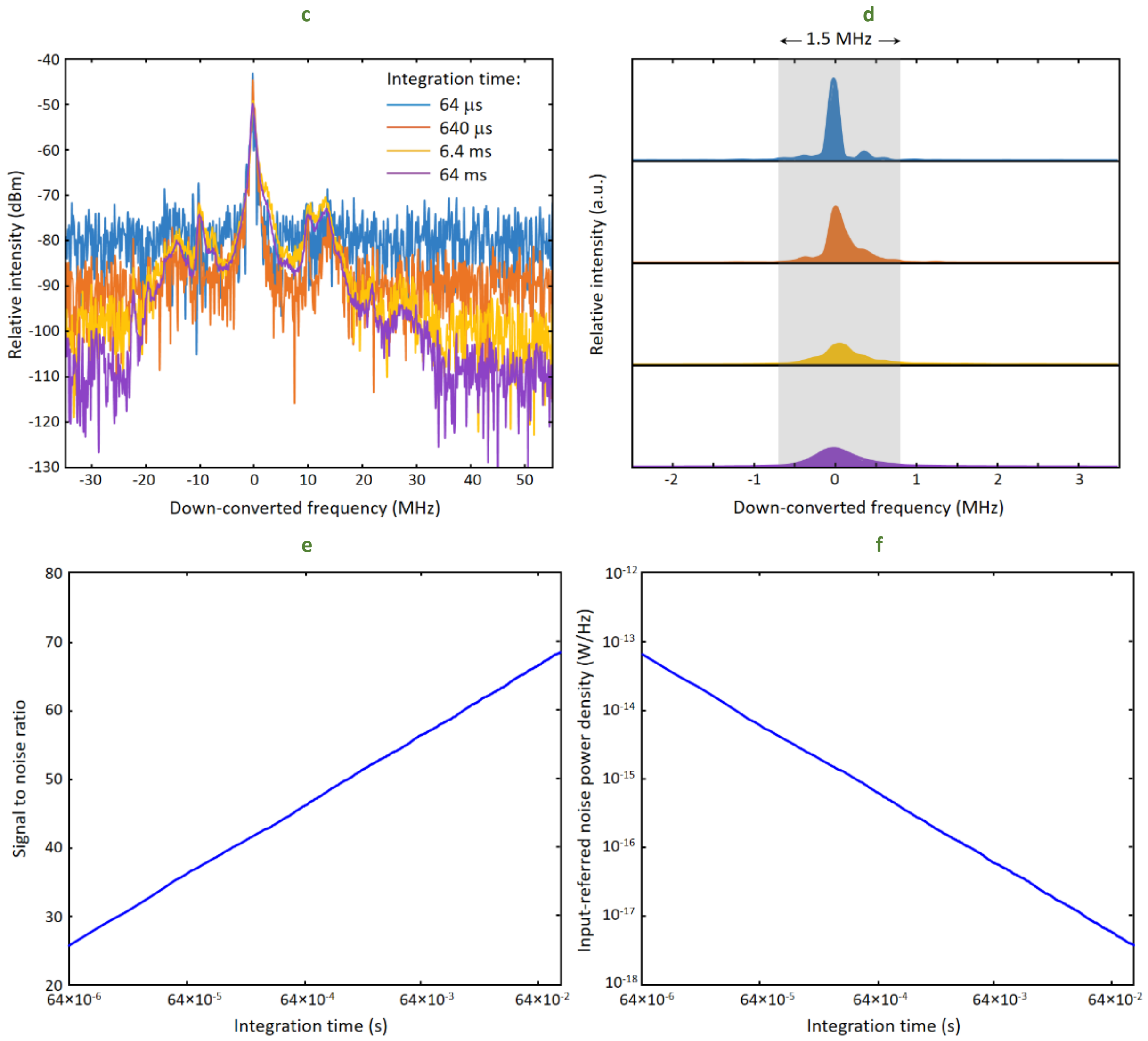

Fig. S6. Characteristics of the down-converted terahertz spectrum using spectral lock-in detection. a, *Average noise intensity of the down-converted spectrum as a function of integration time, showing a noise reduction rate of 10 dB per decade.* ***b,*** *Down-converted spectrum in the absence of a terahertz input for integration times ranging from 64 µs to 1 s.* ***c,*** *Down-converted spectrum of a 240 GHz input tone at –14.5 dBm for integration times from 64 µs to 64 ms.* ***d,*** *Down-converted signal power within a 1.5 MHz detection bandwidth remains constant for integration times up to 64 ms, despite IF spectral broadening caused by pump laser wavelength fluctuations.* ***e,*** *Signal-to-noise ratio (SNR) of the down-converted 240 GHz tone at –14.5 dBm, calculated as the ratio of signal power to noise power within a 1.5 MHz bandwidth, for integration times ranging from 64 µs to 1 s.* ***f,*** *Calculated input-referred noise power density, defined as the input power required to yield an SNR of 1 in a 1 Hz bandwidth (*$\frac{-14.5\ dBm - SNR}{1.5\ MHz}$*).*

o Missing: Comparison with the authors' own previous work. The authors did not compare their results to those of their previous publication, in which they used a very similar measurement scheme and detector principle. This would be a fair comparison. However, the sensitivity value provided in the previous publication is over 100,000 times better than what was demonstrated here. This disproves the claim of state-of-the-art performance.

As described above, the setup, measurement scheme, and operating conditions used in this work differ significantly from those in Wang et al.. Therefore, a direct comparison between our results and the Y-factor noise temperature measurements reported by Wang et al. is not straightforward and fair.

o Discrepancy Between Figures 2h and 3b: In Figure 3b, the noise equivalent power is plotted in pW/√Hz for comparison with direct detectors. How is the unit fW/√Hz in Fig. 2(h) converted to pW/√Hz in Fig. 3(b)?

There was no discrepancy between Figures 2h and 3b in the original manuscript. These figures presented the detector sensitivity in two different forms: in Fig. 2h, as input-referred noise power density with units of fW/Hz (not fW/√Hz), which—as the reviewer noted—is the appropriate metric for a photomixer-based detector with an optical LO; and in Fig. 3b, as sensitivity in units of pW/√Hz to enable performance comparison with direct detectors. Importantly, both figures were derived from the same spectral data. However, following the reviewer's earlier comment, we have removed the comparison with direct terahertz detectors, and in the revised manuscript both figures now present the same sensitivity parameter and unit.

- No IV curves are provided as a function of optical power that would show the conductivity modulation used in the mixing process.

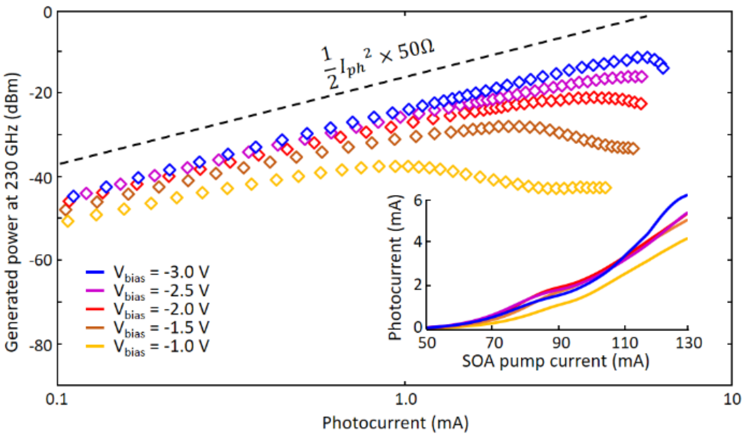

The I–V curves are shown in the inset of Fig. 2e (right). Since the same device is used for both terahertz generation and detection, the I–V characteristics are identical. It should be noted that, in QW-PIN photomixers, the photomixing process does not occur through conductivity modulation. Conductivity modulation is the mechanism in photoconductive photomixers, such as those described in Refs. 7 and 8. As mentioned in our response to an earlier comment, the terahertz detector consists of a monolithically integrated SOA and photomixer. While the input optical power to the SOA section is known, the optical power transferred from the SOA to the photomixer cannot be directly measured because the two sections are tightly integrated to form our gain-enhanced terahertz source. Consequently, it is not experimentally feasible to extract the isolated responsivity of the photomixer section.

Modulators:
Common figures of merit, such as the modulators' bandwidth and insertion loss, should be provided.

As noted in our response to an earlier comment of the reviewer, we have not claimed to implement intensity or phase modulators in this manuscript. Our intent is only to demonstrate that the QW PIN substrate can introduce phase and intensity modulation on the same platform used to realize high-efficiency terahertz sources and detectors. If we were aiming to demonstrate an intensity/phase modulator, we would have at least included a GSG or GS pad connected to the device to be able to measure modulation speed, as well as modulator waveguides of varying lengths to evaluate insertion loss.

Phase and intensity modulators based on QW PIN structures have been well established for more than three decades and are already offered by PIC foundries (e.g., Smart Photonics, MACOM, ZHIWEI Photonics, Sandia, WIN Semiconductors) alongside lasers, SOAs, isolators, photodetectors, waveguides, couplers, multiplexers/demultiplexers, and beam splitters. **In this work, we do not aim or claim to reinvent these existing optoelectronic components**. Rather, we show that high-performance terahertz emitters and detectors can also be realized using the same QW PIN structures, thereby establishing QW PIN photodiodes as the foundation of a Monolithically Integrated Terahertz Optoelectronic (MITO) platform. Put differently, **we do not claim to have integrated or demonstrated all components of a commercially available QW PIN platform (e.g., modulators), which are already mature technologies supported by foundry processes**.

Below, we provide an example list of optoelectronics components already provided by a QW PIN PIC foundry, highlighting the maturity and advancement of this technology.

Active Devices

Device	Parameter	Value
**Amplifiers and Lasers**		
Semiconductor Optical Amplifier	Small-Signal Gain	**30 dB/mm**
	Saturation Power (3dB)	**15 dBm**
	Max Injection Current	200 mA/mm
	Noise Figure	5 dB
DFB Laser[2,3]	Tuning Range (temperature control)	3 nm
	Output Power[4]	**20 mW @100 mA**
	Threshold Current	**10 mA**
DBR Laser[3]	Tuning Range[5]	4 nm
	Output Power[4]	**>10 mW @100 mA**
	Linewidth	< 500 kHz
	Threshold Current	**14 mA**
SG-DBR Laser[3]	Tuning Range	30 nm
	Output Power[4]	**> 10 mW @100 mA**
	Linewidth	< 500 kHz
	Threshold Current	**15 mA**
Low Linewidth Tunable Laser[3,6]	Tuning Range	35 nm
	Output Power incl. booster SOA	20 mW @100 mA
	Threshold Current	40 mA
	Linewidth	< 100 kHz
Fabry-Perot Laser[3]	Output Power[4]	20 mW @100 mA
**Modulators**		
Electro-Optical phase modulator (MZI)	Insertion Loss	2 dB
	Bandwidth	**25 GHz**
	Vπ	5 V
	Extinction Ratio	> 25 dB
Current Injection Phase control[7]	Loss	< 0.5 dB for 1 mm
Electro-Absorption modulator (EAM)	Insertion Loss	**2 dB**
	Bandwidth	**> 50 GHz**
	Vop	**3 V**
	Extinction Ratio	> 6 dB

Device	Parameter	Value
**Photodiodes**		
PIN Photodiodes	3 dB bandwidth	**30 GHz**
	Dark current	25 nA @ -2 V Bias
	Responsivity	0.85 A/W

Passive Devices

Device	Parameter	Value
**Passive waveguides**		
Shallow Waveguide	Loss	1.5 dB/cm
	Minimal Bending Radius	400 µm
Deep Waveguide	Loss	2 dB/cm
	Minimal Bending Radius	100 µm
	Curved Deep Waveguide Loss	0.1 dB/90°
**Gratings**		
Uniform Grating	Coupling Factor	50 /cm
	Current Controlled Tuning Range	2 nm
Sampled Grating	Coupling Factor	50 /cm
**Broad Band Reflectors**		
1x2 MMI Reflector	Loss	2 dB
	Reflectivity	40 %
1x1 MMI Reflector	Loss	2 dB
	Reflectivity	60 %
**Couplers**		
1x2 MMI coupler	Loss	**0.5 dB**
2x2 MMI coupler	Loss	**0.5 dB**
**Other features**		
Active-Passive Transition	Loss	<< 0.1 dB
	Reflectivity	-50 dB
Isolation Section	Loss	0.1 dB

[1] M. Deumer et al., "Ultra-Wideband PIN-PD THz Emitter with > 5.5 THz Bandwidth," J Infrared Milli Terahz Waves, vol. 45, no. 9, pp. 831–840, Oct. 2024, doi: 10.1007/s10762-024-01001-z.
[2] M. Grzeslo et al., "High saturation photocurrent THz waveguide-type MUTC-photodiodes reaching mW output power within the WR3.4 band," Opt. Express, OE, vol. 31, no. 4, pp. 6484–6498, Feb. 2023, doi: 10.1364/OE.475987.
[3] "Virginia Diodes, Inc - Mixers." Accessed: Jul. 16, 2025. [Online]. Available: https://www.vadiodes.com/en/products/mixers-shm-ehm-and-fm
[4] M. Deumer et al., "Continuous wave THz receivers with rhodium-doped InGaAs enabling 132 dB dynamic range," Opt. Express, OE, vol. 32, no. 17, pp. 29855–29867, Aug. 2024, doi: 10.1364/OE.532465.
[5] K. Rohlfs and T. L. Wilson, Tools of Radio Astronomy. in Astronomy and Astrophysics Library. Berlin, Heidelberg: Springer, 1996. doi: 10.1007/978-3-662-03266-4.
[6] N. Wang, S. Cakmakyapan, Y. J. Lin, H. Javadi, and M. Jarrahi, "Room-temperature heterodyne terahertz detection with quantum-level sensitivity," Nature Astronomy 2019 3:11, vol. 3, no. 11, pp. 977–982, Jul. 2019, doi: 10.1038/s41550-019-0828-6.

Reviewer #2 (Remarks to the Author):

In the present manuscript the authors demonstrate an integrated optoelectronic terahertz platform that integrates THz source, detector, modulator and other passive components on one chip, all based on PIN quantum well photodiodes.

While the presented platform is with no doubt very powerful and elegant, I have several questions and concerns which I will outline in the following.

We sincerely thank the reviewer for highlighting the strengths of our presented platform. Our detailed responses to the reviewer's comments are provided below.

Page 2: The sentence on "biological tissues" may deserve a remark about the problem of water absorption, ubiquitous in biological systems.

We thank the reviewer for raising this important point. The sentence has been revised as follows:

In medical imaging, the low scattering of terahertz waves in biological tissues enables applications such as tumor detection and vascular mapping. However, the strong absorption by water—prevalent in biological systems—should be carefully considered when defining the scope and practical implementation of terahertz medical imaging systems.

Page 4: About this sentence: "This limitation arises because previous studies have been limited to photomixing schemes utilizing intersubband photon absorption in QW PIN photodiodes 23,24":
Refs. 23 and 24 describe two different schemes, but neither of them is based on a PIN photodiode (but rather on unipolar structures).

We thank the reviewer for catching this mistake. The sentence has been revised as follows:

Previous studies have been limited to photomixing schemes utilizing intersubband photon absorption in QW structures [23, 24].

Page 5: Usually photomixers rely on the ultrafast carrier relaxation/recombination across the band gap (like in low-T grown GaAs). Here the transit time apparently takes this role. It would be good to make a remark on this difference compared to standard photomixers.

We thank the reviewer for raising this important point and suggestion. We have added the following sentence to the revised manuscript in the section discussing the frequency response of the QW PIN photodiode:

Comparing this frequency response with that of conventional photomixers, which rely on ultrafast carrier relaxation and recombination across the bandgap, reveals that the carrier transit time in QW PIN photodiodes effectively replaces the role of carrier lifetime in conventional photomixers.

Page 6 top: why are the saturation velocities for electrons and holes nearly the same, considering the vastly different effective masses? Is there a simple argument for this?

The velocity–electric field characteristics for electrons and holes in GaAs are shown in the figure below [ref9]. At low electric fields, electrons travel faster than holes due to their smaller effective mass. At higher electric fields, however, electrons exhibit a velocity overshoot that is not observed for holes, leading to nearly identical saturation velocities for both carriers. The absence of velocity overshoot in holes is attributed to their larger effective mass and strong interband scattering, which promote rapid momentum and energy relaxation and prevent the non-equilibrium distribution required for overshoot. In contrast, electrons, being lighter and subject to weaker scattering in the conduction band, are more susceptible to ballistic acceleration and velocity overshoot.

[FIGURE REDACTED]

[ref9] Schwierz, F., Pezoldt, J. and Granzner, R., 2015. Two-dimensional materials and their prospects in transistor electronics. Nanoscale, 7(18), pp.8261-8283.

Page 6: what does this half-sentence mean: “Leveraging the ultrafast carrier dynamics that support interband photon absorption“? What does the absorption have to do with the ultrafast carrier dynamics?

We thank the reviewer for pointing out this confusing sentence. We have revised it in the manuscript for improved clarity:

Leveraging the ultrafast dynamics of carriers generated through interband photon absorption, an optical pump beam containing two frequency components separated by a terahertz frequency difference can generate a terahertz photocurrent through photomixing.

Page 7: The SOAs are introduced without any particular reasoning. Not being an expert in integrated photonics, I was asking myself whether one always needs an SOA, or if just two lasers could be sufficient. Are DFB lasers never powerful enough (see also next question) ? It would be good to give some more explanations on this issue.

We thank the reviewer for noting the need for further clarification on the role of the SOA. While the QW PIN photomixer can generate and detect terahertz signals via interband photomixing when directly pumped by two lasers with a terahertz beat frequency, integrating an SOA significantly lowers the required optical power and enhances the efficiency of terahertz generation and detection. This underscores a key advantage of QW PIN photodiodes—their ability to support gain-enhanced photomixing within a monolithically integrated platform—thus enabling scalable and complex terahertz optoelectronic systems based on large source/detector arrays. In the revised manuscript, we have added further clarification to emphasize these points:

Figure 2a illustrates a fabricated terahertz source/detector prototype that generates and detects terahertz signals via gain-enhanced photomixing within a GaAs/AlGaAs QW PIN photodiode.

While the QW PIN photomixer can generate/detect terahertz signals via interband photomixing when directly pumped by two lasers with a terahertz beat frequency, integrating an SOA significantly reduces the required optical power and enhances the terahertz generation/detection efficiency. This highlights a key advantage of QW PIN photodiodes enabling scalable, complex terahertz optoelectronic systems based on large source/detector arrays.

Page 8: In this connection I see another problem: despite the promise of a completely integrated platform, the authors finally use two external lasers with slightly different wavelengths as pump sources. The claim that they also could be integrated sounds a bit too casual (“Two commercially available lasers are used to characterize both the SOA and the monolithically integrated terahertz source/detector. The two lasers can be replaced by

two wavelength tunable lasers fabricated on the same QW PIN photodiode substrate 31"). Indeed Ref. 31 presents such a laser pair (power 15 mW !) – but would this really be so easy to be implemented in the present platform ? If yes, why isn't this demonstrated then? If no, an honest discussion should be given.

As the reviewer noted, Ref. 31 (Ref. 34 in the revised manuscript) demonstrates such a tunable laser pair on exactly the same commercially available substrate that we used to fabricate our terahertz sources and detectors. Thus, an identical laser pair could be monolithically integrated with our terahertz devices. Achieving this integration would only require developing and adding the fabrication step for distributed Bragg reflectors, a well-established process that could be implemented with minimal risk. We chose not to pursue this route, as fabricating the lasers ourselves would have amounted to re-demonstrating a well-known, four-decade-old technology without providing new scientific insights, while delaying the dissemination of our novel results on gain-enhanced QW PIN terahertz sources and detectors.

That being said, the natural next step following this work is the realization of complex terahertz imaging, sensing, and communication systems on a single chip using QW PIN terahertz optoelectronics. Achieving this vision will indeed require monolithic integration of lasers with the terahertz devices. However, we are not fabricating these lasers ourselves, since commercial III–V PIC foundries already provide them (e.g., lasers) as standard PDK components on QW PIN substrates, where terahertz sources and detectors can be co-fabricated alongside other passive and active elements.

The availability of commercial PIC foundry processes providing lasers, SOAs, modulators, filters, demultiplexers, and other passive optical components on the same QW PIN photodiode substrate enables the monolithic integration of terahertz optoelectronic systems onto a single chip.

As previously mentioned, QW PIN photodiodes serve as the fundamental building blocks of commercially available PICs, enabling the integration of active and passive optical components onto the same substrate. Figures 1b–g illustrate how the same QWs, embedded within the intrinsic region of a PIN photodiode waveguide, can function as multiple optoelectronic components, including a terahertz source (Fig. 1b), a terahertz detector (Fig. 1c), an optical pump source (Fig. 1d), an optical amplifier (Fig. 1e), an intensity modulator (Fig. 1f), and a phase modulator (Fig. 1g).

In fact, high-performance tunable lasers, SOAs, modulators, filters, demultiplexers, and other passive optical components monolithically integrated on QW PIN substrates are already available through commercial PIC foundry processes. This establishes QW PIN photodiodes as the core building block of a MITO platform, making it possible to realize chip-scale optoelectronic terahertz imaging, spectroscopy, and communication systems for the first time.

Fig. 2a: The eight used colors are somehow not different enough; it is difficult to distinguish and assigning the different layers.

We thank the reviewer for pointing out this color ambiguity. In the revised manuscript, we have increased the contrast among these colors for improved clarity.

The data in Fig. 3a and 3b are very impressive, showing that the present QW photomixer outperforms most (or all) devices in the literature, both for THz sources and detectors. Here the question arises, why? Do the authors have an intuitive or even substantiated explanation why their device is better than anything else produced during the past two decades? This may actually be hard to believe and one may wonder about calibrations etc.

We thank the reviewer for highlighting the strong performance of our terahertz sources and detectors. Two key factors contribute to this performance relative to the state of the art. We have added the following discussion to the revised manuscript to highlight these factors:

Two key factors contribute to this performance enhancement. First, the relatively long interaction length between the optical pump beam and the QW photo-absorbing region (when compared with surface-illuminated photomixers), where the photoabsorbed carriers can escape from and transit to the P/N layers in a sub-picosecond timescale, enabling both high quantum efficiency and ultrafast photomixing. Second, the gain-enhanced photomixing process within a monolithically integrated platform, enhancing the terahertz generation/detection efficiency. While the QW PIN photomixer can generate/detect terahertz signals via interband photomixing when directly pumped by two lasers with a terahertz beat frequency, integrating an SOA significantly reduces the required optical power and enhances the terahertz generation/detection efficiency. This highlights a key advantage of QW PIN photodiodes enabling scalable, complex terahertz optoelectronic systems based on large source/detector arrays.

And a related question: can one and the same QW PIN structure really be a "Swiss army knife", i.e. be optimally suited for all different purposes: source, detector, modulator, SOA, filter, demultiplexer? I guess one has to make comprises concerning the performance for the different functionalities?

The reviewer has raised a very important question. While QW PIN structures possess the essential physical characteristics to support a wide range of functionalities—including terahertz sources and detectors via gain-enhanced interband photomixing, lasers and SOAs through stimulated photon emission, modulators leveraging the quantum-confined Stark effect, and passive components such as couplers, filters, and demultiplexers enabled by QW intermixing—it is challenging to design a single structure that simultaneously offers optimal performance for all these applications. For example, reducing the thickness of the QWs enhances photodetection speed by shortening carrier transit time, but reduces modulation depth. Similarly, higher P- and N-doping concentrations improve carrier injection for lasers and SOAs and accelerate photodetector response time, but at the cost of increased free-carrier absorption in passive devices.

These tradeoffs are well recognized, and commercial III–V foundries have already developed QW PIN structures that provide a balanced compromise across different functionalities. In our demonstration, we used a commercially available GaAs/AlGaAs QW PIC substrate to realistically account for these tradeoffs, even though it was not specifically optimized for terahertz generation and detection. Nonetheless, our results underscore the strong potential of QW PIN structures for high-performance terahertz generation and detection on a monolithically integrated platform, and indicate that further optimization of the QW design could deliver even greater performance gains. We have added this discussion to the revised manuscript.

While QW PIN structures possess the essential physical characteristics to support a wide range of functionalities—including terahertz sources and detectors via gain-enhanced interband photomixing, lasers and SOAs through stimulated photon emission, modulators leveraging the Quantum Confined Stark Effect, and passive components such as couplers, filters, and demultiplexers enabled by QW intermixing—it is challenging to design a single structure that offers optimal performance for all these applications. For instance, reducing the thickness of the QWs can improve photodetection speed by reducing carrier transit time, but at the expense of reduced modulation depth. Likewise, increasing P and N doping concentrations can enhance carrier injection for lasers and SOAs and improve photodetector response time, while simultaneously increasing free carrier absorption in passive devices. These tradeoffs are well recognized, and commercial III-V foundries have developed QW PIN structures that offer a balanced compromise across different functionalities. In our demonstration, we used a commercially available GaAs/AlGaAs QW PIC substrate to realistically account for these tradeoffs, even though it was not specifically optimized for terahertz generation and detection. Nonetheless, our results highlight the strong potential of QW PIN structures for high-performance terahertz generation and detection on a monolithically integrated platform, and suggest that further optimization of the QW design could yield even greater performance improvements.

We have also added the following statement to suggest potential directions for optimizing QW PIN structures for improved terahertz performance:

Thus, careful design of the QW PIN photodiode allows for minimizing τ_{RC} and τ_{trans}, reducing frequency roll-off and extending the device's operational range within the terahertz spectrum. For instance, reducing the

thickness of the intrinsic region while maintaining the same QW structures would decrease the carrier transit time while preserving QW properties for stimulated photon generation, amplification, and phase/intensity modulation. Similarly, reducing the device active area and thickness of the P/N layers as well as increasing their doping would lower the RC time constant while maintaining the QW properties.

Reviewer #3 (Remarks to the Author):

In this paper, the authors propose a concept for integrating terahertz photonic system components onto a single chip, using a quantum well structure as a key element. Among the various aspects, the most original point is the generation and detection of terahertz waves via photomixing using a QW PIN photodiode. Both experimental and theoretical evaluations have been conducted in detail, and the authors claim that they have achieved a power conversion efficiency exceeding that of conventional technologies. The device fabrication and evaluation methods have been carefully considered and are described thoroughly, making them credible. This represents an important technology that contributes to the miniaturization and cost reduction of terahertz technology.

We thank the reviewer for recognizing the novelty of our work on terahertz generation and detection using quantum-well PIN photodiodes, and for highlighting its potential impact on advancing miniaturized and cost-effective terahertz technologies.

One major concern, however, lies in the method of comparison with previous literature shown in Fig. 3. The power output values reported in previous works are basically the power of electromagnetic waves emitted into free space through an antenna. In contrast, the power evaluated in this paper is calculated from the current before it is converted into electromagnetic waves, meaning that the antenna conversion efficiency (loss) is not included. This does not constitute a fair comparison, and therefore does not adequately support the authors' claim of achieving higher conversion efficiency than conventional technologies. The same issue applies to the detector operation as well. This point needs to be corrected before considering publication in Nature Communications.

We thank the reviewer for raising this important point. Indeed, there are differences between power measurements where the generated terahertz power is emitted into free space through an antenna and those where it is probed on chip. Our comparisons with terahertz sources from the literature include both antenna-coupled and on-chip probed sources. To address the reviewer's concern, we have revised Fig. 3 to clearly distinguish between antenna-coupled and probed terahertz sources. Accordingly, we compare the generated terahertz power of our probed source with both categories from the literature, demonstrating higher conversion efficiency relative to each.

It should be noted that the GSG probe does not measure the terahertz current before conversion to electromagnetic waves. It measures the terahertz signal after conversion to electromagnetic waves. As illustrated in the figure on the right, the terahertz current is coupled from the device through the GSG pad to the probe tip, where it is converted into terahertz electromagnetic (EM) radiation that propagates along the output rectangular metallic waveguide of the GSG probe. In our measurements, we measured this EM wave propagating in the rectangular metallic waveguide output to determine the generated terahertz power. We then accounted for the probe's conversion loss (as specified by the vendor) and reported the generated THz signal on chip.

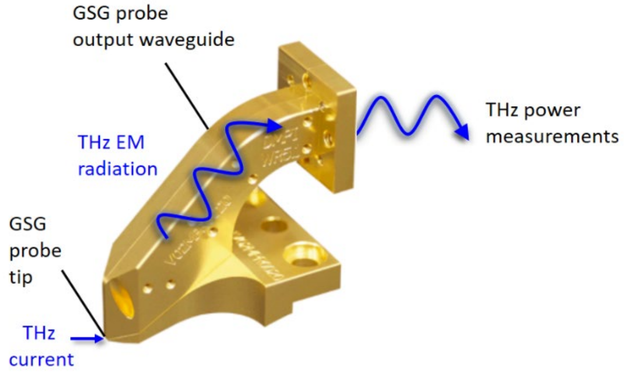

The GSG probe can also be used to convert our devices into antenna-coupled configurations, as illustrated in the figure on the right. In this scheme, the GSG probe converts the on-chip terahertz current into a terahertz electromagnetic wave that propagates through a rectangular metallic waveguide, which is then connected to a commercial metallic horn antenna to radiate into free space. Having the measured terahertz radiation power levels at the GSG probe's output waveguide and using the

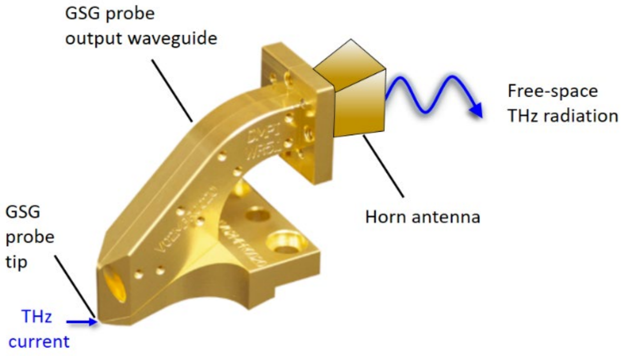

vendor-provided radiation efficiency of the horn antennas, we have projected the generated terahertz power levels for this antenna-coupled configuration.

We have added the generated terahertz radiation from our antenna-coupled device configuration to the comparison results shown in Fig. 3a. However, we note that this approach is not an efficient way to realize antenna-coupled terahertz devices from our QW PIN photomixers for two reasons (we expect better performance when integrating our devices with on-chip terahertz antennas):

(1) GSG probes combined with horn antennas exhibit lower radiation efficiency compared to conventional integrated antennas. This is illustrated in the graph on the right, which shows the measured radiation efficiency of our antenna system—comprising the GSG probe and horn antenna—across the three frequency bands used in our experiments.

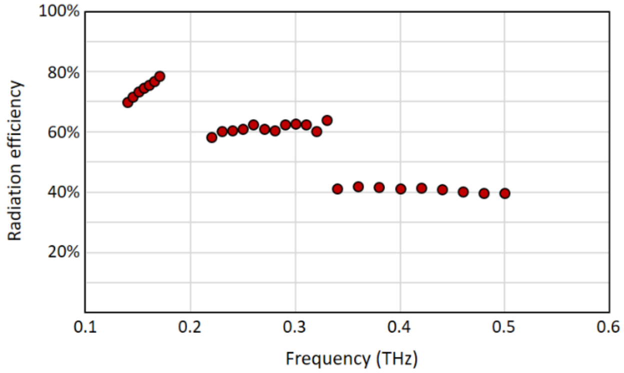

(2) Connecting our devices to a GSG probe requires the addition of a 130-µm-long GSG pad, which significantly attenuates the terahertz signal coupled to the probe. This pad will not be present when integrating our terahertz devices with on-chip antennas, where higher radiation powers can be achieved.

a

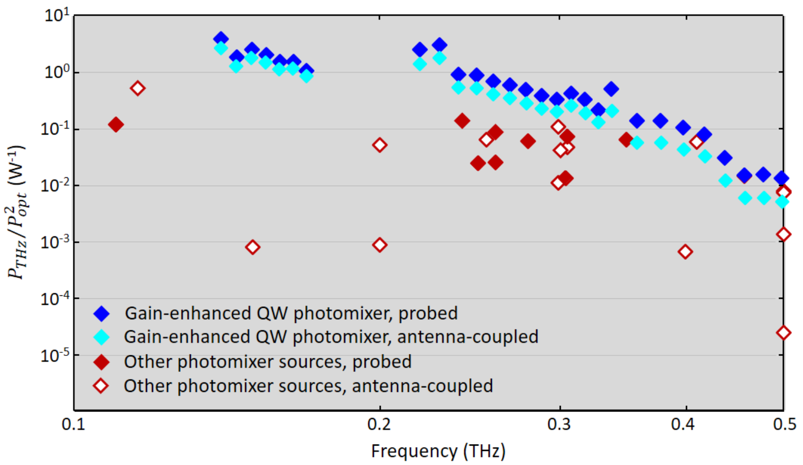

b

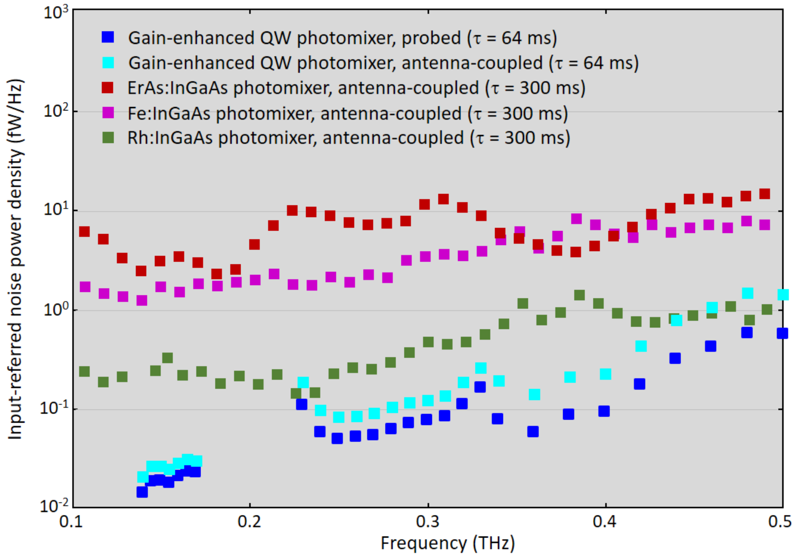

Fig. 3 | Performance comparison with the state-of-the-art. a, Comparison of the efficiency figure of merit P_{THz}/P^2_{opt} for the demonstrated gain-enhanced QW photomixer in terahertz generation mode with other photomixers from the literature (see Supplementary Table S1). The probed and antenna-coupled sources are represented by solid and open red diamonds, respectively. The GSG probes enable the conversion of the gain-enhanced QW photomixer into an antenna-coupled terahertz source. They extract the on-chip terahertz signal and launch it as an electromagnetic wave propagating through the rectangular metallic waveguide output of the probe, which can then be connected to a horn antenna to generate free-space terahertz radiation. By measuring the terahertz power levels at the probe waveguide output and using the specified radiation efficiency of commercial horn antennas, we projected the generated terahertz power levels and efficiencies for this antenna-coupled configuration, shown as cyan diamonds. b, Comparison of the input-referred noise power density (noise-equivalent power) of the demonstrated gain-enhanced QW photomixer in terahertz detection mode at a 64 ms integration time with other photomixers from the literature at a 300 ms integration time, including those based on ErAs:InGaAs, Fe:InGaAs, and Rh:InGaAs photomixers [35,36]. The GSG probes also enable operation of the gain-enhanced QW photomixer as an antenna-coupled terahertz detector. In this configuration, incident terahertz radiation is collected by a horn antenna and coupled into the rectangular metallic waveguide input of the GSG probe. The probe converts the guided terahertz wave into an electrical signal delivered to the GSG pads of the on-chip device. The projected input-referred noise power density values for this antenna-coupled scheme are represented by cyan squares.

The same considerations apply to the terahertz detector measurements. The incident terahertz signal is coupled as an electromagnetic wave propagating through the rectangular metallic waveguide of the GSG probe. The probe then converts this terahertz electromagnetic radiation into a voltage/current applied to the GSG pads connected to the on-chip device, introducing conversion loss. As with our terahertz source, we have added the detected terahertz radiation from our antenna-coupled device configuration to the comparison results shown in Fig. 3b. The revised Fig. 3 and its caption are provided above.

Additional comments are provided below:

•Regarding previous studies on detectors, the following paper is not included. This device is already commercially available and represents an important point of comparison: Ito, H., and Ishibashi, T.: “InP/InGaAs Fermi-level managed barrier diode for broadband and low-noise terahertz-wave detection”, Jpn. J. Appl. Phys., 2017, 56, (1), pp. 014101-1–014101-7

We thank the reviewer for reminding us of this important paper. In line with the comment from the first reviewer, who noted that comparisons with direct terahertz detectors are not appropriate, we have removed such comparisons (including this category of detectors) and instead provided comparisons with photomixer-based detectors employing an optical LO.

•The evaluation method for the phase modulator operation shown in Fig. 4c should be described in more detail.

We thank the reviewer for this valuable suggestion. This information has been added to the caption of Fig. 4c in the revised manuscript:

Optical phase modulation performance of the QW PIN waveguide, determined by measuring changes in the optical mode index as a function of the reverse bias voltage (bottom right inset). The top left inset depicts the experimental setup used for measuring the mode index changes. The input optical beam is first directed through a circulator and coupled into the waveguide using a lensed fiber. The optical beam reflected from the waveguide facet is then routed by the circulator to a photodetector. By modulating the reverse bias voltage applied to the waveguide and lock-in detection of the photodetector signal, the corresponding changes in the reflection coefficient of the waveguide mode are determined for each bias voltage. The optical mode index variations, Δn, are extracted from these changes in the reflection coefficient as a function of reverse bias voltage. Phase modulation per unit length is calculated as $2\pi\Delta n/\lambda$.

•Since the quantum-confined Stark effect changes both the absorption coefficient and the refractive index, using it as a phase modulator inevitably causes simultaneous modulation of the intensity. The authors should comment on whether this could pose a problem in future applications.

We thank the reviewer for this valuable suggestion. As noted, the quantum-confined Stark effect introduces both intensity and phase modulation. This dependence is inherent to any modulation process in a linear optical system, as amplitude and phase modulation cannot be fully decoupled due to the Kramers–Kronig relations. Modulators in other PIC platforms (e.g., III–V QWs, lithium niobate, and silicon photonics) also experience this tradeoff. Importantly, this does not limit device usability or impact, as demonstrated in numerous photonic integrated circuits, including optical phased arrays. Moreover, the QW platform provides optical gain to compensate for modulator losses when needed. While the inherent tradeoff between intensity and phase modulation does not restrict phased-array performance, we have revised Fig. 4d to include an array of SOAs following the phase modulators to offset potential losses. We have also added the following discussion to the revised manuscript:

As illustrated in Figs. 4b and 4c, the quantum-confined Stark effect induces both intensity and phase modulation—an intrinsic feature of any modulation process in linear optical systems. According to the Kramers–Kronig relations, and as demonstrated across various PIC platforms (including III-V quantum wells, lithium niobate, and silicon photonics), intensity and phase modulation are inherently coupled in linear regimes. Consequently, the tradeoff between the intensity and phase modulation must be carefully considered in the design of MITO systems. The ability of QW PIN PIC platforms to provide optical gain offers a key advantage, helping to mitigate this tradeoff and maintain high system performance.

Reviewer #1 (Remarks to the Author):

Thanks to the authors for revising the manuscript. The new title accurately reflects the content of the paper. I agree with the authors that demonstrating emitters and receivers based on the same QW PIN stack is an interesting work.

Many of the previous concerns were addressed, but important issues in the methods remain. These issues invalidate and mislead some of the comparisons and claims. This includes the publication's central claims about the sensitivity of the receiver. Below, I provide a brief summary of each critical aspect and have attached a more thorough explanation of each one.

We thank the reviewer for agreeing with us on the importance of monolithically integrated terahertz sources and detectors based on the same QW PIN stack. We are glad that many of the reviewer's previous concerns have been addressed. The reviewer's concerns about the sensitivity of the demonstrated detector and its comparison with other detectors are addressed below. Please note that we have changed the sequence of the reviewer's comments in our response document, as many of them were repeated several times. Please also note that Ref. 35 and 36 mentioned below, are the reference numbers in the previous version of the manuscript and they are Ref. 38 and 39 in the revised manuscript.

Receiver sensitivity
The authors claim that the receiver has a '…sensitivity over the state-of-the-art.' (l. 14). However, the results in this work were obtained using the evaluation scheme 'spectral lock-in' which differs significantly from the evaluation methods used in the other cited publications. The different evaluation method produces different results, rendering a fair comparison impossible. There are multiple indications in the presented data that support this claim. The most prominent indication is that the sensitivity of a receiver is a fixed value and therefore should not scale with integration time. However, the data in Fig. S6f and Fig. 2h show scaling with integration time, indicating that the authors used a radiometer scheme. That said, the 10 dB/decade increase in sensitivity with integration time (Fig. S6a, f, and 2h) raises concerns about signal evaluation. Normal lock-in produces constant sensitivity, whereas radiometer noise scales at 5 dB/decade. In contrast, the other publications (ref. 35 and ref. 36) use a standard lock-in scheme and demonstrate sensitivity independent of integration time, as expected. In summary, the authors obtain their sensitivity values differently than state-of-the-art publications. Consequently, their sensitivity appears to be orders of magnitude higher and thus beyond state of the art, which is an unsubstantiated claim. Furthermore, their previous publications with comparable methods but far better results are missing.

For better clarity, we separately address two different concerns stated above: (1) concerns about benchmarking the detector performance compared with other detectors in the literature; and (2) concerns about the spectral lock-in detection method and its integrity.

(1) Benchmarking and comparison with other detectors in the literature: Benchmarking a new technology is not always straightforward, since there are always some aspects of the work that differ from the prior art. Meanwhile, if all aspects are the same, there is no novelty to be published. Benchmarking is particularly tricky for terahertz detectors, since there are several different types of detectors in the literature that can be divided into different categories: (A) coherent (referenced detectors) vs. incoherent (direct detectors); (B) electrical, optical, or thermal detectors; (C) broadband vs. narrowband detectors; (D) free-space-coupled vs. waveguide-coupled vs. on-chip-probed detectors; (E) active vs. passive detectors; and (F) electric field vs. absolute power detectors. Therefore, no matter which category we pick for comparison, one can question the fairness of the comparison because the compared detectors do not have exactly the same features and operation principles.

In the original manuscript, we compared our detector's performance with other incoherent (direct) terahertz detectors. These are detectors that are completely independent of the radiation source and do not require any coherence, synchronization, referencing, or locking between the radiation source and detector. Even within that category, one can argue that comparing our work with electrical/thermal or antenna-coupled or broadband or absolute power detectors is not fair, because the compared detectors do not have exactly the same features and operation principles, and their performance has different dependencies on bandwidth, integration time, power, etc.

As part of our manuscript revisions, Reviewer 1 found the comparison with other direct detectors "invalid" and asked us to compare our work with detectors that include mixers with an electrical or optical local oscillator, and in particular suggested comparing our work with Ref. 35 of the revised manuscript. Quoting from the reviewer:

"Although it is claimed that the detector surpasses state-of-the-art detectors, the provided comparison is invalid for multiple reasons: The comparison was only made to direct detectors. The presented receiver is a multiplicative mixer that uses an optical local oscillator (LO), but it is compared to direct detectors, which scale differently with respect to bandwidth. A more accurate comparison would include mixers with an electrical or optical local oscillator, such as Schottky diode-based subharmonic mixers or photoconductive mixers. Such mixers, as described in the literature, provide far superior sensitivity than that demonstrated here. [3], [4]."

Based on the reviewer's comment, we revised our manuscript and modified our comparison with the detectors suggested by the reviewer (exactly the same reference that the reviewer suggested). Therefore, it is very unfair that the reviewer is now questioning the fairness of this comparison that he himself suggested:

The publications referenced in ref. 35 and ref. 36 use a standard lock-in scheme, and, as expected, sensitivity is independent of integration time. However, the authors do not properly explain why the setup in reference 7 is incomparable to theirs.

The results in this work were obtained using the evaluation scheme 'spectral lock-in' which differs significantly from the evaluation methods used in the other cited publications. The different evaluation method produces different results, rendering a fair comparison impossible.

The authors obtain their sensitivity values differently than state-of-the-art publications. Consequently, their sensitivity appears to be orders of magnitude higher and thus beyond state of the art, which is an unsubstantiated claim.

Nevertheless, the provided results indicate that the measurement scheme cannot be used to compare the receiver to literature values obtained by different methods, even without detailed knowledge of the evaluation process.

[FIGURE REDACTED]

Here we provide some context about the detector/receiver type that the reviewer suggested we compare with, and which the reviewer now finds unfair. This is a detector/receiver used in a coherent homodyne system. The figure on the right shows the block diagram from Ref. 35. The same laser pair that produces the terahertz beat frequency is used to both generate and detect terahertz radiation. Because the optical beat frequencies are identical or offset by a fixed frequency difference determined by the optical path lengths, the receiver's detected electrical signal appears at a fixed frequency (DC or low-frequency baseband). Therefore, a standard lock-in detection method is used to extract the receiver output while reducing noise, but at the expense of losing the frequency components of the signal. Losing the frequency components is not a problem in such a coherent system since the detected frequency matches the lasers' optical beat frequency (with or without a fixed offset). Because the optical beat frequencies driving the terahertz transmitter and receiver are phase-coherent, the system is immune to laser drifts and variations—problems that negatively impact the operation of incoherent systems like the detector we presented in this manuscript. As a result, very narrowband filters can be used to significantly reduce the detected noise. Quoting from Ref. 35: "*A software lock-in amplifier that was locked to a 20 kHz reference was used to filter the sampled signal with a bandwidth of 0.5 Hz. An integration time of 300 ms per frequency point was used to further reduce the noise*." For comparison, in our manuscript, we use a filter bandwidth of 1.5 MHz to account for possible laser drifts, which significantly increases the noise that we pick up compared to Ref. 35,

which can use a 0.5 Hz filter without worrying about any laser drift while ensuring the generated and detected signals perfectly align in frequency, thanks to the coherence of the system. While such a coherent homodyne system benefits from phase coherence to achieve very low noise levels, its usage is limited to applications where the receiver is synchronized or locked with the transmitter, or when the transmitter and receiver share the same laser to drive them. Therefore, coherent homodyne systems are restricted in communication and remote sensing applications.

The detector presented in our manuscript does not have this constraint and operates completely independently of the source of terahertz radiation. However, since we do not have prior knowledge of the frequency of the signal we are detecting, we use a spectral lock-in detection technique to maintain the frequency components of the detected signal. Spectral lock-in detection is essentially the same as standard lock-in detection, with only one difference: the frequency components of the signal are first calculated, and standard lock-in detection is then applied to all frequency components in parallel. Please see the table below, where we summarize the similarities and differences between the detector presented in this manuscript and that in Ref. 35, which was recommended by the reviewer.

	Ref. 35 suggested by the reviewer	This manuscript
Detector type	Photomixer-based detector operating in a coherent homodyne scheme	Photomixer-based detector operating in a incoherent direct detection scheme
Optical beat frequency of the detector	Synchronized with the source of radiation (coming from the same laser)	Independent of the source of radiation
Relation to the detected radiation	The exact frequency of the detected radiation is known	Frequency of the detected radiation is unknown
Lock-in detection	Standard Lock-in detection	Spectral lock-in detection
Filter bandwidth restricting the noise power (proportional to noise power)	0.5 Hz	1.5 MHz
Noise power reduction factor as a function of integration time	10 dB/dec	10 dB/dec
Signal power reduction factor as a function of integration time	10 dB/dec	Unchanged
Input referred noise reduction as a function of integration time (W/Hz)	Unchanged	10 dB/dec
Integration time	300 ms	64 ms

Back to the benchmarking discussion, comparing our detector with coherent homodyne systems (recommended by the reviewer) is unfair in the sense that our detector does not benefit from the phase coherence of those systems, and we do not have the luxury of using very narrowband filtering (e.g., 0.5 Hz) to suppress noise. On the other hand, our detection scheme maintains the frequency information of the signal through spectral lock-in detection. Therefore, we do not suffer from signal power reduction when we increase the integration time, which is a limitation for coherent homodyne systems, as detailed above and noted in the table above.

In summary, benchmarking terahertz detectors is not straightforward, and no matter which category of detectors we compare with, one can find features that are not identical in the comparison. However, for the particular case of our manuscript, **we find it very unfair that we revised our manuscript based on the reviewer's comment and modified our comparison with the detectors suggested by the reviewer (exactly the same reference that the reviewer suggested), and that the reviewer is now questioning the fairness of the comparison he himself recommended.**

Furthermore, their previous publications with comparable methods but far better results are missing.

We have addressed this comment in our response to the reviewer in the previous round and explained that our previous work (Wang et al., DOI: 10.1038/s41550-019-0828-6) is not using comparable methods. It uses Y-factor measurements under different hot/cold blackbody sources to extract the system noise temperature, which is very different from the measurements performed in this manuscript and the suggested references by the reviewer. Quoting from our response to the reviewer in the previous round:

As described above, the setup, measurement scheme, and operating conditions used in this work differ significantly from those in Wang et al. Therefore, a direct comparison between our results and the Y-factor noise temperature measurements reported by Wang et al. is not straightforward and fair.

In addition, Wang et. al. describes a precision astronomy instrument that is developed under various stabilization measures that are not used in this manuscript. For further details, please see our response to the reviewer's comment about references [6] and [8].

Now, we address the other aspect of the reviewer's concerns about our demonstrated detector:

(2) Spectral lock-in detection method:
In the first round of reviews, the reviewer assumed that we used a radiometer measurement scheme. In our response letter, we clarified that the reviewer misunderstood our method—we did not use a radiometer method; instead, we used a spectral lock-in detection method. Unfortunately, the reviewer still keeps stating that we used a radiometer measurement scheme and, based on this wrong assumption, concludes that the achieved 10 dB/decade reduction in noise is not plausible and suggests a significant flaw in our signal processing. **<u>WE HAVE NOT USED A RADIOMETER METHOD.</u>**

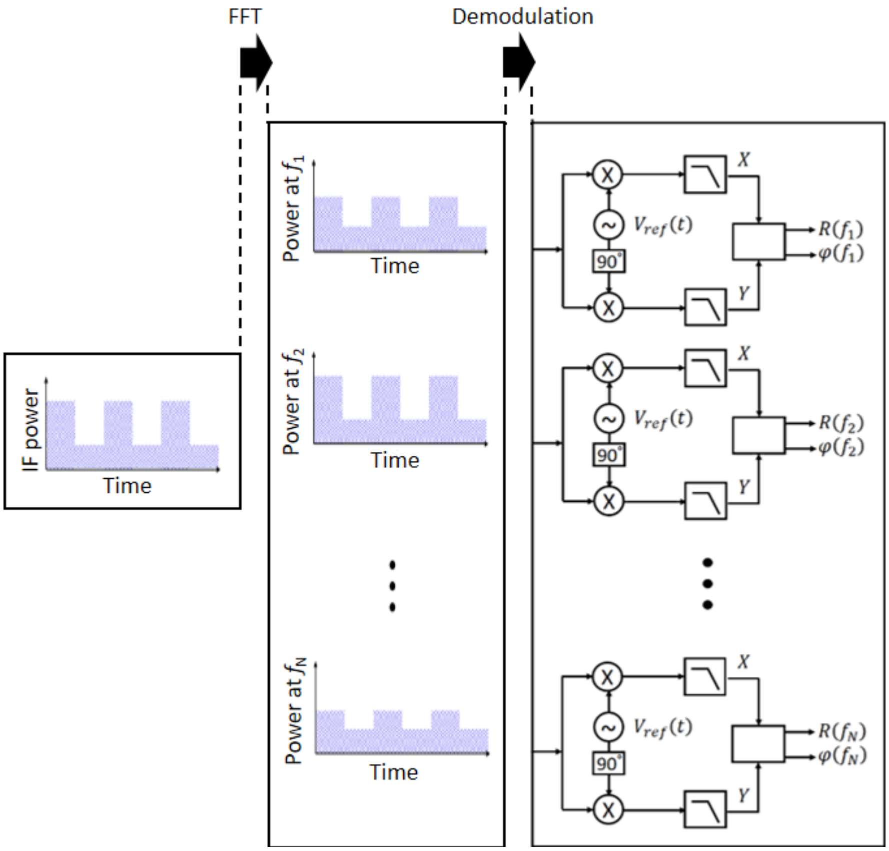

In the revised manuscript in the previous round, we added the following explanation of how data analysis using spectral lock-in detection is performed:

To perform spectral lock-in detection, the input terahertz signal is first modulated, and the electrical spectrum analyzer is replaced with a real-time lock-in detection analyzer. This analyzer down-converts the IF spectrum from ~0.8 GHz to ~40 MHz using an RF mixer (RFCOMP HD26217) and then digitizes it with an analog-to-digital converter (Texas Instruments ADS5485). A Fast Fourier Transform is then applied to the time-domain signal, decomposing it into distinct frequency components spanning 0–100 MHz. All frequency components are demodulated in parallel to generate the lock-in detected spectrum.

The block diagram above illustrates the spectral lock-in detection process. Please note that the described spectral lock-in detection method is no different from standard lock-in detection, with only one difference: the frequency components of the signal are separated first, and then the standard lock-in detection process is applied to every frequency component. Therefore, while we benefit from all the advantages of a standard lock-in detection process (e.g., noise reduction), we can maintain the frequency information of the signal. The remaining parts (e.g., I/Q demodulation and filtering) are exactly like standard lock-in detection methods.

We have added a recent conference paper citation (Ref. 48) to the revised manuscript, which provides experimental demonstration of the spectral lock-in detection process and a detailed description of the method, including the illustrated block diagram (Hwang, J. J., Tsao, S.A. & Jarrahi, M. An Ultra-Low-Noise 600-700 GHz Heterodyne Terahertz Receiver for Ground-Based Astronomy Observations. IEEE/MTT-S International Microwave Symposium Digest, 134-137 (2025)).

As experimentally demonstrated in this paper, while the noise power is reduced at a rate of 10 dB/decade as a function of integration time (as in the case of the standard lock-in detection method), the signal power remains unchanged since this technique inherently maintains all the frequency information. As a result, the detection sensitivity is improved at a rate of 10 dB/decade as a function of integration time. This is very different from the trend in Ref. 35 and Ref. 36, which demonstrate coherent homodyne terahertz systems with standard lock-in detection. In those systems, the noise power is reduced at a rate of 10 dB/decade as a function of integration time; however, the signal power is also reduced at a rate of 10 dB/decade as a function of integration time. For this reason, although the noise power is reduced as a function of integration time, the sensitivity (W/Hz) does not change as a function of integration time. Based on the above explanation and the experimental demonstration in Ref. 48, the reviewer's statement that "*the sensitivity of a receiver is a fixed value and therefore should not scale with integration time*" is not true for all types of receivers. It is applicable to the specific coherent homodyne systems demonstrated in Ref. 35 and Ref. 36, but not to the detector demonstrated in our manuscript. Similarly, the reviewer's statement that "*input-referred noise reduction with integration time is only possible with radiometers, which would result in a 5 dB/decade reduction*" is incorrect, as clearly demonstrated in Ref. 48.

The following explanations are intended to provide background information to help you understand the nature of my concerns.

1. Lock-In Technique vs. Radiometer
The authors appear to use a radiometer measurement scheme to determine the sensitivity value of their receiver, whereas the references use a lock-in technique. The following will explain the main differences between the two approaches and why the results differ. The radiometer scheme is common in astronomy and weather/atmosphere measurements because it surpasses actual receiver sensitivity by orders of magnitude. This is done by comparing noise power and noise plus signal power. The process involves modulating the source and then comparing the off and on states. Essentially, it averages the noise power based on the off state in a fixed bandwidth and subtracts this value from the on-state signal. This subtraction scales with integration time because the standard deviation of the mean values scales with the square root of N, or in this case, the square root of τ. This explains why input-referred noise (sensitivity) scales with integration time. However, this is not an inherent noise value; it is the standard deviation of the noise. Consequently, using those sensitivity values without first calculating the original noise using the radiometer equation renders them completely incomparable to regular measurements. With the 1.5 MHz bandwidth and 64 ms integration time used here, the smallest detectable power appears ~300 lower than the actual receiver sensitivity. Unlike a radiometer, the standard lock-in scheme is essentially a bandpass filter with I/Q demodulation. It does not involve power averaging, and the integration time is inversely proportional to the bandwidth. Therefore, integrating for a longer time reduces the bandwidth, allowing one to distinguish a narrowband signal from the noise. With ten times the integration time, the bandwidth and integrated noise power are both reduced by a factor of ten. However, the input-referred noise power spectral density (sensitivity) in W/Hz remains constant as expected.

As stated above, **<u>WE HAVE NOT USED A RADIOMETER METHOD.</u>** Therefore, the comparisons mentioned above are not applicable to our manuscript. On the previous page, we described that we used a spectral lock-in detection method, which is no different from standard lock-in detection, with only one difference: the frequency components of the signal are separated first, and then the standard lock-in detection process is applied to every frequency component. Therefore, while we benefit from all the advantages of a standard lock-in detection process (e.g., noise reduction), we can also maintain the frequency information of the signal. The remaining parts (e.g., I/Q demodulation and filtering) are exactly the same as in standard lock-in detection methods.

2. 'Spectral Lock-in' Scheme and Receiver sensitivity
In the rebuttal, the authors write that refs. 7 and 8 also use a lock-in technique. However, they overlook the fact that their own measurement scheme is still quite different. They refer to their measurement scheme as "spectral lock-in" and claim a 10 dB/decade improvement in noise and signal-to-noise ratio (SNR). However, input-referred noise reduction with integration time is only possible with radiometers, which would result in a 5 dB/decade reduction, as seen in the original manuscript. Furthermore, their scheme includes a fixed power integration bandwidth of 1.5 MHz and a modulated signal source, similar to a radiometer setup. In this

configuration, achieving a 10 dB/decade reduction in noise is not plausible and suggests a significant flaw in the signal processing. Therefore, the measurement results are highly questionable.

Based on the above explanations and the experimental demonstration in Ref. 48, the reviewer's statements that '*10 dB/decade reduction in noise is not plausible and suggests a significant flaw in the signal processing*' and '*input-referred noise reduction with integration time is only possible with radiometers, which would result in a 5 dB/decade reduction*' are incorrect. Also, we repeat again that **WE HAVE NOT USED A RADIOMETER METHOD.**

The authors neither explain the measurement scheme in detail nor cite the publication in which they use the term "spectral lock-in." [6]. In order to fully understand this measurement scheme, the authors must provide:
(a) A detailed block diagram and description explaining the entire signal chain and processing.
(b) Measurements with different source input powers to verify the linearity of the measurement scheme.
c) Show the phase information of the spectra in Fig. S6c.

In the revised manuscript from the previous round, we added the following explanation of how spectral lock-in detection is performed:

To perform spectral lock-in detection, the input terahertz signal is first modulated, and the electrical spectrum analyzer is replaced with a real-time lock-in detection analyzer. This analyzer down-converts the IF spectrum from ~0.8 GHz to ~40 MHz using an RF mixer (RFCOMP HD26217) and then digitizes it with an analog-to-digital converter (Texas Instruments ADS5485). A Fast Fourier Transform is then applied to the time-domain signal, decomposing it into distinct frequency components spanning 0–100 MHz. All frequency components are demodulated in parallel to generate the lock-in detected spectrum.

We have also added a recent conference paper citation (Ref. 48) to the revised manuscript, which provides a detailed block diagram and description explaining the entire signal chain and processing, as well as an experimental demonstration of the spectral lock-in detection process (showing a 10 dB/decade sensitivity enhancement as a function of integration time). Our experimental results also include measurements with different source input powers to verify the linearity of the measurement scheme over six decades (Fig. 4b of Ref. 48). In addition, we added the phase information of the spectra as a function of integration time to the inset of Fig. S6c.

Furthermore, in references [6] and [8], the authors appear to use the same "spectral lock-in" scheme as in this manuscript while reporting sensitivity orders of magnitude better (2.5 K noise temperature in reference [8] is equivalent to 3.45×10^(−8) fW/Hz).

The work mentioned by the reviewer is a receiver developed for precision astronomy observations. It is a setup that includes optical alignment stabilization, optical intensity stabilization, and frequency stabilization (PLL) modules, and operates under long integration times—none of which are used in the detector presented in this manuscript. As described in Ref. 48, the 2.5 K noise temperature was achieved over a 320-second integration time, which is 5000 times longer than the 64 ms integration time used for the longest measurement in Fig. S6c. Since our detector sensitivity is enhanced at a rate of 10 dB/decade as a function of integration time, this 5000× increase in integration time translates to a <u>5000× increase in sensitivity.</u> Furthermore, the 2.5 K noise temperature in Ref. 48 was achieved using a PLL that significantly narrows the optical pump linewidth, enabling spectral lock-in detection with a 125 kHz bandwidth—12 times smaller than the 1.5 MHz bandwidth used in our experiments, which do not utilize a PLL. This narrower bandwidth provides a 12× reduction in noise power while maintaining the same signal power through spectral lock-in detection, resulting in <u>another 12× enhancement in sensitivity</u>. In addition, the optical alignment stabilization and optical intensity stabilization used to achieve the 2.5 K noise temperature each contribute 1–2 orders of magnitude reduction in the standard deviation of the detected signal over a 64 ms integration time. Therefore, depending on the operating environment (e.g., temperature fluctuations, laser current fluctuations, vibrations, etc.), the optical alignment and intensity stabilization techniques used in Ref. 48 could provide <u>an additional sensitivity enhancement of 2–4 orders of magnitude</u> compared to the detector demonstrated in our manuscript, which does not incorporate any of these features.

There are further indications that the scheme used in the manuscript produces inconsistent results:
a) Conversion loss and input-referred noise vary between manuscripts and methods: As previously mentioned, the relationship between sensitivity and conversion loss should remain consistent. However, this relationship changes from ~1 fW/Hz @ 45 dB in the first manuscript to ~0.02 fW/Hz @ 45 dB in the revised manuscript, which is a mismatch of more than one order of magnitude. Neither value matches the theory, and the authors do not explain why the values and their relation changed so much. The only reasonable conclusion is that both measurement schemes largely distort the results. If these measurement schemes provided reliable sensitivity values, they would match.

We indeed explained why the sensitivity values changed from our original manuscript to the revised manuscript. Quoting from our response to the reviewer in the previous round:

In the original manuscript, our spectral lock-in detection code applied lock-in detection to the spectral power data, yielding a 5 dB/dec noise power reduction rate with integration time. In the revised manuscript, we repeated the detector measurements using a modified spectral lock-in detection code that applies lock-in detection to the spectral voltage data (consistent with refs. 7 and 8, as suggested by the reviewer), which results in a 10 dB/dec noise power reduction rate with integration time. Consequently, the revised manuscript shows lower input-referred noise levels, as illustrated in the updated Fig. 2h.

Here, we present the calculations for the exact example that the reviewer gave:

	Original manuscript	Revised manuscript
Employed technique	Spectral lock-in detection applied to spectral power data	Spectral lock-in detection applied to spectral voltage (current) data
Noise power reduction factor (equivalent to sensitivity enhancement factor)	5 dB/dec	10 dB/dec
Integration time	25 ms	64 ms
Input referred noise for the first data frame captured over 64 us (no integration applied)	20 fW/Hz	20 fW/Hz
Input referred noise after integration based on the sensitivity enhancement factor (5 or 10 dB/dec)	20 fW/Hz / (25ms/64us)^0.5 = **1 fW/Hz**<br>For 5 dB/dec	20 fW/Hz / (64ms/64us) = **0.02 fW/Hz**<br>For 10 dB/dec

As calculated above, the experimentally achieved sensitivity values perfectly match the theoretical predictions for the detector demonstrated in our manuscript, and these statements by the reviewer are incorrect: "*The only reasonable conclusion is that both measurement schemes largely distort the results. If these measurement schemes provided reliable sensitivity values, they would match*." and "*Neither value matches the theory*."

b) Unclear choice of integration times and bandwidths: As mentioned above, the measurement schemes produce very different and implausible results. It is unclear why the integration time was 25 ms in the initial manuscript and 64 ms in the revised one. This is confusing, given that the inset in Fig. 2h indicates that they measured both in a region from 0.1 ms to 100 ms. Furthermore, if the input-referenced noise is not constant, but rather scales by 10 dB/decade or 5 dB/decade, how can one select a plausible integration time and bandwidth for the comparison? If both manuscripts used the same integration time, the discrepancy explained in (b) above would be more obvious.

There is nothing special about the choice of integration time. Indeed, we have shown spectral lock-in detection results with integration times ranging from 64 μs to 64 ms in Fig. S6c, clearly illustrating how the results behave across the entire range (including at 25 ms). Also, the input-referred noise values in the inset of Fig. 2h are consistent with the noise measurement results shown in Fig. S6b over an integration time range of 64 μs to 1 s (not 0.1 ms to 100 ms). The choice of bandwidth is determined by the bandwidth of the signal we plan to detect.

Furthermore, based on our response to the reviewer's previous (similar) comments, the reviewer's statement that "*the measurement schemes produce implausible results*" is incorrect. We have shown the exact

calculations for the specific case highlighted by the reviewer, invalidating this statement and demonstrating that our experimental results are in agreement with the theoretical predictions of device operation.

c) Integration Time and Sensitivity: This is just for completeness, since it was mentioned earlier. The sensitivity of a receiver does not increase with longer integration times.

As described in our earlier response, the reviewer's statement that "*the sensitivity of a receiver does not increase with longer integration times*" is not applicable to all types of detectors (including the detector demonstrated in our manuscript); it is applicable to the coherent homodyne systems presented in Ref. 35 and Ref. 36.

In conclusion, a common method for measuring receiver sensitivity must be used to allow for a fair comparison to the state of the art. The following procedure can be used: First, measure the receiver output noise with an ADC. Then, calculate the power spectral density without subtracting anything. Finally, refer this spectral power density to the input with the conversion loss. This can be done with the measured data, so it should be possible with minimal effort.

We have used exactly the same procedure outlined by the reviewer. The receiver output noise (i.e., the receiver output in the absence of an input signal) is measured using an ADC, and the results are shown in Fig. S6b. The spectral power density values in the presence of an input signal are measured and calculated using spectral lock-in detection without any subtraction, as shown in Fig. S6c. Finally, the input-referred noise (sensitivity) values are calculated from the spectral power density and conversion loss, as shown in Fig. S6f.

Nevertheless, the provided results indicate that the measurement scheme cannot be used to compare the receiver to literature values obtained by different methods, even without detailed knowledge of the evaluation process.

As stated above, our measurement procedure for sensitivity is exactly the same as that used for other detectors in the literature, and the reviewer's comment that "*the measurement scheme cannot be used to compare the receiver to literature values obtained by different methods*" is incorrect.

To summarize, our sensitivity measurement method, based on the spectral lock-in detection technique (not a radiometer method), is technically correct and provides experimental results that are in full agreement with the theoretical predictions.

Introduction
As a motivation for this work, the authors claim that '…Previous work on waveguide-coupled terahertz sources and detectors has lacked compatibility with photonic integrated circuits (PICs)…'(l. 56-58) while both, emitters and receivers, are already shown to be compatible: Ref. 21 [1] of the manuscript demonstrates integration of the source. Additionally, there are publications showing that photodiodes can also be used as detectors [2]. Furthermore, waveguide-integrated photoconductors are demonstrated, which are suitable for photonic integration [3].

We have already addressed this comment in the following sentence in our manuscript. Here is the complete statement from the Introduction that addresses the reviewer's comment:

Previous work on waveguide-coupled terahertz sources and detectors has lacked compatibility with photonic integrated circuits (PICs) that incorporate both passive and active optical components [12-20]. Meanwhile, demonstrated integrated terahertz optoelectronics have relied on complex fabrication processes, such as multiple epitaxial growth steps [21] to achieve the monolithic integration of uni-traveling-carrier photodiodes with optical pump lasers, semiconductor optical amplifiers (SOAs), and modulators, as well as bonding multiple III-V semiconductor stacks onto a silicon photonics chip [22] to heterogeneously integrate lasers, modulators, and photodiodes. Beyond scalability issues, these integrated terahertz optoelectronics schemes have been limited to generating signals up to ~100 GHz without demonstrating high-frequency signal detection.

Please note these specific points about the references mentioned by the reviewer:

[1], [2]: As stated above, Ref. 21 [1] and [2] use uni-traveling-carrier photodiodes that are not compatible with standard photonic integrated circuits (PICs) offered by foundries. Ref. 21 [1] used a fabrication process that required three epitaxial growth steps. Quoting from the authors of Ref. 21 [1] and [5]: "*The main drawbacks of this approach are that it requires a dedicated fabrication process flow*." The fabrication of the terahertz sources and detectors demonstrated in our manuscript does not require multiple epitaxial growth steps and is fully compatible with standard PIC foundry processes.

[2], [3]: These references do not demonstrate photodiodes integrated with photonic integrated circuits (PICs) that incorporate both passive and active optical components.

Terahertz emitter
The discussion of the SOA integration is noted, and I agree with the authors that the 'gain enhancement' is an interesting feature. However, this is also possible based on already available technologies like photonic integration platforms that provide building blocks for PD and SOA. Consequently other platforms already demonstrated the so-called 'gain-enhanced 'terahertz generation by integrating an SOA with a PD [4], [5].
In conclusion, combining SOAs and photodiodes for terahertz generation is possible on other platforms and has been demonstrated.

As stated above and explained in our manuscript, uni-traveling-carrier photodiodes are not compatible with standard photonic integrated circuits (PICs) offered by foundries that include PDKs for SOAs, lasers, and passive components, and their fabrication process requires multiple epitaxial growth steps. Quoting from the authors of [1], [4], [5]: "*The main drawbacks of this approach are that it requires a dedicated fabrication process flow.*" In contrast, the fabrication of the terahertz sources and detectors demonstrated in our manuscript does not require multiple epitaxial growth steps and is compatible with standard PIC foundry processes.

The claimed record efficiency value is largely defined by SOA gain. A fair comparison to the state of the art would require include the efficiency of the PD itself. Furthermore, the claim of '…significant improvements in power efficiency … over state of the art' (line 14) cannot be made, as other publications on gain-enhanced THz emitters did not consider this figure of merit.

As mentioned in our previous response to the reviewer, monolithic integration with other active/passive optoelectronic components on the same chip is the unique advantage of our devices. This includes optical gain on the same chip without the need to use an additional bulky amplifier that have been used for other reported work. To address the reviewer's comment and better clarify the impact of the on-chip gain, we have added a discussion on how SOA gain contributes to the enhancement of terahertz generation efficiency when discussing the comparisons in Figure 3 and added the following statement:

Our estimates indicate that the SOA gain increases the terahertz generation efficiency of the demonstrated sources by an order of magnitude (see Supplementary Fig. S7).

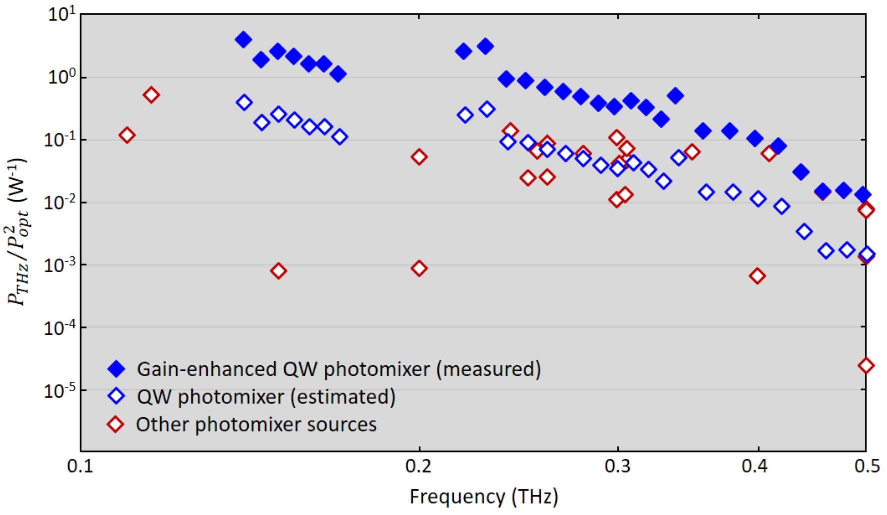

Fig. S7. *The impact of the SOA gain on the terahertz generation efficiency is evaluated by comparing the efficiency figure of merit P_{THz}/P^2_{opt} at the measured input optical power to the SOA (5 mW) and the estimated input optical power to the photomixer. The estimated input optical power to the photomixer is calculated as $\frac{1}{\eta}\frac{hc}{q\lambda}I_{ph} = 16.3$ mW, where h is the Planck's constant, c is the speed of light, q is the electron charge, λ is the optical wavelength of 809 nm, η is the photomixer quantum efficiency, estimated to be 56.5% from the*

electromagnetic simulations (Fig. 2b), and I_{ph} = 6 mA is the photomixer photocurrent. Based on these estimates, the SOA gain is found to enhance the terahertz generation efficiency by approximately an order of magnitude.

Previously Published Plots and Graphs
Many of the key graphs were included in publications made before or during the review process of this manuscript (see attachment for an overview). While a comprehensive publication of all the results in one place is valuable, it could potentially conflict with some journal guidelines. I will leave it to the editor to evaluate this.

We appreciate the reviewer's effort in locating our SPIE conference presentation videos online, capturing snapshots of the graphs from the slides, and compiling the attached table of figures. We would like to clarify that the SPIE presentations were based solely on submitted abstracts and are not associated with any published proceedings.

As encouraged by Nature/Springer group, some of the results in this manuscript were previously presented at other conferences and have been published in the following proceedings, which we have cited in the revised manuscript.

- Zhao, Y., Zumrat, S. E. & Jarrahi, M. Monolithically Integrated Optoelectronic Terahertz Sources and Detectors on Quantum Well PIN Substrates. IEEE/MTT-S International Microwave Symposium Digest, 134-137 (2025).
- Zhao, Y., Zumrat, S. E. & Jarrahi, M. GaAs/AlGaAs Quantum Well Photomixers for High-Efficiency Terahertz Generation and Detection. Proc. Conference of Lasers and Electro-Optics, 1-2 (2025).
- Zhao, Y., Zumrat, S. E. & Jarrahi, M. Monolithically Integrated Terahertz Optoelectronics Based On Quantum Well Structures. Proc. International Conference on Infrared, Millimeter, and Terahertz Waves, 1-2 (2024).

However, this manuscript provides a substantial extension of the results, methodology, analysis, conclusions, and implications beyond those presented in the conference proceedings. We believe this is in line with the policies of Nature Communications. Quoting from https://www.nature.com/nature-portfolio/editorial-policies/preprints-and-conference-proceedings:

Publishing work in conference proceedings is common in some research communities. The Nature journals are happy to consider submissions containing material that has been published in a conference proceedings paper. However, the submission should provide a substantial extension of results, methodology, analysis, conclusions and/or implications over the conference proceedings paper; the final decision on what constitutes a substantial extension is made by the editors at each individual journal.

Conclusion
I cannot recommend this manuscript for publication. My primary concern is the method used to evaluate receiver sensitivity, which seems questionable and differs fundamentally from other recent literature. This results in highly overestimated sensitivity values and a strongly misleading presentation. Furthermore, this method cannot be used to claim that sensitivity exceeds that of state-of-the-art receivers.

In the first round of reviews, the reviewer assumed that we used a radiometer measurement scheme. In our response letter from the previous round, we clarified that the reviewer had misunderstood our method—we did not use a radiometer method; instead, we used a spectral lock-in detection method. Unfortunately, the reviewer continues to state that we used a radiometer measurement scheme and, based on this incorrect assumption, concludes that our results present "*highly overestimated sensitivity values and a strongly misleading presentation*." In this response, we have addressed this misunderstanding once again and provided additional details that confirm the technical integrity of our method.

References
[1] G. Carpintero et al., "Microwave Photonic Integrated Circuits for Millimeter-Wave Wireless Communications," Journal of Lightwave Technology, vol. 32, no. 20, pp. 3495–3501, Oct. 2014, doi: 10.1109/JLT.2014.2321573.
[2] C. C. Renaud, M. Natrella, C. Graham, J. Seddon, F. Van Dijk, and A. J. Seeds, "Antenna Integrated THz

Uni-Traveling Carrier Photodiodes," IEEE Journal of Selected Topics in Quantum Electronics, vol. 24, no. 2, pp. 1–11, Mar. 2018, doi: 10.1109/JSTQE.2017.2725444.
[3] M. Deumer et al., "Waveguide-integrated photoconductive THz receivers," in 2022 47th International Conference on Infrared, Millimeter and Terahertz Waves (IRMMW-THz), Aug. 2022, pp. 1–2. doi: 10.1109/IRMMW-THz50927.2022.9895580.
[4] G. Carpintero et al., "Photonic-Enabled Terahertz Phase Arrays Using Dielectric Rod Waveguides for 6G Wireless Communications," in 2024 IEEE/MTT-S International Microwave Symposium - IMS 2024, June 2024, pp. 74–77. doi: 10.1109/IMS40175.2024.10600234.
[5] G. Carpintero, R. C. Guzmán, C. Gordón, G. Kervella, M. Chitoui, and F. Van Dijk, "Photonic Integrated Circuits for Radio-Frequency Signal Generation," Journal of Lightwave Technology, vol. 34, no. 2, pp. 508–515, Jan. 2016, doi: 10.1109/JLT.2015.2511040.
[6] S.-A. Tsao, J. Hwang, and M. Jarrahi, "Lock-in detection of terahertz spectra captured by plasmonic heterodyne terahertz receivers," in Terahertz, RF, Millimeter, and Submillimeter-Wave Technology and Applications XVIII, SPIE, Mar. 2025, p. PC1336508. doi: 10.1117/12.3046514.
[7] N. Wang, S. Cakmakyapan, Y. J. Lin, H. Javadi, and M. Jarrahi, "Room-temperature heterodyne terahertz detection with quantum-level sensitivity," Nature Astronomy 2019 3:11, vol. 3, no. 11, pp. 977–982, July 2019, doi: 10.1038/s41550-019-0828-6.
[8] J. Hwang, S.-A. Tsao, and M. Jarrahi, "Ultra-low-noise, room-temperature terahertz receiver based on plasmonic photomixing," in Terahertz, RF, Millimeter, and Submillimeter-Wave Technology and Applications XVIII, SPIE, Mar. 2025, p. PC1336502. doi: 10.1117/12.3047609.
[9] Y. Zhao, S.-E.- Zumrat, and M. Jarrahi, "Monolithically Integrated Terahertz Optoelectronics Based on Quantum Well Structures," in 2024 49th International Conference on Infrared, Millimeter, and Terahertz Waves (IRMMW-THz), Sept. 2024, pp. 1–2. doi: 10.1109/IRMMW-THz60956.2024.10697774.
[10] Y. Zhao and M. Jarrahi, "Monolithically integrated terahertz optoelectronics based on quantum well structures," in Terahertz Emitters, Receivers, and Applications XV, SPIE, Oct. 2024, p. PC1314104. doi: 10.1117/12.3028428.
[11] Y. Zhao, S.-E.- Zumrat, and M. Jarrahi, "Monolithically Integrated Optoelectronic Terahertz Sources and Detectors on Quantum Well PIN Substrates," in 2025 IEEE/MTT-S International Microwave Symposium - IMS 2025, June 2025, pp. 126–129. doi: 10.1109/IMS40360.2025.11103976.

Reviewer #2 (Remarks to the Author):

The authors answered most of my questions, clarified many things and put their achievements in more realistic and honest perspective. One thing I am still not completely satisfied with is the performance comparison: they relate the performance of their device to the unamplified input laser power, with the argument that the power after SOA amplification is not known and cannot be measured. But at least it must be possible to give an estimation (not to speak about fabricating a SOA test device and measure the amplified output). Is it a factor of 10? Or more likely 100? This uncertainty should also be stated where the performance is discussed (near Fig 2 and/or 3.)

We are glad that most of the reviewer's questions have been addressed and clarified. We agree with the reviewer that an estimate of the SOA amplification would help to better understand the role of amplification in the device performance. We have now added our estimate for the SOA gain, derived not from a fabricated SOA test device but from the specifications of the SOA-integrated photomixer. To address the reviewer's comment, we have included the estimated SOA gain (6.2 dB) in the manuscript and in the caption of Fig. 2. In addition, the details of this estimation have been added to the Methods section:

The SOA gain at an input optical power of 5 mW is estimated from the measured photocurrent of the photomixer. As illustrated in Fig. 2e, the highest terahertz power is achieved at a photomixer bias voltage of -3 V and a photocurrent of I_{ph} = 6 mA. Therefore, the estimated input optical power to the photomixer is calculated as $\frac{1}{\eta}\frac{hc}{q\lambda}I_{ph} = 16.3$ mW, where h is the Planck's constant, c is the speed of light, q is the electron charge, λ is the optical wavelength of 809 nm, and η is the photomixer quantum efficiency, estimated to be 56.5% from the electromagnetic simulations (Fig. 2b). The SOA output optical beam propagates through a 5-µm-long implanted electrical isolation region with an optical loss of 0.21 dB/µm (Fig. 2c) before reaching the photomixer, resulting in an estimated SOA output power of 20.76 mW and SOA gain of 6.2 dB.

We also discussed how SOA gain contributes to the enhancement of terahertz generation efficiency when discussing the comparisons in Figure 3 and added the following statement:

Our estimates indicate that the SOA gain increases the terahertz generation efficiency of the demonstrated sources by an order of magnitude (see Supplementary Fig. S7).

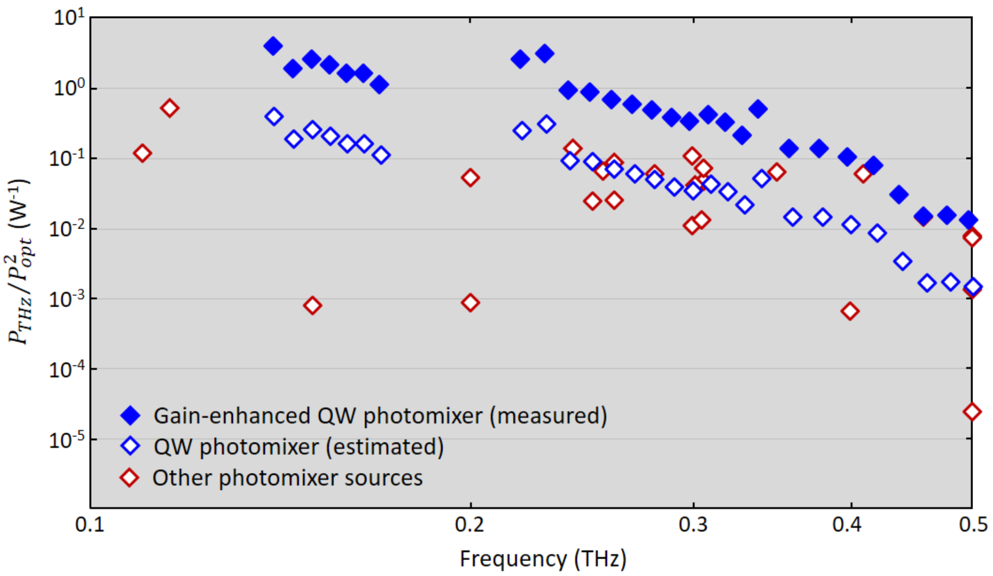

Fig. S7. *The impact of the SOA gain on the terahertz generation efficiency is evaluated by comparing the efficiency figure of merit P_{THz}/P_{opt}^2 at the measured input optical power to the SOA (5 mW) and the estimated input optical power to the photomixer. The estimated input optical power to the photomixer is calculated as $\frac{1}{\eta}\frac{hc}{q\lambda}I_{ph} = 16.3$ mW, where h is the Planck's constant, c is the speed of light, q is the electron charge, λ is the optical wavelength of 809 nm, η is the photomixer quantum efficiency, estimated to be 56.5% from the electromagnetic simulations (Fig. 2b), and I_{ph} = 6 mA is the photomixer photocurrent. Based on these estimates, the SOA gain is found to enhance the terahertz generation efficiency by approximately an order of magnitude.*

Reviewer #3 (Remarks to the Author):

The revisions have clarified the authors' claims, and the title has been updated accordingly. In my opinion, the work is on a rather specific terahertz technology and may not attract broad interest. Therefore, I believe it would be more suitable for a more specialized journal rather than Nature Communications.

We are glad that our response has clarified the manuscript's claims. In our view, the findings of our manuscript are very well suited for the highly interdisciplinary and broad readership of Nature Communications. Just as integrated circuit technology revolutionized early, bulky, power-hungry computers into the high-performance microprocessor chips in use today in homes, cars, phones, and health monitoring systems, the introduced monolithically integrated terahertz optoelectronics platform could bring about a similar technological transformation in the terahertz field. It has the potential to transform terahertz radar, communication, imaging, and sensing systems from expensive, bulky, laboratory-grade setups to scalable, compact, and low-cost microchips for everyday use.

My additional comments are as follows:

• While I can accept the inclusion of performance improvements achieved with the SOA, this result by itself does not appear to carry substantial scientific impact.

We agree with the reviewer that performance improvement achieved solely through the use of an SOA does not represent a substantial scientific impact. However, the contributions of our manuscript extend far beyond the incorporation of an SOA or mere performance enhancement. We demonstrate that high-performance terahertz optoelectronic sources and detectors can be monolithically integrated on the same QW PIN photodiode substrates provided by commercial PIC foundry processes. These processes already offer lasers, SOAs, modulators, filters, demultiplexers, and other passive optical components on the same chip; thereby enabling, for the first time, the monolithic integration of complex terahertz optoelectronic systems onto a single chip.

• The constraints imposed by the Kramers–Kronig relations apply when considering the full frequency response. In practice, however, devices are typically operated in a very narrow frequency window—for example, in a transparency region—where such effects are negligible and the device can essentially function as a pure phase modulator. In the present case, the absorption is significant, which raises concerns that the situation is quite different from that of a general modulator.

We agree with the reviewer that the absorption in QW PIN–based phase modulators is significant. Despite these high absorption levels, such phase modulators are widely employed in PIC processes because of their compatibility with lasers, SOAs, and passive components, enabling the realization of a complete optoelectronic system on a single chip. The optical loss introduced by these modulators is typically compensated by the gain provided by monolithically integrated SOAs available within these platforms.

The optical absorption in these phase modulators arises from the Quantum-Confined Stark Effect and is described by the Kramers–Kronig transform, which determines the changes in the refractive index (Δn) causing phase modulation as a function of bias modulation (ΔV) and wavelength (λ):

$$\Delta n(\lambda_0, \Delta V) = \frac{\lambda_0^2}{2\pi^2} P \int_0^\infty \frac{\Delta\alpha_{abs}(\lambda, \Delta V)}{\lambda_0^2 - \lambda^2} d\lambda$$

where $\Delta\alpha_{\mathrm{abs}}$ is the change in the optical absorption, λ_0 is the optical wavelength at which refractive index change is calculated, and *P* indicates the principle value of the integral. This equation illustrates the tradeoff between phase modulation (caused by the changes in Δn) and intensity modulation (caused by the changes in $\Delta\alpha_{\mathrm{abs}}$). It shows that larger variations in absorption ($\Delta\alpha_{\mathrm{abs}}$) near the operating wavelength lead to stronger phase modulation, independent of the signal bandwidth. This is the tradeoff faced by commercial PIC foundries when designing for efficient phase modulation while compensating optical attenuation through the available gain provided by monolithically integrated SOAs. In fact, the intensity–phase modulation tradeoff observed in our device exhibits specifications comparable to those of foundry-implemented modulators. As illustrated in

Figures 4b and 4c, for a 100-μm-long phase modulator, we achieve a Vπ of 0.5 V and an attenuation of 5.5 dB at Vπ, which is in the same range as the state of the art, including those provided by commercial foundries. These references are examples of such phase modulators which have optical losses ranging from 4-6 dB at Vπ.

- Gupta, Y.D., Binet, G., Diels, W., Abdeen, O., Gaertner, T., Baier, M. and Schell, M., 2023. Implementation, Modelling and Verification of High-Speed Mach-Zehnder Phase Modulators in an Open Access InP Foundry Platform. Journal of Lightwave Technology, 41(11), pp.3498-3504.

- Lange, S., Wolf, S., Lutz, J., Altenhain, L., Schmid, R., Kaiser, R., Schell, M., Koos, C. and Randel, S., 2018. 100 GBd intensity modulation and direct detection with an InP-based monolithic DFB laser Mach–Zehnder modulator. Journal of Lightwave Technology, 36(1), pp.97-102.

- Rouvalis, E., Metzger, C., Charpentier, A., Ayling, T., Schmid, S., Gruner, M., Hoffmann, D., Hamacher, M., Fiol, G. and Schell, M., 2014, September. A low insertion loss and low V π InP IQ modulator for advanced modulation formats. In *2014 The European Conference on Optical Communication (ECOC)* (pp. 1-3). IEEE.

- Juodawlkis, P.W., O'Donnell, F.J., Bailey, R.J., Plant, J.J., Ray, K.G., Oakley, D.C., Napoleone, A., Watts, M.R. and Betts, G.E., 2004, August. InGaAsP/InP quantum-well electrorefractive modulators with sub-volt Vpi. In Enabling Photonic Technologies for Aerospace Applications VI (Vol. 5435, pp. 53-63). SPIE.

Reviewer #1 (Remarks to the Author):

Thank you to the authors for revising the manuscript once again, and for providing such a detailed response. The SOA gain estimation and the additional data points in Fig. S7 now enable a fair comparison and eliminate the question of the SOA's influence. The emitter's efficiency appears to be very good, which is a remarkable result given the use of a multi-purpose epitaxial stack.

We are glad that the additional information helped address the reviewer's questions regarding the emitter performance. We thank the reviewer for acknowledging the remarkable performance of our emitter, which can be monolithically integrated with all other optoelectronic components of a terahertz system on a chip fabricated using standard PIC foundry processes.

Before addressing the reviewer's concerns about our detector, we briefly summarize the key principles relevant to interpreting our detection scheme that the reviewer has unfortunately overlooked, leading to incorrect assumptions that are repeated in his comments. These include:

(Point 1) The principles of spectral lock-in detection process → This information should correct the reviewer's incorrect assumption that we have used a radiometer method, leading to the erroneous conclusion that our sensitivity should be enhanced at a 5 dB/dec rate as a function of integration time. It also provides a detailed mathematical proof that the sensitivity of our detector is enhanced at a 10 dB/dec rate as a function of the integration time (backed by the experimental results in this manuscript and Ref. 48).

- As we have described in our response in the previous round of reviews, Ref. 48 of our manuscript provides a block diagram and description explaining the entire signal chain and processing, as well as an experimental demonstration of the spectral lock-in detection process, showing a 10 dB/decade sensitivity enhancement as a function of integration time. From the reviewer's comments, it does not appear that the reviewer has read our response or Ref. 48 to clarify his incorrect assumptions. To further clarify the specifics of our method, we have prepared Appendix A attached to this response letter. It describes the spectral lock-in detection process in further detail and mathematically proves that the spectral lock-in detection applied to the IF output of our detector, $v_s(t)$, results in a 10 dB/dec noise power reduction as a function of integration time while maintaining the signal power. As a result, the sensitivity is enhanced at a 10 dB/dec rate as a function of integration time. Stated differently, the input-referred noise power spectral density is reduced at a 10 dB/dec rate as a function of integration time. We will refer to this Appendix in our responses to the next comments of the reviewer, since most of the comments are the result of the same incorrect assumptions.

- In our latest response to the reviewer, we stated that our method does not correspond to a radiometer-based detection scheme; however, the reviewer continues to interpret our method as a radiometer-based scheme and concluding that our results are not valid because our noise reduction rate as a function of integration time differs from that of the radiometer method. As shown in the detailed description in Appendix A, the main difference between the radiometer method and our method is that we first perform spectral separation and demodulation directly on the sampled voltage (since we have a linear detector) and convert the lock-in-detected voltage to power in the subsequent steps. In contrast, the radiometer method directly applies the lock-in detection process to the measured power data through square-law detection, leading to a different sensitivity dependence on integration time.

- If the reviewer has difficulty understanding our method and confuses it with the radiometer technique, we suggest that the reviewer instead consider a terahertz time-domain spectroscopy (THz-TDS) system. A THz-TDS system can be regarded as a heterodyne system composed of many optical beat frequencies that collectively generate and detect a broadband signal in the frequency domain. From a signal-processing perspective, the spectral lock-in detection process used in our work is closely analogous to that of conventional THz-TDS systems. In particular, the lock-in detection process is applied to the detected output current or voltage,

resulting in a **noise-current spectral-density reduction that scales as 5 dB/dec** with increasing integration time, while the resolved **noise power spectral density scales as 10 dB/dec** with integration time, exactly like a THz-TDS system.

(Point 2) Dependence of input-referred noise spectral density on system/setup parameters → From the reviewer's comments, it seems that the reviewer believes the input-referred noise spectral density is an intrinsic characteristic of a photomixer-based receiver/detector and is independent of the setup or system used to operate it. We will clarify this incorrect assumption.

- We use the derivations in Ref. 39 (suggested by the reviewer), which formulate the estimated NEP (with units of W/Hz) based on the receiver parameters. These derivations result in an NEP formula (Eq. 12 of Ref. 39) that **appears to be** independent of the setup or system parameters and only a function of the receiver specifications, as shown in:

$$NEP_{THz}(f) = \frac{8k_B T}{\eta_{THz}^{eff}\eta_\tau(f)\eta_{RC}(f)} \cdot \frac{R_{PC}}{R_A(f)}. \qquad (12)$$

However, **important system parameters are hidden in this formulation, which are overlooked in the analytical derivations in Ref. 38 and 39**, making the NEP a strong function of the system parameters. Most notably the laser linewidth, drift, noise, and the detection scheme (i.e., coherent homodyne versus incoherent direct detection). To better understand this, please see Eq. 4 of Ref. 39, which shows the photocurrent formula used in Ref. 39 to find the receiver's responsivity to be independent of the system parameters:

$$I_r(f,t) = \frac{\eta_{opt}\eta_{bias}I_{id}\mu\tau_{rec}}{w_G^2} \cdot \left(1 + \cos(2\pi f t)\sqrt{\eta_\tau(f)\eta_{RC}(f)}\right) \cdot U_{THz}\cos(2\pi f t + \varphi). \qquad (4)$$

At a given laser beat frequency (terahertz frequency difference) and fixed power, the spectrum of the photocurrent is a strong function of the laser linewidth and drift, as illustrated in the cartoon below (in other words, I_{id} and U_{THz} in the above formula are strong functions of these system parameters):

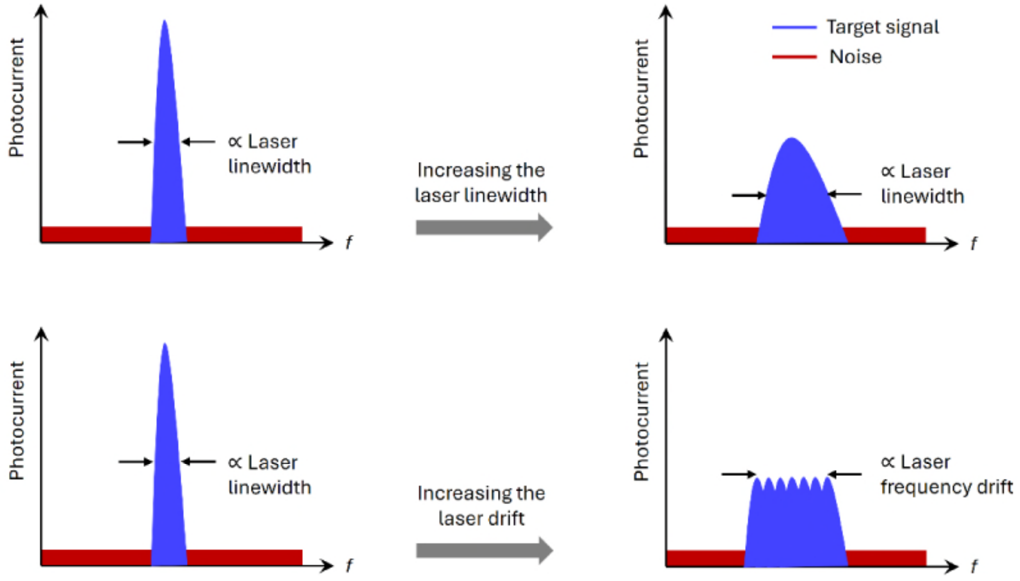

As illustrated in this cartoon, increasing the laser linewidth (at a fixed laser power) reduces the photocurrent spectral peak and widens the signal bandwidth, while maintaining the same noise spectral power density at a fixed optical power. Therefore, to maintain the same signal power at the output, the spectral power density must be integrated over a broader bandwidth, resulting in a higher total noise power. In summary, increasing the laser linewidth (while keeping all other parameters the same) increases the input-referred noise spectral power density of the receiver. Similarly, drift in the laser frequency reduces the photocurrent spectral peak and widens the signal bandwidth, while maintaining the same noise spectral power density at a fixed optical power. Therefore, increasing

the laser frequency drift (while keeping all other parameters the same) increases the input-referred noise spectral power density of the receiver.

Apart from laser linewidth and laser drift, the system architecture also has a significant impact on the input-referred noise spectral power density (i.e., sensitivity). For example, Refs. 38 and 39 suggested by the reviewer use a coherent homodyne architecture, which makes the system immune to laser frequency drift by ensuring that the same beat frequency is used for both generation and detection of the terahertz signal, even if the laser frequency drifts. In contrast, the detector presented in our manuscript uses an incoherent direct detection approach, in which no coherence or phase/frequency referencing is maintained between the detected terahertz frequency and the photomixer pump lasers (as explained in detail in our response in the previous rounds of review). Therefore, our results are strongly affected by laser drift. As clearly illustrated in our results in Fig. S6d, laser frequency drift in our setup reduces the peak spectral power density and widens the signal bandwidth, directly degrading the sensitivity. As described in the caption of Fig. S6, the input-referred noise power density of our detector is calculated as −14.5 dBm − SNR (dB) / 1.5 MHz, where −14.5 dBm is the input power and 1.5 MHz is the detection bandwidth. The use of a frequency-stabilized laser and/or a narrower laser linewidth result in a significant enhancement of the signal spectral peak (i.e., SNR) and allows the use of a much narrower detection bandwidth without losing signal power, leading to a significantly lower input-referred noise power density.

(Point 3) Dependence of input-referred noise spectral density of a receiver on integration time → While the reviewer keeps stating that the receiver's input-referred noise power spectral density must not scale with integration time as a "fundamental principle," this statement is not always valid and depends on many device and system parameters. Quoting the reviewer, "*According to the fundamental principle of receiver input-referred noise power spectral density, it must not scale with integration time*."

- The dependence of the input-referred noise power spectral density on integration time is determined not only by the detection scheme, but also by the system parameters (specifically the signal bandwidth). As an example, even the detectors presented in Refs. 38 and 39 (suggested by the reviewer) achieve improved input-referred noise power spectral density by increasing the integration time, as long as the signal bandwidth is smaller than 1/integration time per frequency point. In fact, Refs. 38 and 39 used lock-in detection with a 300 ms integration time, stating "*an integration time of 300 ms per frequency point was used to further reduce the noise*." The impact of integration time on lock-in detection and its relation with the signal bandwidth is clearly described in the textbook Meade, M. L., *Lock-in Amplifiers: Principles and Applications*, IEE Electrical Measurement Series, 1983, Section 2.4, and in Appendix A, where we describe a conventional lock-in detection process and prove the following points that the reviewer has overlooked: "*At short integration times, when the low-pass filter bandwidth is larger than the signal bandwidth, increasing the integration time reduces the noise power at a rate of 10 dB/decade with respect to integration time while maintaining the signal power, resulting in an SNR improvement of 10 dB/decade. However, at longer integration times, when the low-pass filter bandwidth becomes smaller than the signal bandwidth, both the noise power and the signal power decrease at a rate of 10 dB/decade, preventing any further enhancement in SNR with increasing integration time*."

- In summary, the specifications of a particular type of receiver/detector under specific operating conditions (e.g., signal bandwidth larger than 1/integration time) should not be treated as a "fundamental principle" for all other types of receivers or detectors under all operating conditions, unless the principle is proven to be universal. As detailed in Appendix A, we provide a mathematical proof that the sensitivity of our detector is enhanced at a 10 dB/dec rate with respect to integration time, which is also supported by our experimental results. Stated differently, our input-referred noise power spectral density is reduced at a 10 dB/dec rate with respect to integration time. Instead of invalidating these proven specifications based on a different trend observed for another type of detector in a particular operating regime, our results should be interpreted within the context of our specific system architecture.

(Point 4) Benchmarking approach → In our response to the reviewer in the previous round of review, we discussed benchmarking in detail. However, the reviewer is still overlooking important points about benchmarking. Therefore, here we elaborate on some of the points discussed in our previous response:

- Benchmarking terahertz detectors carries nuance, since there are several different types of detectors in the literature governed by different physical principles, resulting in different behaviors. Terahertz detectors can be divided into different categories: (A) coherent (referenced detectors) vs. incoherent (direct detectors); (B) electrical, optical, or thermal detectors; (C) broadband vs. narrowband detectors; (D) free-space-coupled vs. waveguide-coupled vs. on-chip-probed detectors; (E) active vs. passive detectors; and (F) electric-field vs. absolute-power detectors. Therefore, regardless of which category is chosen for comparison, one can question the fairness of the comparison because the detectors being compared do not have exactly the same features or operating principles.

- Since we use an incoherent direct terahertz detection architecture, in the original manuscript we compared our detector's performance with other incoherent direct terahertz detectors. These are detectors that are completely independent of the radiation source and do not require any coherence, synchronization, referencing, or locking between the radiation source and the detector. As part of our manuscript revisions, the reviewer found the comparison with other direct detectors "invalid" and asked us to compare our work with detectors that include mixers with an electrical or optical local oscillator, and in particular suggested comparing our work with Refs. 38 and 39, which use a coherent homodyne detection architecture. Based on the reviewer's comments, we revised our manuscript and modified our comparison to include the detectors suggested by the reviewer. At the same time, we cautioned the reviewer that this is not a fair comparison due to the fundamental differences between coherent homodyne and incoherent direct detection architectures. The details of these points can be found in our previous response letter; here is the summary we provided: "*Comparing our detector with coherent homodyne systems (recommended by the reviewer) is unfair in the sense that our detector does not benefit from the phase coherence of those systems, and we do not have the luxury of using very narrowband filtering (e.g., 0.5 Hz) to suppress noise. On the other hand, our detection scheme maintains the frequency information of the signal through spectral lock-in detection. Therefore, we do not suffer from signal power reduction when we increase the integration time, which is a limitation for coherent homodyne systems, as detailed above and noted in the table above. In summary, benchmarking terahertz detectors is not straightforward, and no matter which category of detectors we compare with, one can find features that are not identical in the comparison. However, for the particular case of our manuscript, we find it very unfair that we revised our manuscript based on the reviewer's comment and modified our comparison with the detectors suggested by the reviewer, and that the reviewer is now questioning the fairness of the comparison he himself recommended*."

  The reviewer is now asking for the "*input-referred noise without any temporal integration*" for comparison with Refs. 38 and 39, which is an unreasonable request for many reasons:

  (A) We do not understand what the reviewer is referring to when asking for the "*input-referred noise without any temporal integration*." This comment may stem from the reviewer's misunderstanding of our detection technique, assuming that we start with a measurement that provides unprocessed output power spectral density, which then goes through integration in later steps (as stated in one of the reviewer's comments). Unlike the reviewer's assumption, and as described in Appendix A, we start by sampling the detector output using an ADC over multiple time windows, and we need at least one time window to generate our first set of signal spectra. Stated differently, the very first signal spectrum is obtained from the collective data within a time window of duration T_{seg}, which cannot be zero. Regardless of the data acquisition and post-processing methods used in our work, generating a spectrum requires corresponding time-domain data over a nonzero time window. Therefore, we do not understand what the reviewer is referring to when asking for the "*input-referred noise without any temporal integration*."

(B) If the reviewer is asking us to avoid any lock-in detection when comparing our results with Refs. 38 and 39, this is an unfair request because Refs. 38 and 39 do use lock-in detection with an integration time of 300 ms, which is longer than the 64 ms integration time we used. While the reviewer does acknowledge this fact, he dismisses the contradiction by stating that the receiver's input-referred noise power spectral density must not scale with integration time. However, as we described in Point 3, this statement is not always valid, and even the detectors presented in Refs. 38 and 39 achieve improved input-referred noise power spectral density by increasing the integration time up to the point where the integration time becomes comparable to 1/signal bandwidth per frequency point. Quoting from these references "*An integration time of 300 ms per frequency point was used to further reduce the noise*."

(C) The reviewer disregards the fact that Refs. 38 and 39 already have a major advantage compared to our detector, in that they benefit from the phase coherence of the coherent homodyne setup—an advantage that is purely provided by their setup (not the receiver itself) and that significantly improves their input-referred noise spectral density, as described in Points 2 and 3. Despite the benefits that Refs. 38 and 39 gain purely from their setup, the reviewer argues that lock-in detection should be eliminated from our method because it is a feature of our setup. Quoting the reviewer, "*this scheme is not suited to determining the sensitivity of a detector itself, since it measures the sensitivity of the entire setup*." If the reviewer wishes to make this comparison fair, then our results should be compared with Refs. 38 and 39 under conditions in which (1) they use independent, free-running, unreferenced, and unlocked laser pairs for pumping the terahertz source and detector (not a coherent homodyne detection scheme) and (2) the same laser linewidth, noise and stability is used to characterize the detectors, (3) they do not perform any lock-in detection (unlike the current work that uses lock-in detection with a 300 ms integration time), which would definitely degrade the system performance significantly.

(D) As mentioned before, we use an incoherent direct terahertz detection scheme (not a coherent homodyne detection scheme) in our manuscript. For this reason, in the original manuscript we compared our detector's performance with other incoherent direct terahertz detectors in the literature. We would like to point out that all of those incoherent direct terahertz detectors in the literature use lock-in detection like our work. Therefore, we believe we have used a more fair comparison approach in our original manuscript when we compared our results with other incoherent direct terahertz detectors in the literature, all utilizing lock-in detection. As part of our manuscript revisions, the reviewer found the comparison with other direct detectors "invalid" and asked us to compare our work with Refs. 38 and 39, which use a coherent homodyne detection architecture and lock-in detection. Based on the reviewer's comments, we revised our manuscript and modified our comparison to include the detectors suggested by the reviewer. At the same time, we cautioned the reviewer that this is not a fair comparison due to the fundamental differences between coherent homodyne and incoherent direct detection architectures.

In summary, we find the reviewer's request unclear (as described in A), unfair, and misleading (as described in B, C, and D). We have clearly described the impact of integration time on the detection performance conceptually, experimentally, and mathematically, and the reader can clearly determine the input-referred noise spectral density of our detector as a function of integration time from Figs. 2h and S6.
To further clarify this point, we have added results obtained without applying lock-in detection to Fig. S6, where we show results corresponding to the captured data over a single time window of 8 μs.

In contrast, the method used to assess receiver performance remains essentially unchanged, meaning that the results cannot be compared to those in other publications.
There is no flaw in the way we assess our detector performance. Please see Points 1-4 for further justification.

This is clearly evident in the sensitivity of the receiver, as shown in Fig. 2h and Fig. S6f. According to the fundamental principle of receiver input-referred power spectral density, it must not scale with integration time.

As described in Point 1, Appendix A provides a mathematical proof that the sensitivity of our detector is enhanced at a 10 dB/dec rate with respect to the integration time, which is also supported by our experimental results. Also, as described in Point 3, the statement that "*the receiver's input-referred noise power spectral density must not scale with the integration time*" is not always valid and depends on many device/system parameters.

To support this, I provide an overview of the reasons why the sensitivity values given in the manuscript are invalid:
a) Fig. 2h and S6f clearly show scaling of input-referenced noise in W/Hz with integration time. However, this is fundamentally impossible; the power spectral density must not change with integration time.

As described in Point 1, Appendix A provides a mathematical proof that the sensitivity of our detector is enhanced at a 10 dB/dec rate with respect to integration time, which is also supported by our experimental results. Additionally, as described in Point 3, the statement that "*the receiver's input-referred noise power spectral density must not scale with integration time*" is not always valid and depends on many device and system parameters.

b) Relation between input-referred noise and conversion loss: The conversion loss of a receiver is fundamentally linked to its sensitivity and input-referred noise. Therefore, the fundamental limit of a receiver's sensitivity/input-referred noise can be calculated [1]. With -174 dBm/Hz thermal noise at room temperature (where the noise power is equal to the temperature multiplied by Boltzmann's constant) and 43 dB conversion loss (see Fig. 2h), the sensitivity of the receiver is, at best, -131 dBm/Hz (0.08 fW/Hz). However, Fig. 2h shows that the authors claim to have reached 0.02 fW/Hz, which would be approximately 6 dB better than the fundamental limit. In the inset, they even claim to have increased this by more than an order of magnitude with integration time.

Unfortunately, the reviewer is making two mistakes in his statement:

(1) The reviewer states that the *noise power is equal to the temperature multiplied by Boltzmann's constant*, which is incorrect. The spectral power density of noise is equal to the temperature multiplied by Boltzmann's constant.

(2) The reviewer is missing the fact that the spectral power density of noise is reduced at a 10 dB/dec rate as a function of integration time, as described in Point 1, and does not include this factor in his calculations. The 10 dB/dec reduction in the spectral power density of noise is both experimentally demonstrated in this manuscript (Figs. 2h and S6) and mathematically proved in Appendix A. Here, we provide a summary, and the details of the derivations can be found in Appendix A: As stated in Eq. 7, the variance of the sampled noise voltage over a time-window T_{seg} is $\sigma_0^2 = N_0/T_{seg}$ in each frequency bin, where N_0 is the spectral power density of noise (in units of V^2/Hz) and $1/T_{seg}$ is the bandwidth of the frequency bin. We prove that the mean signal power at each frequency bin after the spectral lock-in detection process includes the original signal power of $\frac{1}{2R}A_k^2$ plus a noise power proportional to $\frac{\sigma_0^2}{M}$, which is reduced as a function of integration time at a rate of 10 dB/dec (Eq. 21).

c) Phase information: In Fig. S6c, the authors have added an inset showing the phase information over integration time, as requested. The presented phase data supports the assumption that they are using only the magnitude in their lock-in scheme, as explained below. If they were acquiring the actual source signal phase, it would be random due to the heterodyne measurement principle; however, here it is constant/converging. This plot most likely represents the phase of the 15 kHz modulation rather than the source signal, indicating that the scheme used is related to a radiometer.

The reviewer is correct that we use the magnitude of the voltage (after sampling and separating the frequency components) in our spectral lock-in detection. However, this resemblance to the radiometer technique does not mean that we have used a radiometer method. As described in detail in Point 1 and Appendix A, our method is fundamentally different from the radiometer method, and unlike the radiometer method, which provides a 5 dB/dec reduction in noise power as a function of integration time, our method provides a 10 dB/dec reduction in noise as a function of integration time. As shown in the detailed description in Appendix A, the main difference

between the radiometer method and our method is that we first perform spectral separation and demodulation directly on the sampled voltage (since we have a linear detector) and convert the lock-in-detected voltage to power in the subsequent steps. In contrast, the radiometer method directly applies the lock-in detection process to the measured power data through square-law detection, leading to a different sensitivity dependence on integration time.

To resolve this issue, the authors must use input-referenced noise power density, based on unprocessed output power spectral density divided by conversion loss, for comparison purposes.

It is not clear what "issue" the reviewer is suggesting to resolve. As explained in Point 4, the reviewer appears to have a misunderstanding of our method and assumes that we start with a raw output power spectral density measurement before applying our spectral lock-in detection process. As described in Appendix A, we sample the detector output using an ADC and perform several signal-processing steps to extract the power spectral density for each time window. Therefore, our scheme does not have access to an **unprocessed** output power spectral density.

Below, I have provided comments on the author's response.
1) "Benchmarking and comparison with other detectors in the literature"
a) The authors argue that a comparison is not straightforward, stating that "Benchmarking is particularly tricky for terahertz detectors, since there are several different types of detectors…". I disagree. While there are certainly different types of detector with different bandwidths and coupling schemes, the sensitivity in W/Hz of mixer-based detectors with a local oscillator is a well-established figure of merit that enables receivers to be compared regardless of bandwidth or integration time. The same applies to noise temperature, which is a different representation of sensitivity and an equally universal figure of merit.

We respectfully disagree with the reviewer. As detailed in Point 2, the detection system (e.g., coherent homodyne vs. incoherent heterodyne), as well as the system parameters (e.g., laser linewidth, drift, noise) have a significant impact on the sensitivity of a photomixer-based detector in W/Hz. Also, as detailed in Point 3, the statement that "*the receiver's input-referred noise power spectral density must not scale with integration time*" is not always valid and depends on many device and system parameters.

b) The authors state: "Based on the reviewer's comment, we revised our manuscript and modified our comparison with the detectors suggested by the reviewer (exactly the same reference that the reviewer suggested). Therefore, it is very unfair that the reviewer is now questioning the fairness of this comparison that he himself suggested". I disagree. In my initial comment, I stated that there are multiple reasons why this comparison is invalid, including the measurement scheme. Resolving just one of these issues does not change the fact that the comparison is invalid.

We have addressed this comment in detail in Point 4.

c) The authors wrote: "We indeed explained why the sensitivity values changed from our original manuscript to the revised manuscript. Quoting from our response to the reviewer in the previous round:…". The authors explained the reduction in noise power. However, it remains unclear how the noise power scales with integration time when the signal power remains constant at a fixed resolution bandwidth of 1.5 MHz. Furthermore, as the noise is not reduced by narrowing the bandwidth as it would be with a regular lock-in, the reason for the difference in noise power reduction between the two schemes remains unclear.

A critical point that the reviewer appears to have overlooked is that spectral lock-in detection preserves spectral information while applying conventional lock-in detection independently to each frequency component. In this approach, the signal voltage in each frequency bin is a fixed (DC) value that is modulated and superimposed on background noise. As a result, the effective signal bandwidth of each frequency bin approaches an infinitesimally small value (i.e., a delta function). Consequently, the demodulated signal power at each frequency bin remains

constant, independent of the integration time. Because conventional lock-in detection is applied to the time stream of each individual frequency bin, the noise power of the lock-in–detected bins decreases at a rate of 10 dB/dec with increasing integration time. This leads to a corresponding signal-to-noise ratio (SNR) improvement of 10 dB/dec, while preserving frequency-domain information. A mathematical proof of this performance is provided in Appendix A, as discussed in Point 1, and is further supported by our experimental results.

d) In the rebuttal, the authors list the differences between the measurement schemes in Ref. 35 and their own manuscript. In this comparison, the setup used in the manuscript is referred to as a 'Photomixer-based detector operating in an incoherent direct detection scheme'. This implies that the integrated noise power would scale by 5 dB per decade. However, in the provided data, it scales with 10 dB per decade (integrated noise power in fW, with a constant power spectral density).

As described in Point 1, Appendix A provides a mathematical proof showing why the noise power is reduced at a 10 dB/dec rate with respect to integration time (not 5 dB/dec).

Furthermore, the scheme would only be considered a direct detection scheme if the authors used the magnitude of the FFT in their lock-in scheme instead of the complex value, as mentioned below. This would also mean that the setup would be equivalent to a radiometer, meaning that the presented values would be invalid for comparison purposes (in general, a direct detector with a chopped emitter and lock-in amplification would be equivalent to a radiometer).

The reviewer is correct that we use the magnitude of the voltage (after sampling and separating the frequency components) in our spectral lock-in detection. However, this resemblance to the radiometer technique does not mean that we have used a radiometer method. As described in detail in Point 1 and Appendix A, our method is different from the radiometer method, and unlike the radiometer method, which provides a 5 dB/dec reduction in noise power as a function of integration time, our method provides a 10 dB/dec reduction in noise as a function of integration time. As shown in the detailed description in Appendix A, the main difference between the radiometer method and our method is that we first perform spectral separation and demodulation directly on the sampled voltage (since we have a linear detector) and convert the lock-in-detected voltage to power in the subsequent steps. In contrast, the radiometer method directly applies the lock-in detection process to the measured power data through square-law detection, leading to a different sensitivity dependence on integration time.

In contrast, in Ref. 35, the emitter is not modulated and the integration is equivalent to a second filter stage. Thus, the noise is simply reduced by narrowing the bandwidth, and the sensitivity in fW/Hz can easily be calculated. More importantly, it stays constant over integration time.

As described in Appendix A, in order to perform lock-in detection, one needs to shift the signal frequency by f_{mod}, which can be accomplished in different ways. For example, this is achieved by modulating the emitter in our work and in Ref. 39. Ref. 38 (previously Ref. 35) accomplishes this by shifting the optical beat signal by f_{mod} and later using as the lock-in detection reference. Nevertheless, the principles of the lock-in detection process remain unchanged and are as described in Appendix A: "*At short integration times, when the low-pass filter bandwidth is larger than the signal bandwidth, increasing the integration time reduces the noise power at a rate of 10 dB/decade with respect to integration time while maintaining the signal power, resulting in an SNR improvement of 10 dB/decade. However, at longer integration times, when the low-pass filter bandwidth becomes smaller than the signal bandwidth, both the noise power and the signal power decrease at a rate of 10 dB/decade, preventing any further enhancement in SNR with increasing integration time*."

Therefore, if the sensitivity of the receiver in Ref. 38, under the operational and system specifications used in that reference (signal bandwidth larger than 1/integration time), remains constant over the integration time ranges considered, this does not suggest that any receiver's input-referred noise power spectral density must not scale with integration time in all other conditions (e.g., when the signal bandwidth is smaller than 1/integration time).

Therefore, there is no “fundamental principle” stating that the sensitivity should be constant over integration time under all detection schemes and system parameters, as also described in Point 3.

e) The authors state that their results are not comparable with those of their previous publication [2] because different measurement schemes were employed. However, noise temperature and sensitive/input-referred noise describe the same physical property and are directly convertible [1]. This raises the question of why the authors claim that these values depend on the measurement scheme. If this were true, any comparison with values from other sources would be invalid.

Quoting from our response to the reviewer in the previous round of reviews: “*Wang et. al. describes a precision astronomy instrument that is developed under various stabilization measures that are not used in this manuscript. For further details, please see our response to the reviewer’s comment about references [6] and [8].*”

Please see Table 1 in the following pages, which is included to respond to a similar comment from the reviewer. The table and the accompanying information describe how different setup specifications (e.g., different stabilization mechanisms) can result in sensitivity enhancement. Unfortunately, the reviewer treats the “receiver sensitivity” as an inherent property of the receiver, independent of the detection architecture and system specifications. As discussed in Points 2 and 3, the “receiver’s sensitivity” is not an isolated parameter independent of the detection architecture (e.g., coherent homodyne and incoherent direct detection) and system specifications (laser linewidth, drift, intensity fluctuations, noise, integration time relative to the signal bandwidth, etc.).

2) Spectral lock-in
a) In lock-in measurements, the signal-to-noise ratio (SNR) scales with integration time. This is because the integration acts as a band-pass filter that becomes narrower with increasing integration time. Consequently, the proportion of the spectrally constant noise power spectral density in W/Hz that falls within this filter band decreases as the integration time increases. However, this also means that, for heterodyne measurements on a broadband source (i.e. a source with a bandwidth greater than that of the lock-in filter), the narrowing of the lock-in filter bandpass with integration time will not increase the SNR. In this case, the sensitivity of the detector remains the fundamental limit of the minimum detectable power per bandwidth, which is independent of integration time.

We are glad to see the reviewer’s description of the lock-in detection process, which is in agreement with the principles described in Appendix A and Point 3. As the reviewer acknowledges, the sensitivity of the detector will not be enhanced by increasing the integration time if the signal bandwidth is greater than the lock-in filter (~1/integration time), but this is not true in all scenarios. For example, sensitivity will be enhanced at a 10 dB/dec rate as a function of integration time if the signal bandwidth is smaller than the lock-in filter (~1/integration time). Unfortunately, in his other comments the reviewer continues to state that the receiver’s input-referred noise power spectral density must not scale with integration time as a “fundamental principle,” without considering specific device and system parameters.

However, the authors modulate the source power and run the lock-in on this modulation. This makes it possible to measure power levels much lower than the receiver's sensitivity. This method is common in many fields since it enables very weak signals to be measured, almost independently of the receiver's sensitivity.

We would like to make the following clarifications and corrections before proceeding:

- We would like to remind the reviewer that Ref. 39 (suggested by the reviewer) also modulates the source power and performs lock-in detection on this modulation.

- The statement that source power modulation and lock-in detection enable the measurement of very weak signals almost independently of the receiver’s sensitivity is incorrect. Sensitivity of the receiver plays a significant role.

However, this scheme is not suited to determining the sensitivity of a detector itself, since it measures the sensitivity of the entire setup, including modulation of the source. Therefore, the extracted power levels do not represent the sensitivity in W/Hz as a fundamental property of the detector. Consequently, these values cannot be compared to sensitivity values provided in the literature.

Unfortunately, the reviewer treats “receiver sensitivity” as an inherent property of the receiver, independent of the detection architecture and system specifications. As discussed in Points 2 and 3, the “receiver’s sensitivity” of a photomixer-based receive is not an isolated parameter independent of the detection architecture (e.g., coherent homodyne and incoherent direct detection) and system specifications (laser linewidth, drift, noise, integration time relative to the signal bandwidth, etc.).

As described in Point 4, on one hand, the reviewer says that our results should be compared with Refs. 38 and 39, which use coherent homodyne detectors. On the other hand, the reviewer disregards the fact that Refs. 38 and 39 already have a major advantage compared to our detector, in that they benefit from the phase coherence of the coherent homodyne setup—an advantage that is purely provided by their setup (not the receiver itself) and that significantly improves their input-referred noise spectral density, as described in Points 2 and 3. Despite the benefits that Refs. 38 and 39 gain purely from their setup, the reviewer states that “this scheme is not suited to determining the sensitivity of a detector itself, since it measures the sensitivity of the entire setup.” If the reviewer wishes to make this comparison fair, then our results should be compared with Refs. 38 and 39 under conditions in which (1) they use independent, free-running, unreferenced, and unlocked laser pairs for pumping the terahertz source and detector (not a coherent homodyne detection scheme) and (2) the same laser linewidth, noise and stability is used to characterize the detectors, (3) they do not perform any lock-in detection (unlike the current work that uses lock-in detection with a 300 ms integration time), which would definitely degrade the system performance significantly.

A detailed block diagram would certainly help with understanding the measurement scheme. My understanding of the scheme is as follows: Assuming the signal source produces a signal at 100.1 GHz: This source is switched on and off at a frequency of 15 kHz, as mentioned in the manuscript. The receiver is then driven by an optical beatnote at ~100 GHz, which is down-converted to an intermediate frequency (IF) of ~100 MHz. In the sampled data, we therefore see alternating short noise periods and periods with an IF signal at ~100 MHz. A short-time FFT with 1.5 MHz bins is then applied, which is equivalent to filtering with many 1.5 MHz bandpass filters. For simplicity, only the FFT bin/bandpass around 100 MHz is considered. In the source-on state, the complex amplitude within this FFT bin will contain the IF (source) signal. However, due to the free-running lasers, the phase will be uncorrelated, random and will vary rapidly over time. In the source-off state, there will only be noise. When a lock-in amplifier is applied to this complex-valued signal, both the noise and the source signal will integrate to zero due to the uncorrelated phase. Thus, there will be no output signal to monitor. Nevertheless, the authors report signal and noise reduction over time. This can only be achieved by applying the lock-in scheme to the IF magnitude rather than the complex signal. In this case, the off-state integrates to the average noise magnitude and the on-state integrates to the average source signal magnitude, i.e. the noise power and signal power. The result of the lock-in is therefore the change in power between the noise and the signal, just like a radiometer. Because the variance of the average noise power scales with the square root of the number of samples, the noise reduction is 5 dB per decade. This is a digital implementation of a 'multichannel filter spectrometer' [3]. In conclusion, this scheme either measures no signal at all when a complex signal is used, or it measures power as a radiometer would when magnitude is considered. However, it should never reduce noise power by 10 dB per decade.

A more detailed block diagram is included in Appendix A, where we analytically describe our spectral lock-in detection and formulate the signal processing, proving that the noise power is reduced at a 10 dB/dec rate with respect to integration time. This analysis shows that the reviewer’s understanding of the spectral lock-in detection is incorrect. The frequency-bin bandwidth (i.e., frequency resolution) is given by $1/T_{seg}$, where T_{seg} is the duration of a single time window in the spectral lock-in detection process, as described in Appendix A. As explained in Fig. S6,

the duration of one time-window in our experiments was 8 μs, resulting in a frequency resolution of 125 kHz (not 1.5 MHz).

Also, the reviewer is correct that we use the magnitude of the voltage (after sampling and separating the frequency components) in our spectral lock-in detection. However, this resemblance to the radiometer technique does not mean that we have used a radiometer method. As described in detail in Point 1 and Appendix A, our method is different from the radiometer method, and unlike the radiometer method, which provides a 5 dB/dec reduction in noise power as a function of integration time, our method provides a 10 dB/dec reduction in noise as a function of integration time. As shown in the detailed description in Appendix A, the main difference between the radiometer method and our method is that we first perform spectral separation and demodulation directly on the sampled voltage (since we have a linear detector) and convert the lock-in-detected voltage to power in the subsequent steps. In contrast, the radiometer method directly applies the lock-in detection process to the measured power data through square-law detection, leading to a different sensitivity dependence on integration time.

b) I requested a comparison with the author's previous publication (Ref.). 48. The author replied that the results are not directly comparable and that the differences could be up to four orders of magnitude. I see two issues here: i) The result from Ref. 48 are still many orders of magnitude better and should be used as the benchmark in your comparison.

We did not say that "the differences could be up to four orders of magnitude." The reviewer has miscalculated this. Please see the exact response we provided to the reviewer in the last round, after which we present the calculations: *"The work mentioned by the reviewer is a receiver developed for precision astronomy observations. It is a setup that includes optical alignment stabilization, optical intensity stabilization, and frequency stabilization (PLL) modules, and operates under long integration times—none of which are used in the detector presented in this manuscript. As described in Ref. 48, the 2.5 K noise temperature was achieved over a 320-second integration time, which is 5000 times longer than the 64 ms integration time used for the longest measurement in Fig. S6c. Since our detector sensitivity is enhanced at a rate of 10 dB/decade as a function of integration time, this 5000× increase in integration time translates to a 5000× increase in sensitivity. Furthermore, the 2.5 K noise temperature in Ref. 48 was achieved using a PLL that significantly narrows the optical pump linewidth, enabling spectral lock-in detection with a 125 kHz bandwidth—12 times smaller than the 1.5 MHz bandwidth used in our experiments, which do not utilize a PLL. This narrower bandwidth provides a 12× reduction in noise power while maintaining the same signal power through spectral lock-in detection, resulting in another 12× enhancement in sensitivity. In addition, the optical alignment stabilization and optical intensity stabilization used to achieve the 2.5 K noise temperature each contribute 1–2 orders of magnitude reduction in the standard deviation of the detected signal over a 64 ms integration time. Therefore, depending on the operating environment (e.g., temperature fluctuations, laser current fluctuations, vibrations, etc.), the optical alignment and intensity stabilization techniques used in Ref. 48 could provide an additional sensitivity enhancement of 2–4 orders of magnitude compared to the detector demonstrated in our manuscript, which does not incorporate any of these features."*

The following table summarizes the enhancement factors in Ref. 48. It estimates that a 7–9 orders of magnitude enhancement in sensitivity could be offered by the receiver described in Ref. 48.

Table 1. Summary of the sensitivity enhancement factors in Ref. 48

Enhancement factor due to increased integration time	5000
Enhancement due to narrower optical pump linewidth	12
Enhancement due to optical intensity stabilization	10-100
Enhancement due to optical alignment stabilization	10-100
Total estimated enhancement (without considering the enhancement due to laser drift reduction)	$6x10^6$ – $6x10^8$

ii) Why not conduct the same measurement under similar conditions to establish at least one direct, unquestionable point of comparison?

The 809 nm lasers used to pump our QW PIN detectors are mechanically tuned, making it impossible to incorporate them into a phase-locked loop (PLL) to prevent linewidth broadening and frequency drift. This is because a PLL requires an electrical feedback signal to control the laser wavelength, and a mechanically tuned laser cannot be adjusted within such a loop. As a result, the IF frequency shifts and IF spectrum broadens over time, making very long integration times (e.g., 320 s) ineffective for sensitivity enhancement. Similarly, shifts in the IF frequency and its spectral broadening prevent the use of spectral lock-in detection with narrow bandwidths without incurring signal loss. In addition, the intensity and alignment stabilization methods reported in Ref. 48 were implemented using free-space setups that included reference beam branches monitored to provide feedback to the laser and to the piezo stage on which the detector was mounted, thereby optimizing laser power and detector alignment. Such free-space intensity and alignment stabilization schemes are not compatible with the chip described in this manuscript, since variations in optical coupling from free space to the chip would introduce significant uncertainty in the feedback signal levels, preventing effective stabilization. While the required building blocks for phase, frequency, or intensity stabilization aimed at further sensitivity enhancement have not been incorporated into our current chips, they can be implemented using PIC foundry processes, as demonstrated by other groups. Therefore, although we cannot apply the same sensitivity-enhancement mechanisms to the current chips, all of these approaches will be feasible in future implementations.

c) The authors claim that constant sensitivity over integration time only applies to coherent homodyne measurements and is not relevant to their receiver. I disagree. For a heterodyne detector, sensitivity is independent of integration time, just as it is for any electronic frequency extender used with a spectrum analyser. The situation is different for direct detectors without a local oscillator, such as some Schottky detectors or Golay cells. These effectively integrate the power of all frequencies and cannot provide spectral (frequency and phase) information.

As described in Point 1, Appendix A provides a mathematical proof that the sensitivity of our detector is enhanced at a 10 dB/dec rate with respect to the integration time, which is also supported by our experimental results. Also, as described in Point 3, the statement that "*the receiver's input-referred noise power spectral density must not scale with the integration time*" is not always valid and depends on many device/system parameters.

d) I requested the input-referred noise without any temporal integration. The authors state that this is included in Fig. S6b. However, it seems that this graph already incorporates some form of integration or time averaging, and possibly even the lock-in stage. This would affect the power spectral density values. I was also unable to find information on whether 64 μs is used as the window length for the short-time FFT. A frequency resolution of 1.5 MHz would correspond to a window of just ~0.64 μs. In conclusion, it seems that the 64 μs refer to the lock-in period, indicating that lock-in demodulation of the magnitude is applied. Therefore, this is not the actual power spectral density that I requested.

We would like to clarify that the reviewer did not request "*the input-referred noise without any temporal integration*" in the previous rounds of review. In the previous round of review, the reviewer requested that we calculate our device sensitivity by measuring the output noise, calculating the spectral power density, and referring the spectral power density to the input using the conversion loss. We stated that exactly the same approach was taken and illustrated in Fig. S6: "*We have used exactly the same procedure outlined by the reviewer. The receiver output noise (i.e., the receiver output in the absence of an input signal) is measured using an ADC, and the results are shown in Fig. S6b. The spectral power density values in the presence of an input signal are measured and calculated using spectral lock-in detection without any subtraction, as shown in Fig. S6c. Finally, the input-referred noise (sensitivity) values are calculated from the spectral power density and conversion loss, as shown in Fig. S6f.*"

As discussed in Point 4, we do not understand what the reviewer is referring to when asking for the "*input-referred noise without any temporal integration*." This comment may stem from the reviewer's misunderstanding of our detection technique, assuming that we start with a measurement that provides unprocessed output power spectral density, which then goes through integration in later steps (as stated in one of the reviewer's comments).

Unlike the reviewer's assumption, and as described in Appendix A, we start by sampling the detector output using an ADC over multiple time windows, and we need at least one time window to generate our first set of signal spectra. Stated differently, the very first signal spectrum is obtained from the collective data within a time window of duration T_{seg}, which cannot be zero.

From the reviewer's comment, it appears that the reviewer is looking for the input-referred noise corresponding to one time window with a duration of T_{seg}. Under this assumption, we have updated Fig. S6 and included the graphs and data for one time window with a duration of T_{seg} = 8 μs. We stated "*the 8 µs data correspond to measurements captured over an 8 µs time window without applying lock-in detection*". Please note that our frequency resolution is not 1.5 MHz; rather, it is 1/ T_{seg} = 125 kHz. We combine the powers in multiple frequency bins to extract the data over a 1.5 MHz bandwidth, as illustrated in Fig. S6d. Although our system provides a spectral resolution of 125 kHz, we selected a 1.5 MHz bandwidth to extract the output signal because the combined laser linewidth and frequency drift spread the down-converted power over an approximately 1.5 MHz bandwidth during a 64 ms integration time (see Fig. S6). Employing lasers with higher frequency stability and narrower linewidths, or phase-locking/referencing the lasers, would reduce the effective linewidth of the down-converted signal. Under such conditions, the output power could be extracted from a narrower frequency band (<1.5 MHz), leading to lower noise power and improved detection sensitivity.

3) Terahertz Emitter:
a) In their rebuttal, the authors wrote that "...Ref. 21 ([4]) and [5] use uni-traveling-carrier photodiodes that are not compatible with standard photonic integrated circuits (PICs) offered by foundries. Ref. 21 used a fabrication process that required three epitaxial growth steps. Quoting from the authors of Ref. 21 ([4]) and [6]: "The main drawbacks of this approach are that it requires a dedicated fabrication process flow."" However, in Ref. 21 [4], UTC-PDs are implemented alongside SOAs, lasers, waveguides and modulators on a single chip, while in Ref. [7], PIN-PDs are implemented with SOAs and waveguides. In Ref. 21 [4], PDs based on UTC-PDs are described, and it is mentioned that „This process flow uses fabrication steps that are compatible with the integration of MQW active sections and the realization of shallow ridge laser and SOA waveguides.“ [8]. Furthermore, typical foundry platforms for photonic integration commonly use multiple epitaxial growth steps. This means that UTC-PDs are just as compatible with photonic integration as PIN diodes. There are also further examples of photonic integrated UTC-PDs, such as [9].

The reviewer may have overlooked the meaning of **compatibility with standard photonic integrated circuits (PICs)**. When a process is compatible with standard PICs, the user tapes out a chip with a foundry that has already fixed the semiconductor substrate layers, their thickness, composition, and doping; the substrate growth process; the waveguide etching process and etch depth; the planarization dielectric type and thickness; the metal layers and their thickness; and other related parameters. These specifications have been optimized by the foundry through years of refinement and are therefore highly repeatable across many fabrication runs. In addition, the process design kit (PDK) for the main building blocks is already provided by the foundry and many users employ it to fabricate their chips side-by-side in shared multi-project wafer (MpW) runs. The user only designs the layout based on the fixed process and available PDKs. If the user wants to make any changes to these specifications, then the process is not compatible with a standard PIC process and a custom dedicated fabrication run must be arranged.

There are several disadvantages to custom dedicated runs: (1) new process development and optimization must be performed, making custom runs more risky, time-consuming, expensive, and less repeatable; (2) the PDKs provided by the foundry may not be compatible with the custom process specifications; (3) many previously developed PICs that could complement the new designs and enable more complex systems cannot be used with the custom run; and (4) because of the specialized nature of custom runs, there are far fewer customers interested in them, making these runs very expensive and associated with long lead times. A tangible example illustrating the difference between a standard foundry process and a custom process is comparing a tape-out with TSMC using one of their standard CMOS MPW runs versus a custom run to fabricate the same CMOS chip at TSMC with a small

process change (e.g., modifying the gate material or channel doping). Changes as small as these can increase the cost from approximately $20k to $5M and extend the lead time by an order of magnitude.

The examples provided by the reviewer cannot be fabricated using standard PIC foundry processes, as their authors have also pointed out in their papers. Quoting from one of the references listed by the reviewer: "*The main drawbacks of this approach are that it requires a dedicated fabrication process flow.*"

Contrary to the reviewer's statement, adding extra process steps to regrow UTCPD layers on a foundry wafer and fabricating a UTCPD alongside other devices on that wafer does not make the process a standard PIC foundry process. Moreover, using a process flow that includes steps compatible with the integration of MQW active sections and the realization of shallow-ridge laser and SOA waveguides does not make the process a standard PIC foundry process. Our results show that high-performance terahertz emitters and detectors can be fabricated on the same QW PIN PIC substrate without requiring any regrowth or bonding processes.

b) The authors wrote, "Beyond scalability issues, these integrated terahertz optoelectronics schemes have been limited to generating signals up to ~100 GHz without demonstrating high-frequency signal detection." However, this is not accurate, as [7] demonstrated an emitter with SOAs and four PIN-PDs that emitted at 210 GHz.

Unfortunately, the reviewer is quoting our statement out of context. This statement was provided when discussing chips that have lasers integrated with emitters and other components on the same chip, as can be seen from the full statement: "*Meanwhile, demonstrated integrated terahertz optoelectronics have relied on complex fabrication processes, such as multiple epitaxial growth steps [21] to achieve the monolithic integration of uni-traveling-carrier photodiodes with optical pump lasers, semiconductor optical amplifiers (SOAs), and modulators, as well as bonding multiple III-V semiconductor stacks onto a silicon photonics chip [22] to heterogeneously integrate lasers, modulators, and photodiodes. Beyond scalability issues, these integrated terahertz optoelectronics schemes have been limited to generating signals up to ~100 GHz without demonstrating high-frequency signal detection.*"

While Ref. [7] mentioned by the reviewer is very impressive work, it does not include lasers integrated with the terahertz emitters on the same chip, as illustrated in their die photo and stated by the authors: "*optical heterodyne signal is shined into the waveguide*." Stated differently, Ref. [7] does not fit the context of our statement questioned by the reviewer. To prevent similar confusion, we have modified the statement as follows, while citing Ref. [7] suggested by the reviewer:

"*Meanwhile, demonstrated integrated terahertz optoelectronics have relied on complex fabrication processes, such as multiple epitaxial growth steps [21, ref] to achieve the monolithic integration of uni-traveling-carrier photodiodes with optical pump lasers, semiconductor optical amplifiers (SOAs), and/or modulators, as well as bonding multiple III-V semiconductor stacks onto a silicon photonics chip [22] to heterogeneously integrate lasers, modulators, and photodiodes. Beyond scalability issues, these integrated terahertz optoelectronics schemes have been limited in the operation frequency without demonstrating high-frequency signal detection.*"

c) The authors replied that "...monolithic integration with other active/passive optoelectronic components on the same chip is the unique advantage of our devices." As previously mentioned, there are multiple publications demonstrating the integration of multiple active and passive optoelectronic components on a single chip [4, 7, 9]. There are also publications demonstrating photonic integration-compatible emitters, receivers, or both [5, 8, 9, 10, 11].

Unfortunately, the reviewer is quoting our statement out of context. This was not a general statement, but was provided in our response to the reviewer when he was comparing our emitter with other gain-enhanced THz emitters in the literature, which use additional bulky amplifiers after their optical pump lasers (Fig. 3). When comparing our emitter with those publications, we stated: "*monolithic integration with other active/passive*

optoelectronic components on the same chip is the unique advantage of our devices. This includes optical gain on the same chip without the need to use an additional bulky amplifier that have been used for other reported work."

[1] D. M. Pozar, Microwave Engineering, 4th ed. Hoboken, NJ: John Wiley & Sons, Inc., 2012.
[2] N. Wang, S. Cakmakyapan, Y. J. Lin, H. Javadi, and M. Jarrahi, "Room-temperature heterodyne terahertz detection with quantum-level sensitivity," Nature Astronomy 2019 3:11, vol. 3, no. 11, pp. 977–982, July 2019, doi: 10.1038/s41550-019-0828-6.
[3] K. Rohlfs and T. L. Wilson, Tools of Radio Astronomy. in Astronomy and Astrophysics Library. Berlin, Heidelberg: Springer, 1996. doi: 10.1007/978-3-662-03266-4.
[4] G. Carpintero et al., "Microwave Photonic Integrated Circuits for Millimeter-Wave Wireless Communications," Journal of Lightwave Technology, vol. 32, no. 20, pp. 3495–3501, Oct. 2014, doi: 10.1109/JLT.2014.2321573.
[5] C. C. Renaud, M. Natrella, C. Graham, J. Seddon, F. Van Dijk, and A. J. Seeds, "Antenna Integrated THz Uni-Traveling Carrier Photodiodes," IEEE Journal of Selected Topics in Quantum Electronics, vol. 24, no. 2, pp. 1–11, Mar. 2018, doi: 10.1109/JSTQE.2017.2725444.
[6] G. Carpintero, R. C. Guzmán, C. Gordón, G. Kervella, M. Chitoui, and F. Van Dijk, "Photonic Integrated Circuits for Radio-Frequency Signal Generation," Journal of Lightwave Technology, vol. 34, no. 2, pp. 508–515, Jan. 2016, doi: 10.1109/JLT.2015.2511040.
[7] G. Carpintero et al., "Photonic-Enabled Terahertz Phase Arrays Using Dielectric Rod Waveguides for 6G Wireless Communications," in 2024 IEEE/MTT-S International Microwave Symposium - IMS 2024, June 2024, pp. 74–77. doi: 10.1109/IMS40175.2024.10600234.
[8] E. Rouvalis et al., "High-speed photodiodes for InP-based photonic integrated circuits," Opt. Express, OE, vol. 20, no. 8, pp. 9172–9177, Apr. 2012, doi: 10.1364/OE.20.009172.
[9] M. Anagnosti et al., "Record Gain x Bandwidth (6.1 THz) Monolithically Integrated SOA-UTC Photoreceiver for 100-Gbit/s Applications," Journal of Lightwave Technology, vol. 33, no. 6, pp. 1186–1190, Mar. 2015, doi: 10.1109/JLT.2014.2372816.
[10] E. Rouvalis et al., "170 GHz Photodiodes for InP-based photonic integrated circuits," in IEEE Photonics Conference 2012, Sept. 2012, pp. 88–89. doi: 10.1109/IPCon.2012.6358502.
[11] M. Deumer et al., "Waveguide-integrated photoconductive THz receivers," in 2022 47th International Conference on Infrared, Millimeter and Terahertz Waves (IRMMW-THz), Aug. 2022, pp. 1–2. doi: 10.1109/IRMMW-THz50927.2022.9895580.

Reviewer #3 (Remarks to the Author):

As the authors state, I have no doubt that this work is highly important and has the potential to enable future miniaturization and cost reduction of terahertz systems. However, at this stage, the study is still in the phase of establishing the core technological components, and in my view it does not, by itself, generate a broad interest. Therefore, my conclusion remains unchanged: it would be more appropriate to submit this work to a more specialized journal rather than Nature Communications.

We thank the reviewer for acknowledging the importance of our work and its potential to enable future miniaturization and cost reduction of terahertz systems. Given the potential of the introduced monolithically integrated terahertz optoelectronics platform to transform terahertz radar, communication, imaging, and sensing systems from expensive, bulky, laboratory-grade setups into scalable, compact, and low-cost microchips for everyday use, thereby impacting many aspects of people's lives, we strongly believe that our manuscript is very well suited for the highly interdisciplinary and broad readership of Nature Communications.

We thank the reviewers for their valuable feedback, which significantly improved the clarity and quality of our manuscript. Our responses to the reviewer comments are shown in blue, and the revised material in the manuscript and supplementary information is shown in red.

Reviewer #5 (Remarks to the Author):

The authors report on the development and demonstration of a single QW PIN structure allowing various modes of operation. This is an important milestone on the way towards the integration of optical, terahertz and electronic properties on a single chip built from possibly universal components – which is a topic and achievement certainly appropriate for Nature Communications.

The manuscript essentially builds on and synthesizes numerous long-term developments: while QW PIN structure is the core component, the various functionalities address quite a number of branches of photonics (optical amplification, terahertz generation using photo-mixing, terahertz down-conversion etc.) – it is hard to imagine the unbelievable amount of work behind all these studies. The manuscript thus contains an extreme amount of useful and interesting information.

We thank the reviewer for attesting to the importance of the findings of our manuscript and acknowledging the significant amount of work behind these studies.

A serious weakness of the manuscript is the unfocused style of the presentation – it took me quite some time to just decipher its main message.

We thank the reviewer for this important comment and fully agree that clarity and focus in the presentation are critical. We believe the extent of the content covered in this manuscript may have contributed to this issue. To improve readability and better guide the reader to the main message, we have refined key paragraphs in the latter part of the Introduction and at the beginning of the Results section to more explicitly state the main contribution and distinguish it from prior work:

Here, we demonstrate that interband photomixing in quantum well (QW) PIN photodiodes—previously unexplored for terahertz applications—provides an efficient and scalable mechanism for both terahertz generation and detection. By leveraging gain-enhanced photomixing within a platform inherently compatible with photonic integrated circuits (PICs), this approach overcomes key limitations of existing photomixers in efficiency, sensitivity, and integrability. In a prototype fabricated on a GaAs/AlGaAs QW PIC substrate, we demonstrate frequency-tunable terahertz generation and detection across the 100–500 GHz range, achieving both higher terahertz generation efficiency and improved terahertz detection sensitivity compared to the state-of-the-art[23-25]. The compatibility of QW PIN photodiodes with commercially available PIC foundry processes further enables monolithic integration of terahertz sources, detectors, and optical components on a single chip.

Another issue is that there are many unsupported or undeveloped claims.

We thank the reviewer for pointing out the missing justification in different parts of the manuscript. We have revised the manuscript and supplementary information to provide the missing information, as listed below:

For example, at lines 105 - 107 it is written that “The values of tau_QW and tau_trans depend on the reverse bias voltage, as well as the composition and thickness of the heterostructure layers forming the

QW PIN photodiode." - since these dependencies are used throughout the manuscript, I would expect a formula or a reference.

Reference 29 of the manuscript, which was a preprint when we submitted this manuscript and has now been published (Zhao, Y. & Jarrahi, M., Ultrafast carrier dynamics in multiple quantum well p-i-n photodiodes, J. Appl. Phys. 139, 015701, 2026), provides a detailed analysis of ultrafast carrier dynamics in QW PIN photodiodes, including analytical and numerical calculations of τ_{QW} and τ_{trans}. We have added a summary of this information to the supplementary information (Fig. S1), where we also describe the dependence of τ_{QW} and τ_{trans} on the reverse bias voltage, as well as the composition and thickness of the heterostructure layers forming the QW PIN photodiode:

Under increasing reverse bias, the electric field across the intrinsic region becomes stronger, tilting the potential barriers and enhancing carrier tunneling out of the wells. As a result, the carrier escape time from the QWs (τ_{QW}) becomes shorter at higher bias voltages. Similarly, the carrier transit time (τ_{trans}) decreases with reverse bias because carriers are accelerated more rapidly and quickly reach their saturation velocity, reducing the time required to traverse the depletion region. The heterostructure composition and layer thickness also play a central role. Higher barrier heights set by material composition (e.g., higher Al content in AlGaAs) and thicker barriers increase confinement, leading to longer escape times, while thinner wells and barriers, along with lower band offsets, reduce the escape time by facilitating tunneling. Effective mass differences between materials further influence escape rates by modifying the tunneling probability. The total thickness of the intrinsic region directly determines the transit distance; thus, thicker intrinsic layers increase τ_{trans}, whereas thinner regions reduce it. In addition, material-dependent saturation velocities and the presence of heterojunction barriers (which can temporarily trap carriers) further modulate the effective transit time. Overall, higher reverse bias and thinner, lower-barrier heterostructures lead to shorter τ_{QW} and τ_{trans}, while thicker layers and higher band offsets increase these characteristic times.

Particular numerical values falling from a clear sky excessively appear throughout the entire manuscript. For example, at lines 108 - 111 it is written that "Figure 1a presents the theoretically predicted frequency response of a reverse-biased QW PIN photodiode with an RC time constant of 1.55 ps, fabricated on the GaAs/AlGaAs QW PIC substrate used in our demonstrations.

We have added detailed photomixer modeling and frequency response analysis to the supplementary information (Figs. S1–S6), which provide detailed numerical, analytical, and experimental results leading to the estimated RC time constant of 1.55 ps and the theoretically predicted frequency response shown in Fig. 1a.

The substrate structure, detailed in Supplementary Fig. S1, provides estimated saturation velocities of 0.72×10^7 and 0.8×10^7 cm/s..." - it is not clear how any of these three physical values was determined.

References 30 and 31 have been added to the revised manuscript, where the saturation velocities of the utilized material are reported.

Altogether, although the manuscript still needs quite some improvement (even at the price of its expansion), the subject is really important to deserve communication to a broader audience.

Once again, we thank the reviewer for pointing out the missing information and unclear aspects of our manuscript, which have helped us significantly improve its quality and clarity.

Reviewer #6 (Remarks to the Author):

The manuscript from the Jarrahi group reports on a new method of THz or sub-THz frequency generation by photomixing in GaAs/AlGaAs semiconductor heterostructures. The results comprise higher efficiency, lower noise figures as compared to other photomixing approaches as well as straightforward on-chip integration owing to the monolithic semiconductor process. The device architecture even allows for versatile operation modes ranging from detection, phase or amplitude modulation to the aforementioned main purpose, photomixing.

The principle of the device is straightforward and based on established semiconductor physics, and as such nothing new. However, the technological relevance of this work is very high owing to the potentially cost-effective implementation of THz devices from communications to sensing to industry and medical applications. Moreover, it has potential in research owing to the possible broadband operation with the potential to generate short pulses. In my view, the work suits Nature Communications in principle.

We thank the reviewer for acknowledging the high technological impact of the findings of our manuscript and the potential of the introduced technology for various applications.

However, I would like to see two points to be addressed to broaden the scope of the work:
- Mechanism. What (quantum?) model would describe the charge carrier dynamics and enable one to deduce suitable current densities? The authors refer to a preprint paper with their own model. How would, e.g., modulation doping of the structure affect the electron transport?

The carrier dynamics in our photomixers are described by a semiclassical quantum-escape + transport model: carriers photogenerated in the QWs escape via field-assisted tunneling and thermionic emission (with field-modified QW density of states and energy-dependent tunneling probability included), then transit across the intrinsic and depleted cladding regions at saturation velocity; the induced external current is obtained via the Shockley–Ramo theorem. Using the escape times, heterojunction trap times, and transit times from this model, we calculate the photocurrent density and frequency response. We have now added an explicit statement of these modeling assumptions to the manuscript:

Under reverse bias, which governs interband photon absorption within the QWs via the quantum-confined Stark effect[28] – the photo-generated carriers escape from the QWs and drift across the intrinsic region (Fig. 1a inset). We model carrier dynamics using a semiclassical quantum-escape formulation: photocarriers generated in the mth QW escape the well by a combination of field-assisted tunneling and thermionic emission (computed using the field-modified density of states and energy-dependent transmission probability) and are then accelerated to saturation velocity across the intrinsic and depleted cladding layers[29]. The resulting induced current is obtained from the carrier motion using the Shockley-Ramo theorem, which accounts for both conduction and displacement currents (see Supplementary Figs. S1). This framework maps material and device parameters (e.g., barrier heights, doping densities, depletion widths, and layer thicknesses) to carrier escape, trapping, and transit times and therefore to a predicted photocurrent. As a result, the contribution of photo-generated electrons and holes in the mth QW traveling across the intrinsic region at high frequencies can be approximated as[29]

$$I_{e,m}(\omega) \approx \frac{z_m}{L+W_n+W_p} \times \frac{1}{1+j\omega\tau_{RC}} \times \frac{qSN_m}{1+j\omega\tau_{QW}^{e}} \times sinc\left(\frac{\omega\tau_{trans}^{e}}{2}\right) e^{-\frac{j\omega\tau_{trans}^{e}}{2}} \quad (1)$$

$$I_{h,m}(\omega) \approx \frac{L-z_m}{L+W_n+W_p} \times \frac{1}{1+j\omega\tau_{RC}} \times \frac{qSN_m}{1+j\omega\tau_{QW}^{h}} \times sinc\left(\frac{\omega\tau_{trans}^{h}}{2}\right) e^{-\frac{j\omega\tau_{trans}^{h}}{2}} \quad (2)$$

where z_m is the distance from the mth well to the N-side, L, W_n and W_p are the thicknesses of the intrinsic region, depleted N-region, and depleted P-region, respectively. $\mathcal{S}$ denotes the device area, and N_m is the number of photo-generated electrons and holes in the mth QW. τ_{QW}^{e} and τ_{QW}^{h} correspond to the electron and hole escape times from the QW respectively, whereas τ_{trans}^{e} and τ_{trans}^{h} denote the electron and hole transit times through the intrinsic region. Additionally, τ_{RC} represents the RC time constant of the device. The total frequency response is obtained by summing the current contributions from all QWs.

Reference 29 of the manuscript, which was a preprint when we submitted this manuscript and has now been published (Zhao, Y. & Jarrahi, M., Ultrafast carrier dynamics in multiple quantum well p-i-n photodiodes, J. Appl. Phys. 139, 015701, 2026), provides a detailed analysis of ultrafast carrier dynamics in QW PIN photodiodes, including the calculation of current densities. We have added a summary of this information to the supplementary information (Figs. S1 and S3), where we also describe the impact of doping on carrier transport and frequency response:

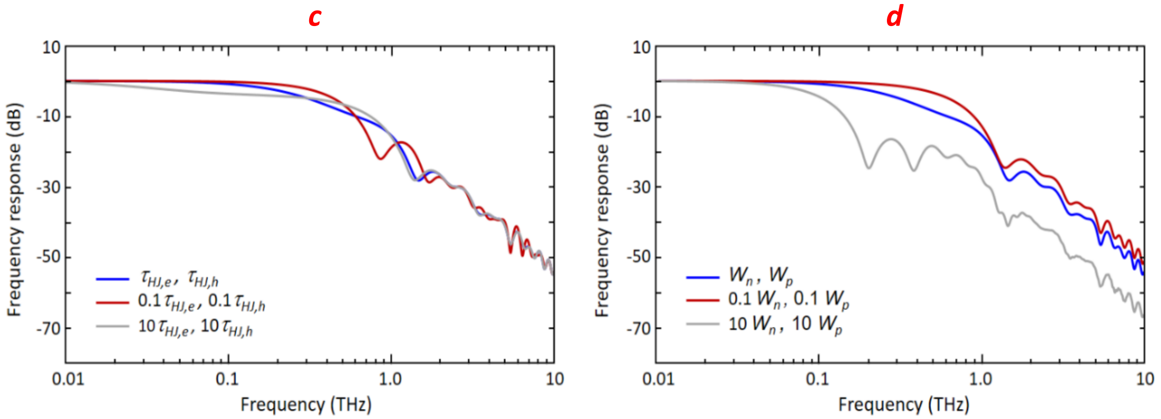

***Fig. S3. c**, The impact of heterojunction trap time on the frequency response is studied, indicating a negligible dependence. This is because the heterojunction trap time only affects the current contribution from the photocarriers moving inside the depleted n- and p-cladding layers with depletion widths W_n and W_p, respectively. In the structure studied, the doping level is high enough to keep the depletion width in the cladding layer relatively small compared to the intrinsic region, resulting in minimal effect on the frequency response. **d,** The impact of cladding layer doping on the frequency response is studied. Using lighter doping in the cladding layers extends the depletion width, which increases the carrier transit time and results in a steeper frequency roll-off.*

- Scalability / material platform. The strength as well as THz bandwidth achievable with the photocurrent depend crucially on the intrinsic properties of the charge carriers and thus semiconductor material as well as the geometry of the structure. Which other materials may be suitable and for what reasons?

We thank the reviewer for pointing out the important aspect of the platform's scalability with respect to other materials and their impact on performance. To address this important point, we have added the following to the discussion section of the revised manuscript:

While the present demonstration is based on a GaAs/AlGaAs quantum well platform, the underlying gain-enhanced interband photomixing mechanism is broadly applicable to other semiconductor material systems. The achievable terahertz bandwidth and efficiency are primarily governed by carrier escape time, carrier transit velocity, and device parasitics, which in turn depend on the effective mass, band offsets, and saturation velocity of the semiconductor. Materials with higher carrier saturation velocities and lower effective masses, such as InGaAs/InAlAs or InP-based QW structures, could enable faster carrier transport and extended terahertz bandwidth. Emerging material systems, including GaN/AlGaN, may further extend the operational range toward higher frequencies due to their large band offsets and potentially ultrafast carrier dynamics. These considerations highlight that the proposed MITO platform is

not limited to a specific material system but can be adapted to different semiconductor platforms depending on the targeted performance metrics and integration requirements.

Regarding the authors' exchange with Referee 1, my assessment is that the Referee's original concerns were justified and, as stated by the Referee, have been addressed to a sizeable extent with rigor. The Referee has some more open questions and I am a bit surprised by the confrontational nature of the authors' responses. The Referee's statement for example that a normalized noise figure (units: W/Hz) should not depend on measurement time is correct and I would like to see a solid response to that.
On the question of noise and power measurements in general, my view is that this is certainly important when benchmarking something intended as a device architecture against existing concepts. I feel however that questions of scaling and universality as raised by myself above are more central.

We are glad that our response to Referee 1's comments has addressed their concerns to a significant extent. Here, we address the Referee's statement that a normalized noise figure (units: W/Hz) should not depend on measurement time. Referee 1's statement is based on the fact that the sensitivity (units: W/Hz) of the detectors in Refs. 38 and 39 (Refs. 40 and 41 in the revised manuscript) does not improve as a function of integration time when performing conventional lock-in detection under the operating conditions used in those manuscripts. Under those conditions, the detected noise power is reduced at a 10 dB/dec rate as a function of integration time. However, the detected signal power is also reduced at a 10 dB/dec rate as a function of integration time, since increasing the integration time effectively performs low-pass filtering. As a result, the references mentioned by Referee 1 cannot improve the signal-to-noise ratio (sensitivity) by increasing the integration time, since both signal and noise power decrease at a 10 dB/dec rate as a function of integration time. In contrast to the conventional lock-in detection process, we have used a spectral lock-in detection process, which preserves the frequency information of the signal. Therefore, we can reduce the noise power at a 10 dB/dec rate as a function of integration time (similar to the conventional lock-in detection process), while maintaining the same signal power at each frequency. This allows enhancement of the signal-to-noise ratio (sensitivity) at a 10 dB/dec rate as a function of integration time. A detailed mathematical proof of this sensitivity enhancement process is appended to our response (please see the following pages). In addition, experimental evidence of this dependence is provided in our manuscript (Fig. 2h inset and Fig. S10) and Ref. 50.

Appendix A: Conventional and Spectral Lock-in Detection Principles

Conventional lock-in detection improves the signal-to-noise ratio (SNR) of a modulated signal by eliminating a significant portion of the broadband noise; however, it does not preserve spectral information. Here, we describe a spectral lock-in detection technique that enhances the SNR of the captured spectra while retaining spectral information.

We begin with an overview of the conventional lock-in detection process. The process starts by modulating the target signal, which shifts its frequency components to higher frequencies where flicker noise is significantly reduced (Fig. 1a). The modulated signal is then demodulated by multiplication with the in-phase (I) and quadrature (Q) components of the reference modulation signal, $V_{ref}(t)$. This operation shifts the spectrum back to low frequencies, allowing the original signal amplitude and phase to be recovered (Fig. 1b). To further reduce noise, the I and Q signals are integrated over an integration time τ. This integration effectively acts as a low-pass filter, suppressing out-of-band noise with a bandwidth inversely proportional to τ, as illustrated in Fig. 1c.

At short integration times, when the low-pass filter bandwidth is larger than the signal bandwidth, increasing the integration time reduces the noise power at a rate of 10 dB/dec with respect to integration time while maintaining the signal power, resulting in an SNR improvement of 10 dB/dec. However, at longer integration times, when the low-pass filter bandwidth becomes smaller than the signal bandwidth, both the noise power and the signal power decrease at a rate of 10 dB/dec, preventing any further enhancement in SNR with increasing integration time. In either regime, the lock-in–detected output represents a superposition of all filtered frequency components and does not preserve the spectral information of the original signal.

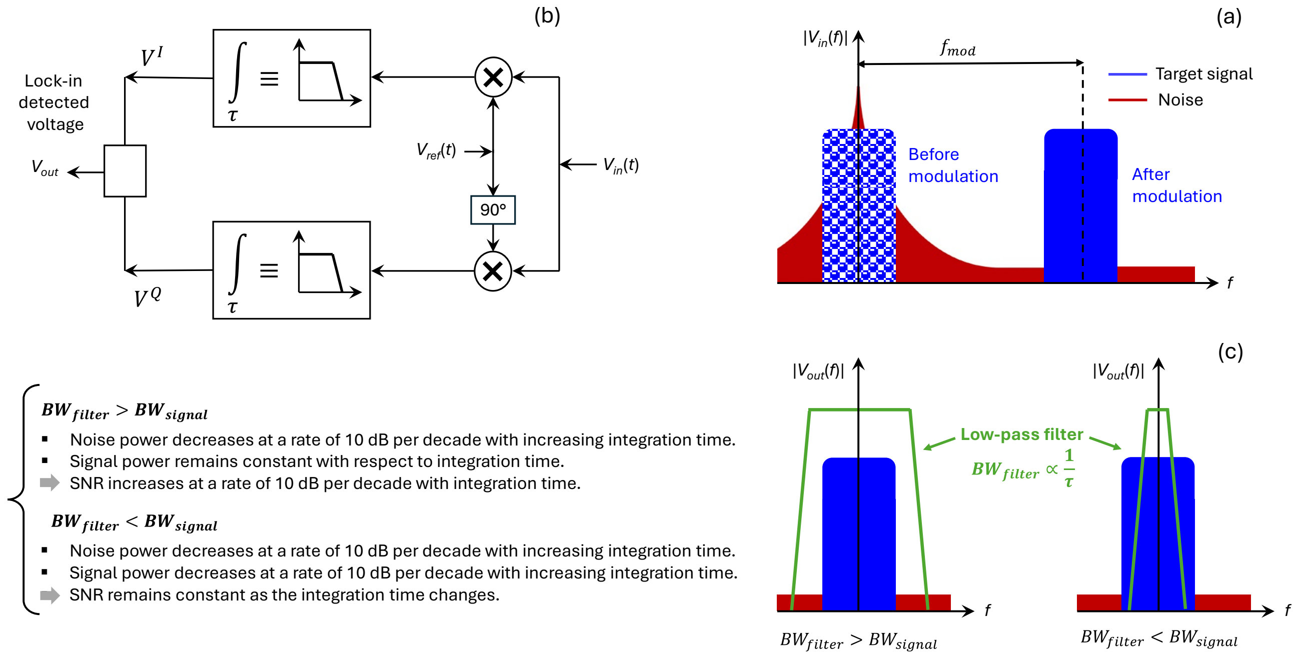

Fig.1. (a) Signal modulation shifts the frequency components by the modulation frequency, f_{mod}. (b) Block diagram of the conventional lock-in detection process applied to a modulated signal. (c) Spectrum of the lock-in–detected signal, illustrating the effective low-pass filtering enabled by integration.

Next, we introduce the spectral lock-in detection technique both conceptually and analytically, and explain how it reduces the noise power at a rate of 10 dB/dec with respect to integration time while maintaining the signal power. This results in an SNR improvement of 10 dB/dec while preserving frequency information. All discussions and conclusions are supported by mathematical proof.

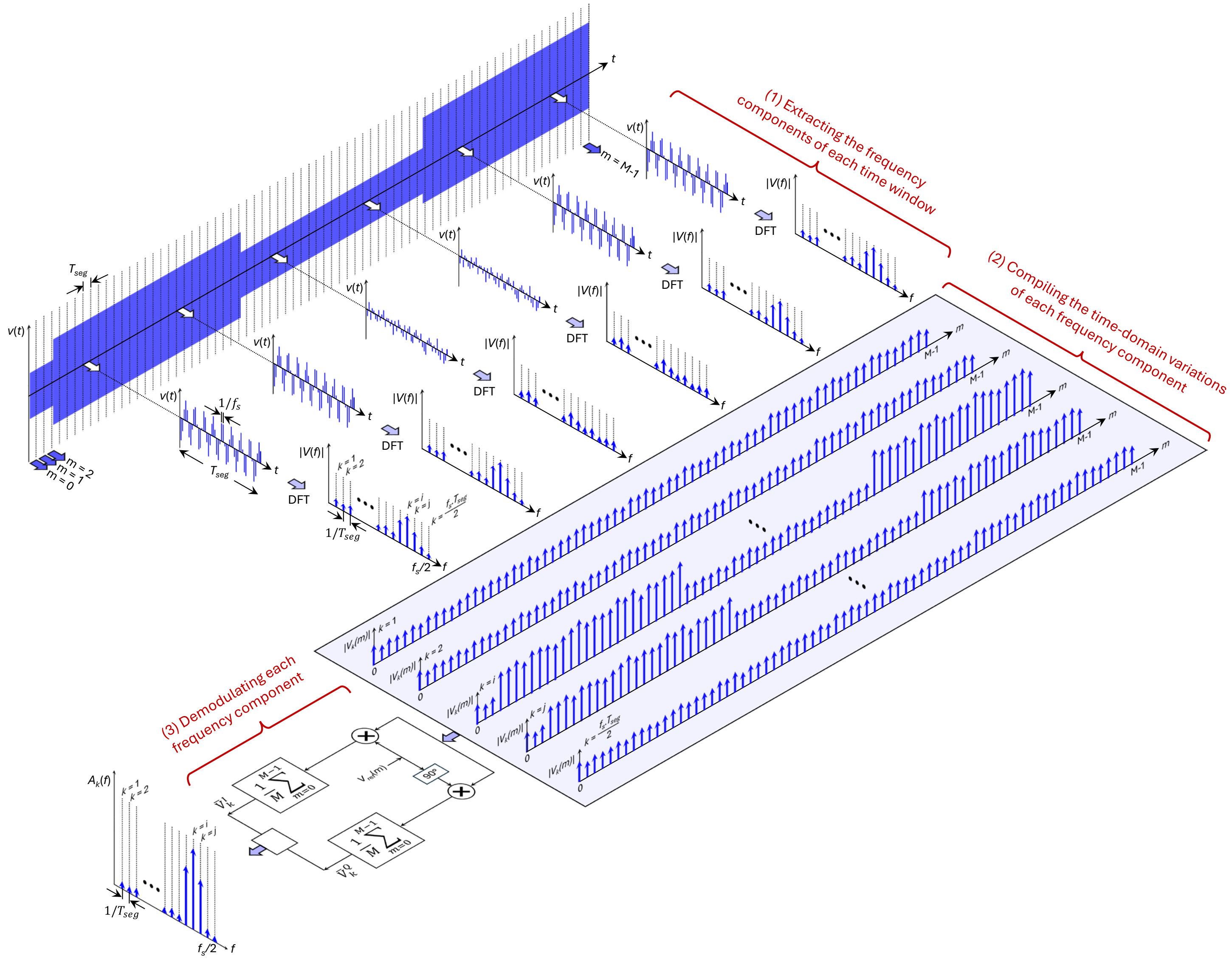

Fig.2. Schematic diagram and principles of spectral lock-in detection.

Figure 2 illustrates the principle of spectral lock-in detection. The raw time-domain modulated signal, sampled by an analog-to-digital converter (ADC), is segmented into multiple time windows. For each window, the frequency components are extracted using a discrete Fourier transform (DFT). The resulting time sequence of each frequency component across all windows is then compiled and demodulated using conventional lock-in detection. The final lock-in–detected spectrum is obtained by combining the lock-in outputs of all frequency components.

Assuming that the frequency components of the target signal remain constant over the integration time, the signal bandwidth of each frequency bin approaches an infinitesimally small value (i.e., a delta function). Consequently, the demodulated signal power at each frequency bin remains constant, independent of the

integration time. Because conventional lock-in detection is applied to the time stream of each individual frequency bin, the noise power of the lock-in–detected bins decreases at a rate of 10 dB/dec with increasing integration time. This leads to a corresponding signal-to-noise ratio (SNR) improvement of 10 dB/dec, while preserving frequency-domain information. A mathematical proof of this performance is provided below.

The sampled voltage by the ADC, $v(t)$, is the superposition of the modulated signal, $v_s(t)$, and unmodulated noise, $v_n(t)$:

$$v(t) = v_s(t) + v_n(t) \tag{1}$$

The spectral lock-in detection process starts by dividing the sampled voltage into time-windows of duration T_{seg}, each containing $N = f_s T_{seg}$ samples.

$$v[m,l] = v\left(\frac{mT_{seg} + l}{f_s}\right) \tag{2}$$

where f_s is the ADC sampling rate, *m* is the window index ($m = 0, 1, \ldots, M - 1$), and *l* is the sample index within each window ($l = 0, 1, \ldots, N - 1$).

For each time-window, *m*, the frequency components of the voltage are calculated using a discrete Fourier Transform. The voltage at frequency bin *k* is:

$$V_k(m) = \frac{1}{N}\sum_{l=0}^{N-1} v[m,l]e^{-\frac{i2\pi kl}{N}} \tag{3}$$

Because $v[m,l] = v_s[m,l] + v_n[m,l]$, the signal and noise contributions to $V_k(m)$ are:

$$S_k(m) = \frac{1}{N}\sum_{l=0}^{N-1} v_s[m,l]e^{-\frac{i2\pi kl}{N}} \tag{4}$$

$$N_k(m) = \frac{1}{N}\sum_{l=0}^{N-1} v_n[m,l]e^{-\frac{i2\pi kl}{N}} \tag{5}$$

For white Gaussian noise $v_n(t)$ with zero mean and spectral power density N_0, (units V^2/Hz), the variance of each discrete sample $v_n[m,l]$ is equal to the spectral power density of the white noise integrated over the sampling Nyquist band:

$$\sigma_n^2 = N_0 f_s \tag{6}$$

Therefore, the variance of the noise voltage in each frequency bin, which has a bandwidth of $1/T_{seg}$, is calculated as:

$$N_k(m) \sim \mathcal{N}(0, \sigma_0^2),\ \sigma_0^2 = \frac{1}{N}\sigma_n^2 = \frac{N_0}{T_{seg}} \tag{7}$$

After extracting the frequency components of the voltage for each window, *m*, the magnitude of the voltage at frequency bin *k* is calculated to account for possible phase incoherence in the signal.

$$|V_k(m)| = \left|\frac{A_k}{2}\left[1 + sgn\left(\omega_{mod} mT_{seg}\right)\right]e^{i\varphi_k(m)} + N_k(m)\right| = \left|\frac{A_k}{2}\left[1 + sgn\left(\omega_{mod} mT_{seg}\right)\right] + \tilde{N}_k(m)\right| \tag{8}$$

where A_k and $\varphi_k(m)$ represent the voltage amplitude and phase at the kth frequency bin. Since $N_k(m)$ is a circularly symmetric complex Gaussian random variable (i.e., isotropic in phase), multiplying it by any phasor $e^{-i\varphi_k(m)}$ does not change its distribution. Specifically, $\widetilde{N_k}(m) = N_k(m)e^{-i\varphi_k(m)}$ has the same statistics $\mathcal{N}(0, \sigma_0^2)$.

Next, the magnitude of the voltage at frequency bin k is demodulated through multiplication by the in-phase (I) and quadrature (Q) components of the modulation reference: $sgn\left[sin\left(\omega_{mod} mT_{seg} + \varphi_{mod}\right)\right]$ and $sgn\left[cos\left(\omega_{mod} mT_{seg} + \varphi_{mod}\right)\right]$, where $\varphi_{mod} \in (\pi, -\pi)$ is the phase difference between the modulated signal and the modulation reference.

$$\tilde{V}_k^I(m) = \left|\frac{A_k}{2}\left[1 + sgn\left[sin\left(\omega_{mod} mT_{seg}\right)\right]\right] + \tilde{N}_k(m)\right| sgn\left[sin\left(\omega_{mod} mT_{seg} + \varphi_{mod}\right)\right] \sigma_n^2 = N_0 f_s \tag{9}$$

$$\tilde{V}_k^Q(m) = \left|\frac{A_k}{2}\left[1 + sgn\left[sin\left(\omega_{mod} mT_{seg}\right)\right]\right] + \tilde{N}_k(m)\right| sgn\left[cos\left(\omega_{mod} mT_{seg} + \varphi_{mod}\right)\right] \tag{10}$$

Next, the demodulated samples $\tilde{V}_k^I(m)$ and $\tilde{V}_k^Q(m)$ are averaged over M window frames, corresponding to a total integration time of $T = MT_{seg}$, to obtain the lock-in detected voltages in each frequency bin k:

$$\bar{V}_k^I(T) = \frac{1}{M} \sum_{m=0}^{M-1} \tilde{V}_k^I(m) \tag{11}$$

$$\bar{V}_k^Q(T) = \frac{1}{M} \sum_{m=0}^{M-1} \tilde{V}_k^Q(m) \tag{12}$$

In the following section, we prove that

(i) The mean demodulated voltages $\bar{V}_k^I(T)$ and $\bar{V}_k^Q(T)$ at each frequency bin k remain constant, while the corresponding noise voltage is reduced as a function of integration time at a rate of 5 dB/dec; and

(ii) The mean power corresponding to the demodulated voltages at each frequency bin k remains constant, while the corresponding noise power is reduced as a function of integration time at a rate of 10 dB/dec.

<u>Proof:</u>

We perform a statistical analysis of the lock-in detected voltages $\bar{V}_k^I(T)$ and $\bar{V}_k^Q(T)$ by calculating their mean and standard deviation. We first calculate the mean values $\langle \bar{V}_k^I(T) \rangle$ and $\langle \bar{V}_k^Q(T) \rangle$.

With a probability of $\frac{\pi - |\varphi_{mod}|}{2\pi}$, $\tilde{V}_k^I(m) = |A_k + \tilde{N}_k(m)|$; with a probability of $\frac{|\varphi_{mod}|}{2\pi}$, $\tilde{V}_k^I(m) = -|A_k + \tilde{N}_k(m)|$; with a probability of $\frac{|\varphi_{mod}|}{2\pi}$, $\tilde{V}_k^I(m) = |\tilde{N}_k(m)|$; and with a probability of $\frac{\pi - |\varphi_{mod}|}{2\pi}$, $\tilde{V}_k^I(m) = -|\tilde{N}_k(m)|$. Therefore, the mean value of $\bar{V}_k^I(T)$ is calculated as:

$$\langle \bar{V}_k^I(T) \rangle = \left(\frac{1}{2} - \frac{|\varphi_{mod}|}{\pi}\right)\left[\frac{1}{M}\sum_{m=0}^{M-1} \langle |A_k + \tilde{N}_k(m)| \rangle - \frac{1}{M}\sum_{m=0}^{M-1} \langle |\tilde{N}_k(m)| \rangle\right] \tag{13}$$

With a probability of $\frac{\pi-\left|\varphi_{mod}+\frac{\pi}{2}\right|}{2\pi}$, $\tilde{V}_k^Q(m) = \left|A_k + \tilde{N}_k(m)\right|$; with a probability of $\frac{\left|\varphi_{mod}+\frac{\pi}{2}\right|}{2\pi}$, $\tilde{V}_k^Q(m) = -\left|A_k + \tilde{N}_k(m)\right|$; with a probability of $\frac{\left|\varphi_{mod}+\frac{\pi}{2}\right|}{2\pi}$, $\tilde{V}_k^Q(m) = \left|\tilde{N}_k(m)\right|$; and with a probability of $\frac{\pi-\left|\varphi_{mod}+\frac{\pi}{2}\right|}{2\pi}$, $\tilde{V}_k^Q(m) = -\left|\tilde{N}_k(m)\right|$. Therefore, the mean value of $\bar{V}_k^Q(T)$ is calculated as:

$$\langle \bar{V}_k^Q(T)\rangle = \left(\frac{1}{2} - \frac{\left|\varphi_{mod}+\frac{\pi}{2}\right|}{\pi}\right)\left[\frac{1}{M}\sum_{m=0}^{M-1}\langle\left|A_k + \tilde{N}_k(m)\right|\rangle - \frac{1}{M}\sum_{m=0}^{M-1}\langle\left|\tilde{N}_k(m)\right|\rangle\right] \tag{14}$$

For $X \sim \mathcal{N}(\mu, \sigma_0^2)$, $\langle|X|\rangle = \sigma_0\sqrt{\frac{2}{\pi}}exp\left(-\frac{\mu^2}{2\sigma_0^2}\right) + \mu erf\left(\frac{\mu}{\sigma_0\sqrt{2}}\right)$. Therefore,

$$\langle|A_k + N_k(m)|\rangle - \langle|N_k(m)|\rangle = A_k erf\left(\frac{A_k}{\sigma_0\sqrt{2}}\right) - \sigma_0\sqrt{\frac{2}{\pi}}\left(1 - exp\left(-\frac{A_k^2}{2\sigma_0^2}\right)\right) \tag{15}$$

While this expression should be evaluated numerically for general cases, to obtain a conceptual understanding of the spectral lock-in detection process, we consider a scenario in which the original signal is distinguishable relative to the noise ($A_k \gg \sigma_0$) in order to obtain an analytical solution. Under this condition, $\langle \bar{V}_k^I(T)\rangle$ and $\langle \bar{V}_k^Q(T)\rangle$ are approximated as:

$$\langle \bar{V}_k^I(T)\rangle \approx A_k\left(\frac{1}{2} - \frac{|\varphi_{mod}|}{\pi}\right) \text{ and } \langle \bar{V}_k^Q(T)\rangle \approx A_k\left(\frac{1}{2} - \frac{\left|\varphi_{mod}+\frac{\pi}{2}\right|}{\pi}\right) \tag{16}$$

where $\varphi_{mod} \in (\pi, -\pi)$.

Therefore, φ_{mod} and A_k can be calculated from the lock-in-detected voltages $\bar{V}_k^I(T)$ and $\bar{V}_k^Q(T)$. Please note that the mean lock-in-detected voltage values $\langle \bar{V}_k^I(T)\rangle$ and $\langle \bar{V}_k^Q(T)\rangle$, which yield the signal voltage at each frequency bin (A_k) are independent of the integration time and remain constant as a function of integration time, as indicated by the mathematical proof.

Next, we calculate the standard deviation of $\bar{V}_k^I(T)$ and $\bar{V}_k^Q(T)$.

With a probability of $\frac{1}{2}$, $\tilde{V}_k^I(m) = \pm\left|A_k + \tilde{N}_k(m)\right|$ and with a probability of $\frac{1}{2}$, $\tilde{V}_k^I(m) = \pm\left|\tilde{N}_k(m)\right|$. Similarly, with a probability of $\frac{1}{2}$, $\tilde{V}_k^Q(m) = \pm\left|A_k + \tilde{N}_k(m)\right|$ and with a probability of $\frac{1}{2}$, $\tilde{V}_k^Q(m) = \pm\left|\tilde{N}_k(m)\right|$. Therefore,

$$Var\left(\bar{V}_k^I(T)\right) = Var\left(\bar{V}_k^Q(T)\right) = \frac{1}{2M}Var\left(\left|A_k + \tilde{N}_k(m)\right|\right) + \frac{1}{2M}Var\left(\left|\tilde{N}_k(m)\right|\right) = \frac{\sigma_0^2}{M}\left(1 - \frac{1}{\pi}\right) \tag{17}$$

where

$$Var\left(\left|\tilde{N}_k(m)\right|\right) = \langle\left|\tilde{N}_k(m)\right|^2\rangle - \langle\left|\tilde{N}_k(m)\right|\rangle^2 = \sigma_0^2 - \frac{2}{\pi}\sigma_0^2 = \sigma_0^2\left(1 - \frac{2}{\pi}\right) \tag{18}$$

$$Var\left(\left|A_k + \tilde{N}_k(m)\right|\right) = \langle\left|A_k + \tilde{N}_k(m)\right|^2\rangle - \langle\left|A_k + \tilde{N}_k(m)\right|\rangle^2 = A_k^2 + \sigma_0^2 - A_k^2 = \sigma_0^2 \tag{19}$$

Therefore,

$$Std\left(\bar{V}_k^I(T)\right) = Std\left(\bar{V}_k^Q(T)\right) = \frac{\sigma_0}{\sqrt{M}}\sqrt{1-\frac{1}{\pi}} \tag{20}$$

When calculating the signal voltage at each frequency bin using $\bar{V}_k^I(T)\left(\frac{1}{2}-\frac{|\varphi_{mod}|}{\pi}\right)^{-1}$, the standard deviation of the calculated voltage is $\frac{\sigma_0}{\sqrt{M}}\left(1-\frac{1}{\pi}\right)^{1/2}\left(\frac{1}{2}-\frac{|\varphi_{mod}|}{\pi}\right)^{-1}$. Therefore, while the calculated mean voltage per bin, $A_k \approx \langle\bar{V}_k^I(T)\rangle\left(\frac{1}{2}-\frac{|\varphi_{mod}|}{\pi}\right)^{-1}$, remains constant as a function of integration time, its standard deviation, $\frac{\sigma_0}{\sqrt{M}}\left(1-\frac{1}{\pi}\right)^{1/2}\left(\frac{1}{2}-\frac{|\varphi_{mod}|}{\pi}\right)^{-1}$, is reduced as a function of integration time at a rate of 5 dB/dec.

Similarly, when calculating the mean signal power at each frequency bin using $\frac{1}{2R}\langle\left|\bar{V}_k^I(T)\right|^2\rangle\left|\frac{1}{2}-\frac{|\varphi_{mod}|}{\pi}\right|^{-2}$ (with R = 50 Ω), we obtain:

$$\langle P_k\rangle = \frac{1}{2R}A_k^2 + \frac{1}{2R}\frac{\sigma_0^2}{M}\left(1-\frac{1}{\pi}\right)\left(\frac{1}{2}-\frac{|\varphi_{mod}|}{\pi}\right)^{-2} \tag{21}$$

This expression includes the original signal power, $\frac{1}{2R}A_k^2$, plus a noise power of $\frac{1}{2R}\frac{\sigma_0^2}{M}\left(1-\frac{1}{\pi}\right)\left(\frac{1}{2}-\frac{|\varphi_{mod}|}{\pi}\right)^{-2}$, which is reduced as a function of integration time at a rate of 10 dB/dec.

3. Previous publications

I noticed that there are three conference papers by the authors covering this topic already. I leave it to the editor if that is interfering with this publication or not: [9], [10], [11]

Manuscript	[11]	[9]	[10]
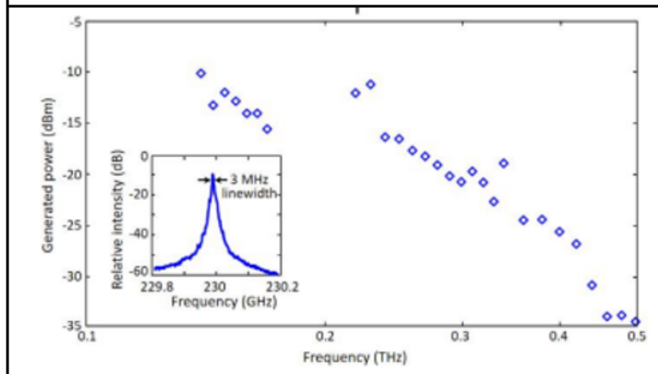	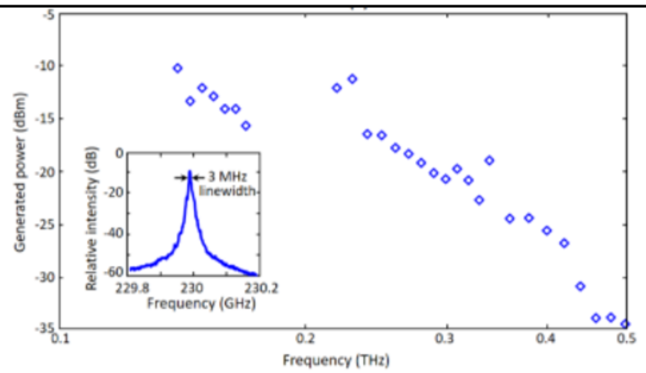	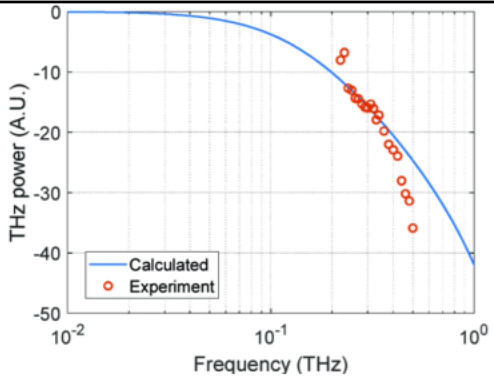	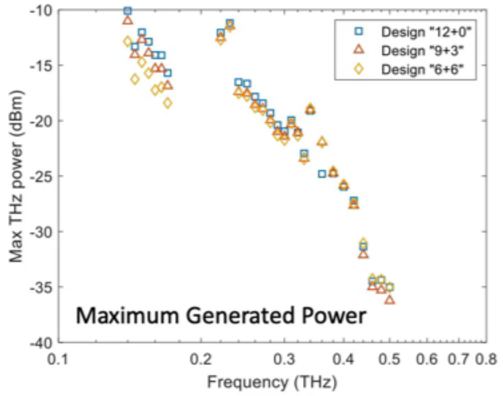
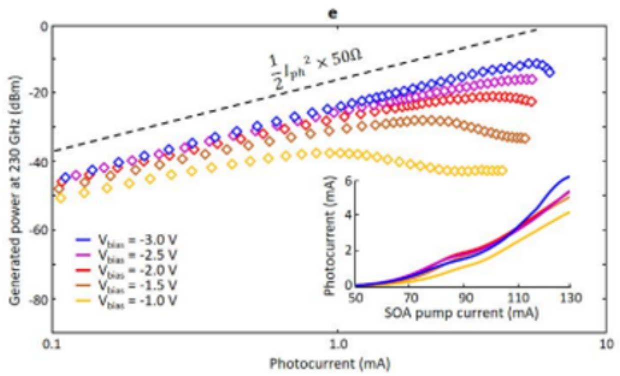	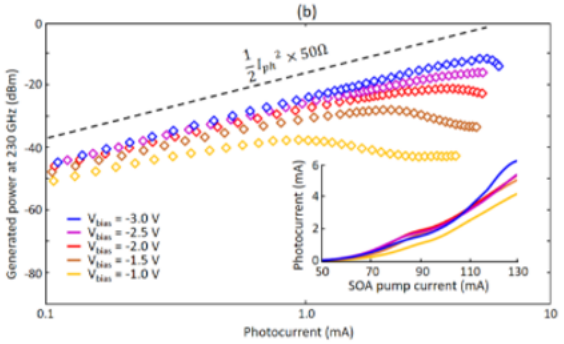	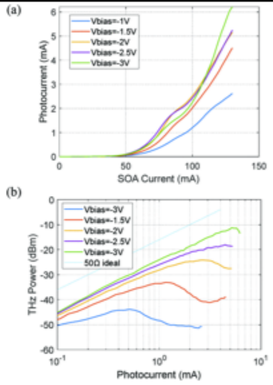	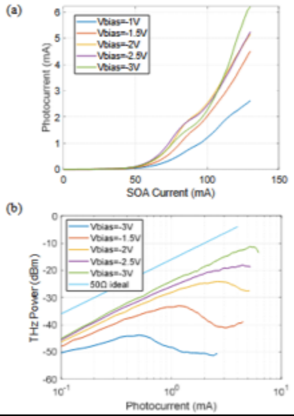
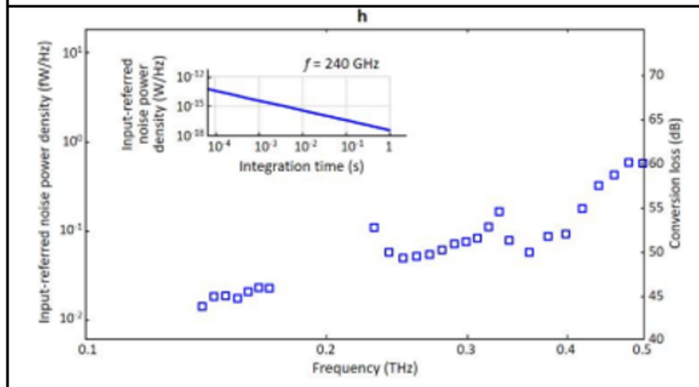	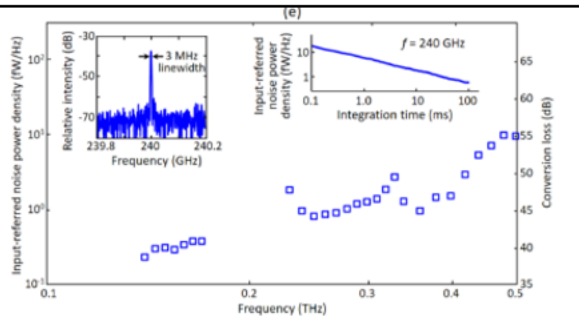	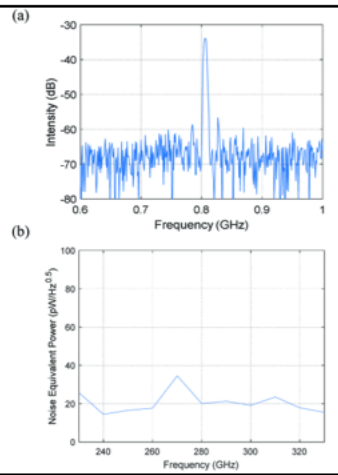	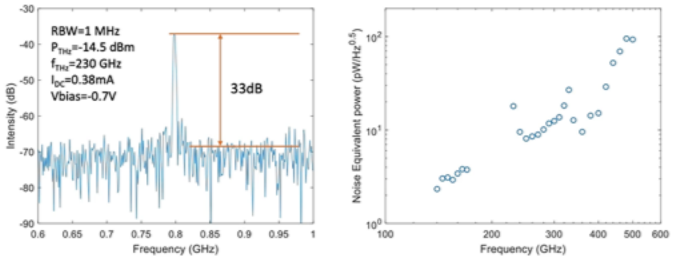
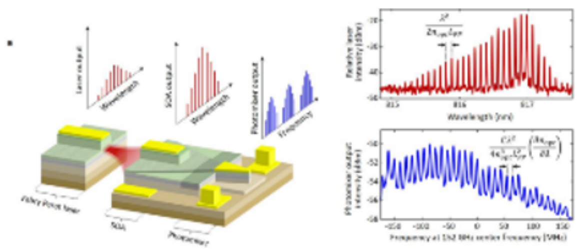	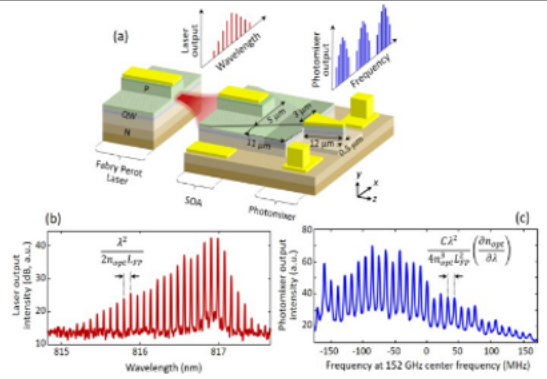	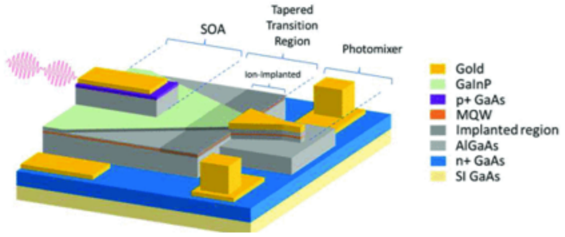	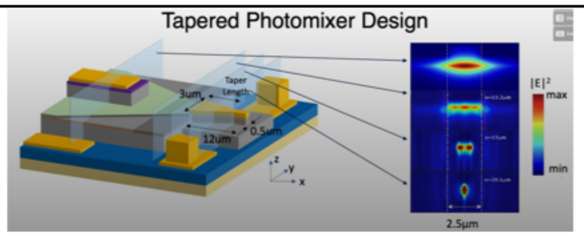
